# IPEV: identification of prokaryotic and eukaryotic virus-derived sequences in virome using deep learning

**DOI:** 10.1093/gigascience/giae018

**Published:** 2024-04-22

**Authors:** Hengchuang Yin, Shufang Wu, Jie Tan, Qian Guo, Mo Li, Jinyuan Guo, Yaqi Wang, Xiaoqing Jiang, Huaiqiu Zhu

**Affiliations:** Department of Biomedical Engineering, College of Future Technology, and Center for Quantitative Biology, Peking University, Beijing 100871, China; Department of Biomedical Engineering, College of Future Technology, and Center for Quantitative Biology, Peking University, Beijing 100871, China; Department of Biomedical Engineering, College of Future Technology, and Center for Quantitative Biology, Peking University, Beijing 100871, China; Department of Biomedical Engineering, College of Future Technology, and Center for Quantitative Biology, Peking University, Beijing 100871, China; Department of Biomedical Engineering, College of Future Technology, and Center for Quantitative Biology, Peking University, Beijing 100871, China; School of Life Sciences, Peking University, Beijing 100871, China; Department of Biomedical Engineering, College of Future Technology, and Center for Quantitative Biology, Peking University, Beijing 100871, China; Department of Biomedical Engineering, Georgia Institute of Technology and Emory University, Atlanta, GA 30332, USA; Department of Biomedical Engineering, College of Future Technology, and Center for Quantitative Biology, Peking University, Beijing 100871, China; Department of Biomedical Engineering, College of Future Technology, and Center for Quantitative Biology, Peking University, Beijing 100871, China; Beijing Institute of Genomics, Chinese Academy of Sciences, and China National Center for Bioinformation, Beijing 100101, China; Department of Biomedical Engineering, College of Future Technology, and Center for Quantitative Biology, Peking University, Beijing 100871, China; School of Life Sciences, Peking University, Beijing 100871, China; Department of Biomedical Engineering, Georgia Institute of Technology and Emory University, Atlanta, GA 30332, USA

## Abstract

**Background:**

The virome obtained through virus-like particle enrichment contains a mixture of prokaryotic and eukaryotic virus-derived fragments. Accurate identification and classification of these elements are crucial to understanding their roles and functions in microbial communities. However, the rapid mutation rates of viral genomes pose challenges in developing high-performance tools for classification, potentially limiting downstream analyses.

**Findings:**

We present IPEV, a novel method to distinguish prokaryotic and eukaryotic viruses in viromes, with a 2-dimensional convolutional neural network combining trinucleotide pair relative distance and frequency. Cross-validation assessments of IPEV demonstrate its state-of-the-art precision, significantly improving the F1-score by approximately 22% on an independent test set compared to existing methods when query viruses share less than 30% sequence similarity with known viruses. Furthermore, IPEV outperforms other methods in accuracy on marine and gut virome samples based on annotations by sequence alignments. IPEV reduces runtime by at most 1,225 times compared to existing methods under the same computing configuration. We also utilized IPEV to analyze longitudinal samples and found that the gut virome exhibits a higher degree of temporal stability than previously observed in persistent personal viromes, providing novel insights into the resilience of the gut virome in individuals.

**Conclusions:**

IPEV is a high-performance, user-friendly tool that assists biologists in identifying and classifying prokaryotic and eukaryotic viruses within viromes. The tool is available at https://github.com/basehc/IPEV.

## Introduction

Viruses and virus-like particles (VLPs) are abundant and diverse biological entities on Earth. It is estimated that there are approximately 10^31^ viral particles [1], showcasing their pervasive nature. Notably, even in human feces, there can be as many as 10^9^ VLPs per gram, further emphasizing their prevalence and ubiquity [2, 3]. The advent of next-generation sequencing (NGS) technology has revolutionized virome studies, enabling the discovery of novel viruses and significantly advancing our understanding of their potential influence on both environmental and human body microbiomes [4–7]. Nonetheless, it is essential to note that enriched sample approaches carry the risk of losing valuable host or environmental information [8], potentially leading to inaccurate virus host identification and constraining subsequent analyses. A noteworthy example is our previous study, in which we found a strong correlation between alterations in the proportion of temperate phages within the gut virome and the occurrence of ulcerative colitis in patients [9]. Similarly, the significance of the eukaryotic virome should not be overlooked when examining the virome, as it is believed to play a vital role in both host health and disease [10–14]. Recent studies have shed light on intricate trans-kingdom interactions involving eukaryotic viruses, bacteria, and the host within the intestinal ecosystem [15–18]. Obviously, these analyses first require the precise differentiation of eukaryotic viruses from prokaryotic viral sequences, and it is beneficial for gaining a comprehensive understanding of the viral landscape [9, 15].

However, the precise identification of prokaryotic and eukaryotic viruses poses a significant challenge owing to the highly diverse and fast-mutating genetic elements within the virome. Furthermore, the limitations of assembly tools arise from factors such as mutations, recombination events, and often low or uneven sequencing coverage across the viral genome [19]. The abundance of short and frequently inadequate reads further complicates the task of virus identification. Moreover, the absence of a well-conserved genetic marker such as the bacterial 16S ribosomal RNA gene presents a barrier in constructing a phylogenetic tree to effectively differentiate between eukaryotic and prokaryotic viruses [20]. Virus identification typically involves aligning sequences against known viruses in genomic repositories, such as the NCBI Taxonomy Databases [21] and European Nucleotide Archive (ENA) [22]. Despite the rapid increase in viral sequences, the number of viral reference sequences in public reference databases is still limited, constraining the effectiveness of sequence-based alignment methods. For instance, it has been estimated that there are millions of viral species, but the International Committee on Taxonomy of Viruses has recognized only 11,273 species to date [23].

Currently, some computational tools for identifying viruses have been introduced in metagenomes, such as HoPhage [24], iPHoP [25, 26], WIsH [27], CHERRY [28], PHP [29], and VHM-Net [30], designed to assign the host for a given phage contig using sequence similarity search or *ab initio* identification. Some tools, like DeePhage [9], PHACTS [31], and PhagePred [32], have been developed to answer questions about the lifestyles of phages. Other tools, like PPR-Meta [33], DeepVirFinder [34], VIBRANT [35], vConTACT2 [36], VirSorter [37], and the most recent version, VirSorter2 [38], can be used to identify viruses from metagenomics data. While analyzing and classifying phages in virome data is a current focus, it is important to note that eukaryotic viruses also play a critical role in influencing host immunity and disease phenotypes by infecting host cells and interacting with the bacterial microbiome through trans-kingdom interactions. However, existing methods for analyzing virome data are limited in distinguishing eukaryotic viruses from viromes, assigning hosts, and classifying them accurately [39]. Some methods, such as the Host Taxon Predictor (HTP) [40], can be used to bridge the gap, differentiating between phages and eukaryotic viruses based on sequence information and nucleic acid type (such as DNA or RNA). Given that HTP’s performance is highly dependent on the nucleic acid type used and that some experimental protocols for virome research may result in mixed datasets containing both DNA and RNA sequences [41], it might be challenging to accurately determine the origin of these sequences. This uncertainty could, in our observations, influence the performance of HTP in classifying viruses.

In this article, we present the IPEV (Identify Prokaryotic and Eukaryotic Virus-derived sequences), a high-performance, user-friendly tool for differentiating prokaryotic and eukaryotic viruses from virome sequence fragments. To achieve high performance on short viral sequences, we developed a 2-dimensional (2D) convolutional neural network (CNN) based on a sequence pattern matrix using the Sequence Graph Transform (SGT) model. Cross-validation tests demonstrate that IPEV significantly outperforms related methods in terms of F1-score metrics by at most 21.1% while requiring only 1/50th the time of HTP in the same computing environment. We also designed various homology layouts for independent sets based on known sequence data to assess IPEV’s generalization capabilities. IPEV outperforms HTP k-Nearest Neighbors (KNN) by approximately 22% in terms of the F1-score on an independent set with highly imbalanced labels when the sequence identity between the training and independent test sets is less than 30%. IPEV’s evaluations of marine virome samples are better than HTP, with much more accurate results. We applied IPEV to analyze the longitudinal gut virome data from 10 healthy individuals over 12 months and achieved the best performance in at least 90% of the samples compared to other methods. Our analysis revealed that the gut virome exhibits temporal stability beyond that observed in persistent personal viromes, thus enhancing our understanding of gut virome stability in individuals.

## Materials and Methods

### Dataset construction

Without accurately host-annotated virome datasets that could serve as benchmarks, we generated simulated datasets based on well-annotated complete virus genomes. First, we downloaded the taxonomy ID list of viruses and corresponding host lineages from Virus-Host DB [42] and genome sequences from the NCBI database [43] on 31 October 2021. As a result, we established our first dataset, referred to as dataset 1, which contains 11,022 eukaryotic virus genomes and 5,051 prokaryotic virus genomes (of which 113 are attributed to archaeal viruses). To enhance the model’s generalizability, we incorporated additional data from the Reference Viral Database (RVDB) [44] and 25,644 eukaryotic sequences along with 5,598 prokaryotic sequences from IMG/VR v4 [45], collectively termed dataset 2. Details regarding the data inclusion criteria are outlined in the Supplementary Materials and Methods. Dataset 1 is sourced from reference sequences and manually curated with credible host annotations, while dataset 2 is not. Therefore, based on genomes, we used all viruses in dataset 2 and 10,000 eukaryotic and 4,000 prokaryotic viruses in dataset 1 randomly divided for 5-fold cross-validation, while the remaining subset served as an independent test set for assessing generalizability.

Considering the limitations of current mainstream sequencing technologies and the length constraints of assembled contigs, we simulated 4 contig length groups (A–D) using MetaSim v0.9.1 (RRID:SCR_011940) [46] with “exact” preset and “Uniform” distribution types. The contig length groups were as follows: group A (100–400 bp), group B (400–800 bp), group C (800–1,200 bp), and group D (1,200–1,800 bp). The specific contig numbers can be found in Supplementary Tables S1 and S2. We evaluated IPEV’s generalization ability using an independent test set of 1,022 eukaryotic and 1,051 prokaryotic virus sequences. Using MetaSim, we totally generated 20,000 contigs and ensured low similarity against the training set using BLASTn (v2.7.1), following the above length groups. We generated 6 low homology independent test sets (datasets I1–I6) with varying query coverage and identity thresholds relative to the training set. The number of corresponding prokaryotic and eukaryotic virus contigs in each independent test set can be found in Supplementary Tables S5 and S6, respectively.

We evaluated the effect of sequencing errors on IPEV’s performance, and we generated a total of 10,000 contigs (1,200 to 1,800 bp) with 5%, 10%, and 15% sequencing errors based on the independent test set using MetaSim (related details can be found in the Supplementary Materials and Methods). Furthermore, we assessed the capability of the IPEV tool by analyzing protein sequences with functional annotations. We constructed a dataset of 7,384 receptor-binding proteins (RBPs) and corresponding negative samples, which were manually verified. Our selection criteria revolved around methods that are oriented toward function, Gene Ontology annotation, or product description and that feature RBP-related keywords. These protein sequences originated from a wide range of prokaryotic viruses, spanning 7 orders and 28 families, including *Tubulavirales* and *Timlovirales*. To evaluate our model’s efficacy in predicting eukaryotic viruses, we also assembled a collection of 7 experimentally confirmed capping enzymes [47].

We also used a real virome to evaluate IPEV and related tools. We first downloaded a dataset comprising 243 marine virome samples from the ENA (accession number: PRJEB22493 [48, 49]). Besides, we analyzed longitudinal data from Shkoporov et al.’s [50] study to evaluate IPEV’s accuracy and the stability of gut virome data. We retrieved the raw human gut virome dataset from the NCBI Sequence Read Archive (accession number: PRJNA545408). This dataset included 130 virome samples from 10 healthy adults (subjects 916–925) collected over 12 months (T1–T12) through monthly synchronous samplings. We utilized the SPAdes v3.13.0 (RRID:SCR_000131) [51] software to assemble short reads and conducted BLASTn searches against a bacterial database to eliminate bacterial contamination with an e-value of e-5, an identity of 50%, and query coverage of 90%. Our reference bacterial dataset comprised 20,003 complete prokaryotic genomes sourced from the NCBI RefSeq database, comprising 19,629 bacterial genomes and 374 archaeal genomes. Following Shkoporov et al.’s [50] personal persist virome (PPV) definition, we used cd-hit-est (v.4.8.1) with parameters (c 0.8, aS 0.8, d 0, n 5) to cluster decontaminated contigs and defined clusters containing contigs from at least 6 months as PPV clusters. For subject 917, sampled for 11 months, we modified the PPV definition to include contigs appearing in at least 5 months. We aligned the assembled contigs with reference virus sequences using BLASTn to assign virus taxon labels. The potential prokaryotic or eukaryotic viruses were inferred from viral contigs with an e-value less than the cutoff of 1e-4.

### Mathematical model of DNA sequences

In this study, we developed a sequence pattern matrix using the SGT model to extract meaningful information based on the relative positions of trinucleotides [52]. The pattern matrix is a numerical representation of the frequency and order of trinucleotide pairs. We generated a trinucleotide set by combining 3 nucleotides to represent a DNA sequence (*S*) and calculated the weights of trinucleotides *u* and *v* using the following formula:


(1) \begin{eqnarray*}
{\psi }_{uv}\left( s \right) = \frac{{{\sum }_{\forall \left( {l.m} \right)}{e}^{ - k\left| {m - l} \right|}}}{{\left| {{{\mathrm{\Lambda }}}_{uv}\left( s \right)} \right|}}. \end{eqnarray*}

**Figure 1:**
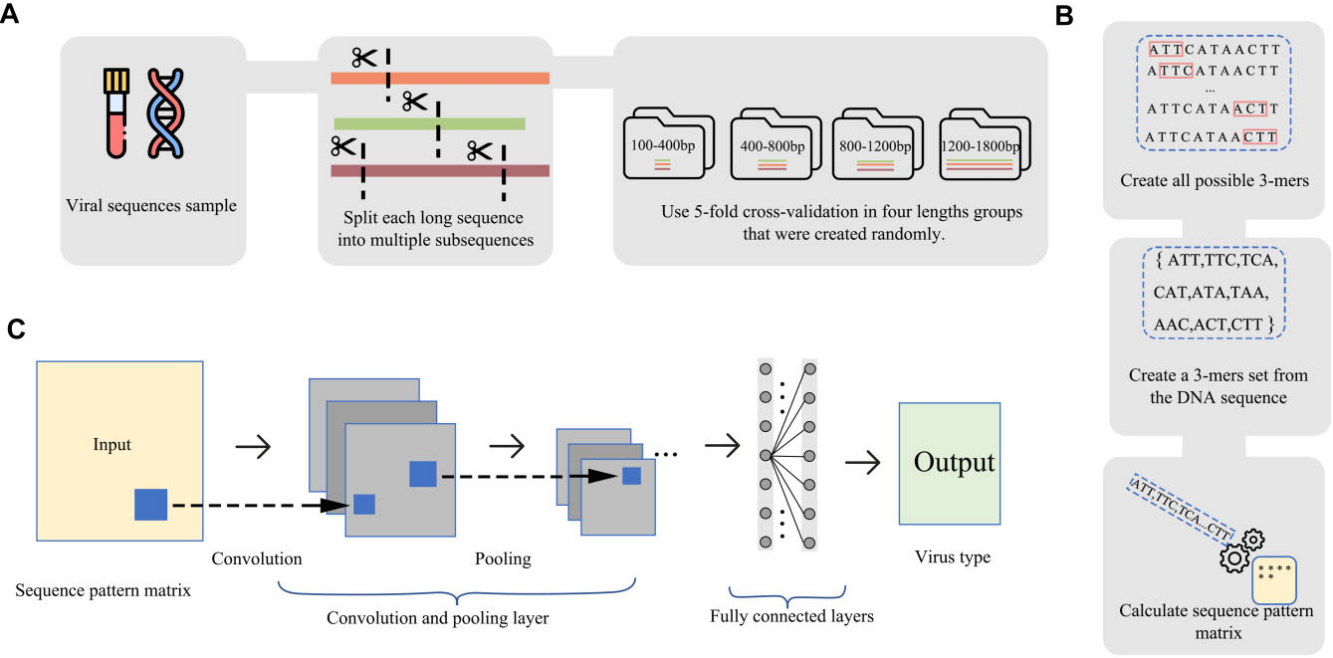
Workflow for extracting the sequence pattern matrix and using a deep learning neural network structure to predict taxon. (A) The virus genomes are initially divided into 5 subsets, and then each subset is simulated to represent 4 groups with different contig lengths. (B) Overlapping trinucleotides are used to represent the virus contigs. For example, if the nucleotides of the viral fragment are “ATTCATAACTT,” the trinucleotide set would consist of “ATT, TTC, TCA, CAT, ATA, TAA, AAC, ACT, CTT.” The trinucleotide set is then converted to a 64 × 64 sequence pattern matrix using a sequence pattern function. (C) The IPEV tool employs a 2D CNN model as the classifier. The CNN model accepts the sequence pattern matrix as input and outputs a 1 × 2 array representing the likelihood of prokaryotic and eukaryotic viruses.

Herein, ${e}^{ - {\mathrm{\kappa }}| {m - l} |}$ represent the weight of a trinucleotide pair of *u* and *v* at the position of *m* and *l*. The relative distances of a trinucleotide pair of *u* and *v* are measured by $| {m - l} |$. $| {{{\mathrm{\Lambda }}}_{uv}} |$ is the size of the set ${{\mathrm{\Lambda }}}_{uv}$. It represents the size of total $( {u,v} )$ pairs in the trinucleotide set of a DNA sequence. A schematic representation of the sequence pattern matrix can be found in Fig. 1B. Finally, the DNA sequence is converted to a 64 × 64 matrix of relationship weights for the trinucleotide pair set.

### Structure of the deep learning neural network

We constructed a 2D CNN to predict taxon information using sequence pattern matrices. The CNN has the following layers: 2 convolution layers (with a 7 × 7 kernel size and the “same” padding), 2 max pooling layers (with a 2 × 2 pooling window), 2 dropout layers, a flatten layer, and 2 fully connected layers followed by a softmax activation function. Here, the Conv2D layer takes the sequence pattern matrix ${\mathrm{I}}$ of dimensions ($L \times L$) as the sequence pattern matrix and generates total ${\mathrm{F}}$ feature maps as output by corresponding ${\mathrm{F}}$ (kernels) of dimensions ${k}_1 \times {k}_2$ with the same padding. Those kernels were used to extract information on the viral sequence. Using the Rectified Linear Unit (ReLU) as the activation function, the Conv2D layer outputs an ${\mathrm{F}} \times {k}_1 \times {k}_2$ matrix ${Y}^C$ and computes the ${{\mathrm{f}}}^{{\mathrm{th}}}$ feature map at the $( {{i}^{{\mathrm{th}}},{j}^{{\mathrm{th}}}} )$ location, and the value is given as


(2) \begin{eqnarray*}
{\left( {Y_{ij}^C} \right)}_f = \ {\mathrm{ReLU}}\left(\sum _{m = 0}^{{k}_1 - 1}\sum _{n = 0}^{{k}_2 - 1}{K}_f\left( {m,n} \right)I\left( {i - m,j - n} \right) + b_f^{\mathrm{C}}\right), \end{eqnarray*}

\begin{eqnarray*}
{\mathrm{for\ }}i,j = 0,1,2, \ldots ,{\mathrm{L}} - 1,{\mathrm{\ f\ = \ }}0,1,2, \ldots ,{\mathrm{F}} - 1. \end{eqnarray*}

where ${K}^f$ and $b_{\mathrm{f}}^{\mathrm{C}}$ are a ${k}_1 \times {k}_2$ weight matrix and a bias of the ${{\mathrm{f}}}^{{\mathrm{th}}}$ kernel. Mainly, the ReLU function mentioned above is defined as follows:


(3) \begin{eqnarray*}
{\mathrm{ReLU}}({\mathrm{x}}) = \ \left\{ {\begin{array}{@{}*{1}{c}@{}} {{\mathrm{x\ }}\quad {\mathrm{if}}\ {\mathrm{x}} \ge 0}\\ {0\quad {\mathrm{if}}\ {\mathrm{x}} < 0} \end{array}} \right.. \end{eqnarray*}

The next layer in the model is a Maxpooling layer, taking the maximum value over an input channel with a pooling size ${\mathrm{S}}1 \times {\mathrm{S}}1$ and a stride size ${\mathrm{S}}2 \times {\mathrm{S}}2$. The padding option is set to “same.” The window is shifted along with each channel independently and can generate F new channels with the size of ${\mathrm{L^{\prime}}} \times {\mathrm{L^{\prime}}}( {{\mathrm{L^{\prime}}} = \lceil{\mathrm{L}}/{\mathrm{S}}2\rceil} )$. The Maxpooling layer outputs an ${\mathrm{L^{\prime}}} \times {\mathrm{L^{\prime}}}\ \times {\mathrm{F}}$ feature matrix ${Y}^M$ and one of the pooling operations for a specific channel at the $( {{i}^{{\mathrm{th}}},{j}^{{\mathrm{th}}}} )$ location was calculated as


(4) \begin{eqnarray*}
Y_{\left( {i,j} \right){\mathrm{,f}}}^{\mathrm{M}} &=& \max (Y_{(i \times {\mathrm{S2,}}j \times {\mathrm{S2),f}}}^{\mathrm{C}},Y_{(i \times {\mathrm{S2 + 1,}}j \times {\mathrm{S2 + 1),f}}}^{\mathrm{C}},Y_{(i \times {\mathrm{S2 + 2,}}j \times {\mathrm{S2 + 2),f}}}^{\mathrm{C}}, \ldots ,\\ &&Y_{(i \times {\mathrm{S2 + S1 - 1,}}j \times {\mathrm{S2 + S1 - 1) - 1,f}}}^{\mathrm{C}}), \end{eqnarray*}

\begin{eqnarray*}
{\mathrm{for\ }}i,j = 0,1,2, \ldots ,{\mathrm{L^{\prime}}} - 1,\ {\mathrm{f}} = 0,1,2, \ldots ,{\mathrm{F}} - 1. \end{eqnarray*}

The features that the neural network learns in the Maxpooling layer are transferred to the Dropout layer. The output ${\tilde{y}}^{{\mathrm{Dp}}}$ is formulated as


(5) \begin{eqnarray*}
{\tilde{y}}^{{\mathrm{Dp}}} = \ \tilde{k}\ {\mathrm{*}}\ {Y}^{\mathrm{M}} \quad \hbox{Where }\tilde{k} \sim B\left( {1,\ {\mathrm{P}}} \right). \end{eqnarray*}

Here ∗ denotes an element-wise product. For any layer *Y*, the drop mask $\tilde{k}$ denotes an independent Bernoulli distribution with random variables, each having a probability *p* of 1. It can effectively reduce overfitting. We employed a Flatten layer to convert all the elements ${{\boldsymbol{Y}}}^{{\mathrm{Dp}}}$ in the tensor into a ${{\boldsymbol{Y}}}^{\mathrm{F}}$ 1-dimensional array one by one. The Dense1 layer uses the ReLU function to output R units. It has an R$\times$F weight matrix ${W}^{{\mathrm{D1}}}$ and an R-dimensional bias vector ${b}^{{\mathrm{D1}}}$. Each output unit is given as follows:


(6) \begin{eqnarray*}
y_{\mathrm{r}}^{D1} = {\mathrm{ReLu}}\left( {\mathop \sum \limits_{{\mathrm{f}} = 0}^{{\mathrm{F}} - 1} W_{{\mathrm{r}},{\mathrm{f}}}^{D1}y_{\mathrm{f}}^{\mathrm{F}} + b_{\mathrm{r}}^{D1}} \right), \quad {\mathrm{\ for\ r}} = 0,1,2, \ldots ,{\mathrm{R}} - 1. \end{eqnarray*}

The Dense1 layer can generate an R-dimensional vector ${{\boldsymbol{y}}}^{{\mathrm{D1}}}$ while a Conv1D layer extracts features into different feature maps, and we used a SoftMax function as an activation function. The final layer is the Dense2 layer, which outputs only a $1 \times 2$ dimension array to represent the likelihood of phages and eukaryotic viruses. The output score is calculated as follows:


(7) \begin{eqnarray*}
\stackrel{\wedge}y_i^{D2} = {\mathrm{softmax}}\left( {{y}_i} \right) = \frac{{{e}^{{y}_i}}}{{\sum _{j = 1}^n{e}^{{y}_j}}}. \end{eqnarray*}

Moreover, the loss function is defined below:


(8) \begin{eqnarray*}
\textit{Loss} = - \sum _{i = 1}^Cy_i^{D2} \cdot \log \stackrel{\wedge}y_i^{D2}. \end{eqnarray*}

We employed the Adam optimizer (learning rate = 0.0005) and batch size 16 to train the neural network and update network weights (F = 128, S1 = S2 = 2, *P* = 0.32, and R = 64). The architecture of the IPEV neural network is depicted in Fig. 1C. When using IPEV, the final viral taxon scores are obtained by weighted averaging of the subsequence predictions. The detailed calculation methodology is outlined in the Supplementary Materials and Methods section.

## Results

### Performance on viral genome fragments using cross-validation

**Figure 2:**
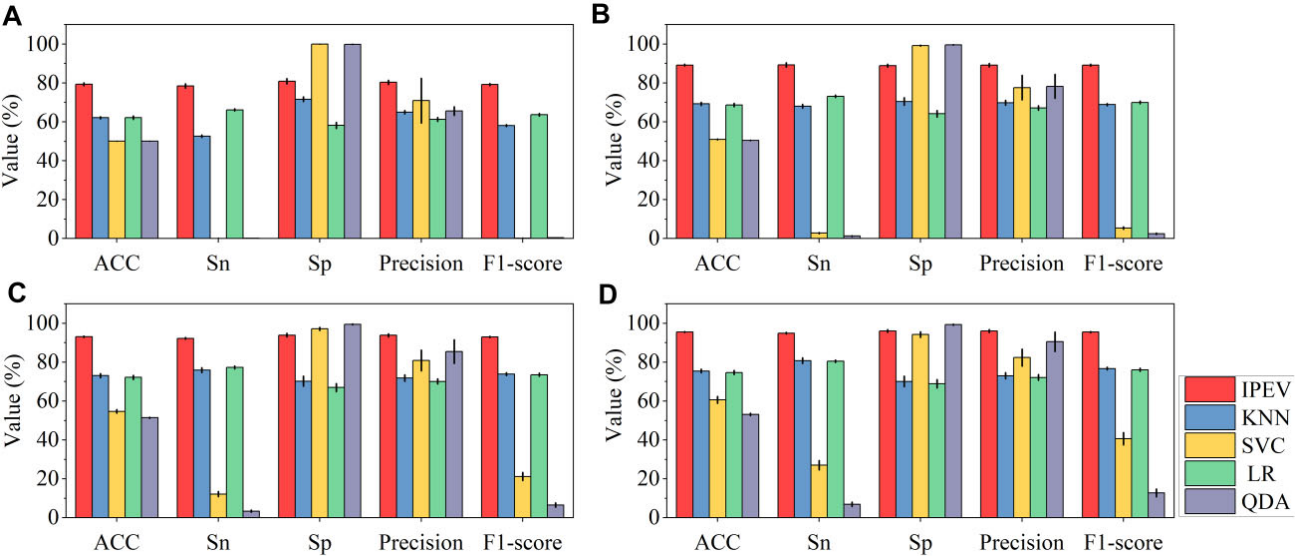
Panels A, B, C, and D display the comparative performance of IPEV and HTP (KNN, SVC, LR, and QDA) with 5-fold cross-validation across groups A, B, C, and D, respectively. Sn = TP/(TP + FN), Sp = TN/(TN + FP), ACC = (TP + TN)/(TP + TN + FP + FN), Precision = TP/(TP + FP), F1-score = 2 × Precision × Recall/(Precision + Recall), where TP, TN, FP, and FN respectively represent true positive, true negative, false positive, and false negative. As the method with the best performance in HTP, KNN is selected for comparison. The mean and standard deviation of 5-fold cross-validation are computed to elaborate on performance evaluation. Due to a lack of reconstruction between the train and validation sets, the performance of HTP (KNN) is overestimated. (In this article, prokaryotic viruses are treated as positive samples.)

**Figure 3:**
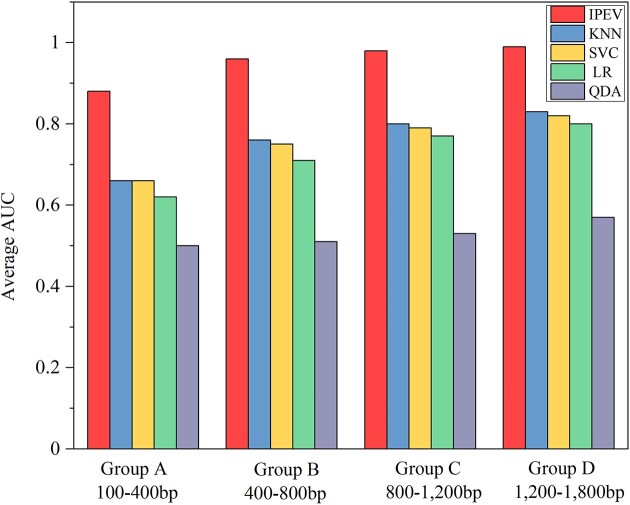
The average performance of IPEV and HTP (KNN) with 5-fold cross-validation for various sequence lengths, expressed as a percentage.

To evaluate the performance of IPEV, we implemented a 5-fold cross-validation procedure on groups A to D. The HTP tool, which we compared, comprises 4 distinct classifiers: KNN, SVC, LR, and QDA. The results showed that IPEV performed better than KNN, SVC, LR, and QDA by an average area under the curve (AUC) value increase of 0.16, 0.18, 0.20, and 0.22, respectively, in group D (1,200–1,800 bp). This is further depicted in Figs. 2 and 3 and Supplementary Table S4. Additionally, we observed that the performance of the model’s predictions is directly proportional to the nucleotide sequence length. The AUC value of IPEV increased from 0.88 to 0.99, from group A (100–400 bp) to group D (1,200–1,800 bp). In contrast, the AUC value of HTP (KNN) increased from 0.66 to 0.83, as shown in Supplementary Fig. S1.

In binary classification, sensitivity (Sn) and specificity (Sp) evaluate the ability to predict positive and negative samples. As shown in Fig. 2 and Supplementary Table S3, in group A (100–400 bp), the performance of HTP (KNN) in accurately identifying prokaryotic viruses (considered as positive samples) was found to be modest, achieving an Sn of 52.6% and an Sp of 71.6%. In contrast, our IPEV tool demonstrated rather better performance under the same conditions, with an Sn of 78.4% and an Sp of 80.9%. This suggests that IPEV outperforms HTP (KNN) in accurately classifying short viral fragments. This implies that for some species with low abundance or insufficient sequencing depth, it may not be possible to assemble longer phage sequences, and HTP may fail to identify them. IPEV, on the other hand, does not exhibit a specific preference. In group D (1,200–1,800 bp), the performance of HTP (KNN) in accurately identifying eukaryotic viruses was found to be modest, with an Sn of 80.8% and an Sp of 70.1%. Conversely, under the same conditions, the IPEV tool exhibited superior performance, with an Sn of 95.0% and an Sp of 96.1%. In addition, we plotted the training loss, validation loss, training accuracy, and validation accuracy curves with respect to the number of epochs using 5-fold cross-validation. We observed that the model converged at 30 epochs, with training and validation losses and accuracies remaining consistent and overlapping. This indicates that the model achieved high performance while avoiding overfitting or underfitting the data, as shown in Supplementary Figs. S8 and S9. We also tested IPEV’s effectiveness in differentiating viral from nonviral genome fragments in datasets where viruses and nonviruses are present in a 50:50 ratio. As shown in Supplementary Fig. S12, across groups A to D, IPEV achieved Sn scores of 0.73, 0.833, 0.905, and 0.931, respectively (details on sample construction and methods can be found in Supplementary Materials and Methods).

### Performance on novel viruses with low homology to known databases

Tools designed for predicting viral taxa aim to accurately identify newly discovered viruses, especially those exhibiting low homology to existing viral databases [40, 53, 54]. However, evaluating the performance of such tools is challenging owing to the lack of accurate labels for new viral sequences. This study defines a novel virus as one with very low homology to known viruses. To evaluate IPEV’s effectiveness, we constructed several independent test sets in which sequence identity increased compared to the training set of IPEV; these evaluations are designed to measure IPEV’s performance on “unseen” viruses. These 6 low homology test sets (datasets I1–I6) were generated with varying degrees of query coverage and identity relative to the training set. Related details are shown in the Supplementary Materials and Methods. For dataset I1, the majority of high homology sequences were eliminated, applying a threshold of 30% identity and 30% coverage. This highly labeled and unbalanced dataset I1 comprises groups A, B, C, and D, containing 3,180, 3,152, 3,050, and 3,171 prokaryotic virus contigs, respectively, and 1,106, 1,117, 1,011, and 1,036 eukaryotic virus contigs, respectively.

**Figure 4:**
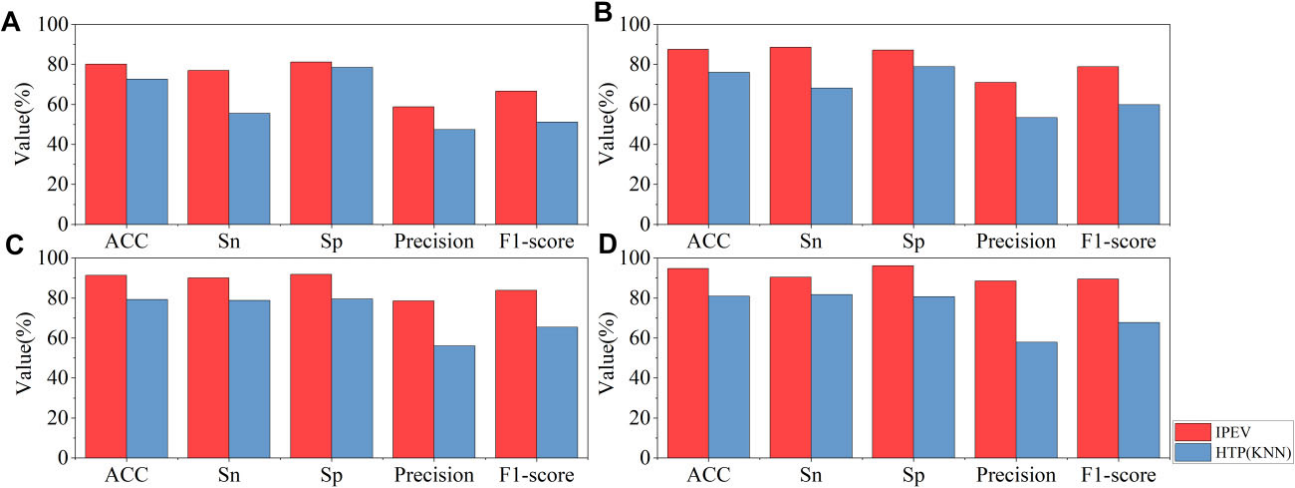
Panels A, B, C, and D display the comparative performance of IPEV and HTP (KNN) across groups A, B, C, and D, respectively, of dataset I1 (parameter: query coverage = 30%, identity = 30%).

**Figure 5:**
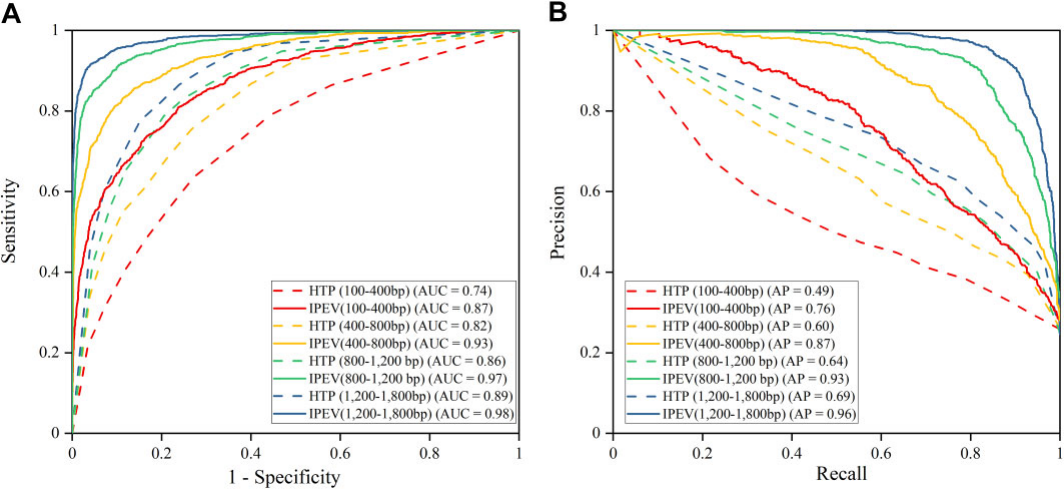
Performance comparison between IPEV and HTP (KNN) on dataset I1. (A) The receiver operating characteristic (ROC) curve demonstrates the discrimination capability, particularly in class-balanced test sets, with higher AUC values preferred. (B) The precision-recall curves measure discrimination capability in class-imbalanced test sets, with AP representing the average precision.

Despite removing sequences with high homology from the training set, IPEV still outperformed HTP, as shown in Fig. 4 and Supplementary Table S7. Specifically, in group A (100–400 bp) of dataset I1, IPEV reported an Sn of 76.94% and an Sp of 81.19%. On the other hand, HTP (KNN) reported an Sn of 55.52% and an Sp of 78.55%. In group D (1,200–1,800 bp), IPEV outperformed HTP (KNN) in Sn and Sp by 10% and 16%, respectively. These results underscore the superior performance of IPEV in distinguishing prokaryotic viruses from eukaryotic viruses. Meanwhile, we observed that as the sequence length increased from group A (100–400 bp) to group D (1,200–1,800 bp), the F1-score of IPEV improved from 66.61% to 89.44%. In contrast, the F1-score for HTP (KNN) increased from 51.12% to 67.81%. This performance is consistent with the results of a 5-fold cross-validation. As shown in Figs. 4 and 5, in terms of the AUC metric, IPEV outperformed HTP (KNN) by 0.13, 0.11, 0.11, and 0.09 in groups A, B, C, and D, respectively. These results demonstrate the advantage of IPEV in handling short fragments and datasets with low homology.

Furthermore, to evaluate the performance of IPEV, we utilized datasets I2 to I6, each exhibiting different levels of homology to the training set. As the number of high homology sequences between the independent test set and the training set increases, we observe a gradual improvement in the performance of IPEV, as shown in Supplementary Tables S7–S12 and SupplementaryFigs. S3–S7. The results indicate that the similarity between the test and training sets is crucial to determining the classification performance. Supplementary Table S12 demonstrates that in group D (1,200–1,800 bp) of dataset I6 (query parameter: coverage = 100% identity = 60%), the F1-score that can be achieved with IPEV is 96%. In contrast, the highest F1-score reported with HTP (KNN) is 77%.

On the other hand, it is also important to consider the impact of label imbalances on model performance. In datasets I1 to I6, which vary in their similarity to the training set of IPEV and in their respective length ranges (from group A to group D), IPEV’s Sn and Sp did not show significant variation as the label imbalance increased, while HTP (KNN) exhibited obvious changes. This indicates that IPEV maintains robust predictive performance, even when tested on sets with low sequence homology to the training set, and does not exhibit a bias in binary classification.

### Performance on test sets with sequencing errors

In this subsection, we evaluate the performance of IPEV and HTP on a dataset with varying levels of sequencing errors, including base insertion, deletion, and substitution. Errors in NGS are determined by the sequencing method and the experimental environment. Specifically, NGS has an error rate of 0.06% to 0.24% per base, while third-generation sequencing, such as PacBio, even exhibits a higher error rate of 5% to 15% per base [55, 56]. To evaluate the robustness of IPEV, we employed MetaSim to generate 2,000 short reads for both eukaryotic and prokaryotic viruses with different sequencing errors ranging from 1,200 to 1,800 bp. As shown in Table 1, the AUC values for both IPEV and HTP decrease with an increasing proportion of sequencing errors. When the error rate (base substitutions) reaches 15%, IPEV outperforms HTP (KNN) by approximately 16% in terms of the AUC metric. When the error rate (base insertions or deletions) reaches 15%, IPEV outperforms HTP (KNN) by approximately 17% in terms of the AUC metric. The observed phenomenon may be attributed to the sequence pattern matrix’s capacity to tolerate errors. Notably, our results indicate that the performance of IPEV is minimally affected by the percentage of sequencing errors introduced. Specifically, when the substitution-induced error rate rises from 0% to 15%, the AUC value declines slightly from 0.99 to 0.91. These findings underscore the robustness and reliability of IPEV, showing its ability to maintain its performance despite the presence of sequencing errors.

**Table 1:** Comparison of IPEV and HTP’s AUC value on artificial datasets (1,200–1,800 bp) with varying error rates

Error rate (%)	Base substitutions		Base insertions or deletions	
	IPEV	HTP	IPEV	HTP
0	0.99	0.82	0.99	0.82
5	0.98	0.80	0.98	0.80
10	0.95	0.77	0.97	0.80
15	0.91	0.75	0.95	0.78

### Performance on functional protein sequences

In addition to evaluating short sequence fragments, we comprehensively evaluated the capability of the IPEV tool by incorporating protein sequences with functional annotations. One key aspect of understanding virus classification is identifying critical markers that play a role in the classification process, even though deep learning is often considered a black box.

**Figure 6:**
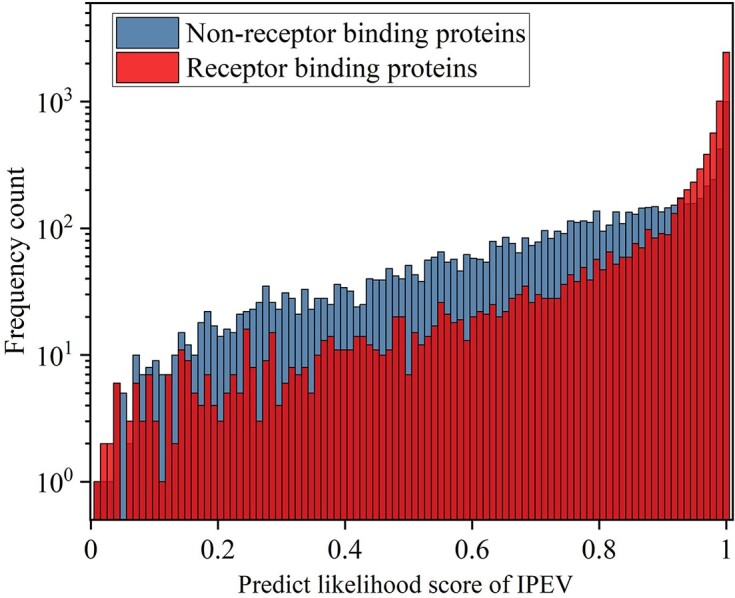
Histogram illustrating the predicted likelihood scores generated by IPEV for RBPs and non-RBPs.

Considering the crucial role of RBPs in the adsorption and host invasion of bacteriophages, we formulated a hypothesis suggesting their substantial contribution to the accuracy of phage prediction. To test this hypothesis, we carefully assembled a dataset of 7,384 RBPs, complemented with corresponding negative samples, verified manually. We aimed to evaluate the impact of RBPs on phage prediction accuracy. The IPEV prediction results showed that RBPs significantly contributed to phage prediction accuracy. The predicted likelihood score by IPEV had a mean of 0.90 and a median of 0.98 for the RBP set, while for the non-RBP set, the mean was 0.77 and the median was 0.83, as shown in Fig. 6 (Wilcoxon rank-sum test, *P* < 2.2e-16). The findings from our study also indicate that our model possesses the capability to learn host-related information to a certain degree. This emphasizes the significance of integrating host-related data into phage prediction models. To assess our model’s performance in predicting eukaryotic viruses, we conducted a focused analysis of 7 experimentally confirmed capping enzymes. The results revealed that our model accurately predicted the likelihood of these protein sequences, achieving a score close to 1. This result provides compelling evidence of the model’s high accuracy and effectiveness in predicting eukaryotic viruses. For additional details regarding these results, please refer to Supplementary Table S14.

### Performance on the marine virome

**Figure 7:**
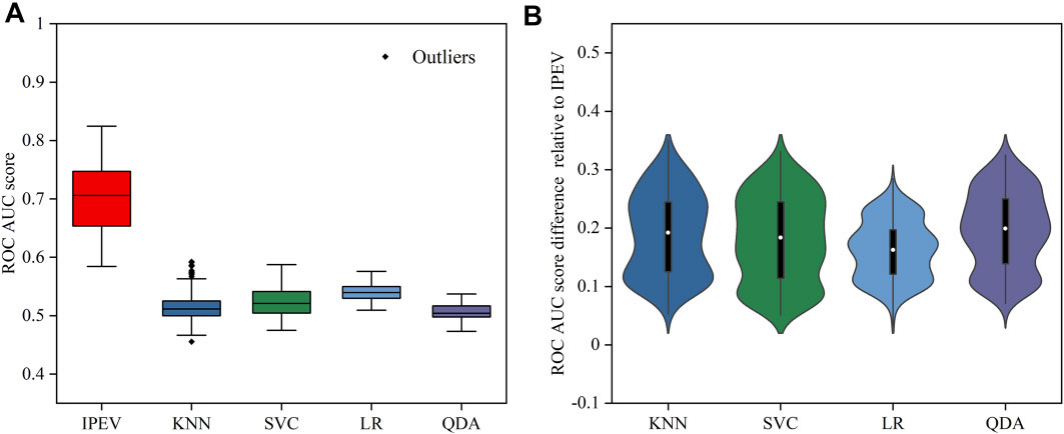
(A) Box plots representing AUC scores of the ROC curves for IPEV, KNN, SVC, LR, and QDA. (B) Violin plots displaying the AUC score differences of each tool relative to IPEV.

We collected data from 243 marine virome samples with assembled contigs and annotated them for sequence type using BLASTn. We evaluated our tool, IPEV, along with other related tools. We reported the overall average and median AUC for the samples, and we noted that our tool, IPEV, outperforms HTP (KNN, and others) across all samples. As shown in Fig. 7, in our comprehensive evaluation, IPEV demonstrated its advanced capabilities by consistently outperforming other tools with its higher average AUC values: it outperforms SVC by 0.18, exceeds QDA by 0.20, surpasses LR by 0.16, and betters KNN by 0.19. These results indicate that IPEV is a highly competitive tool.

### Applying IPEV to analyze the longitudinal gut virome in a cohort study

Within this subsection, we provide a comprehensive overview of our analysis encompassing 2 primary aspects. First, we assess the accuracy of IPEV, along with its related tool, in analyzing the gut virome. Second, we explore the temporal stability of the healthy gut virome utilizing longitudinal data spanning a 1-year period, employing the IPEV tool.

**Figure 8:**
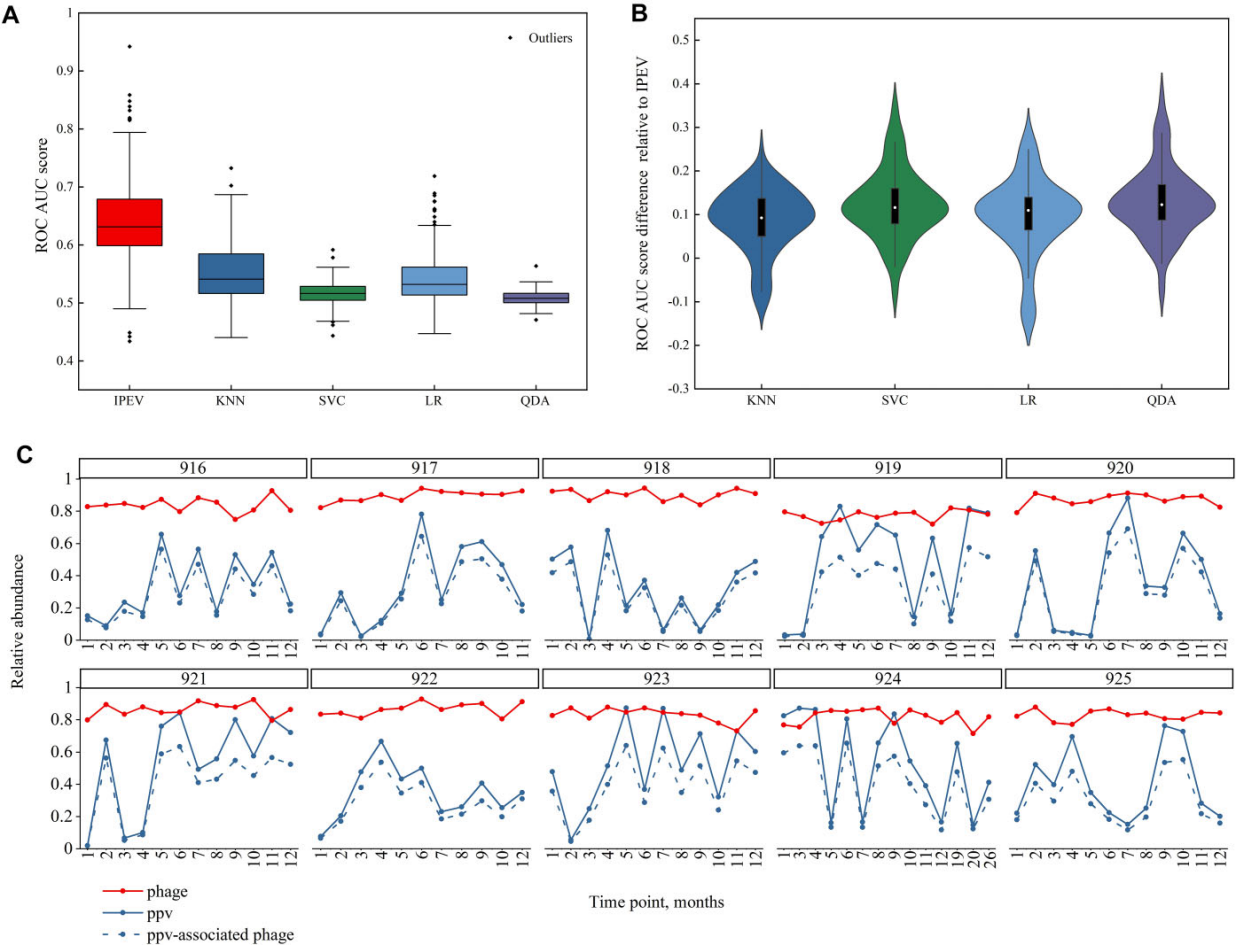
(A) Box plots representing the AUC scores of ROC curves for IPEV, KNN, SVC, LR, and QDA. (B) Violin plots displaying the AUC scores differences of each tool relative to IPEV. (C) Relative abundances of phages, PPV, and PPV-associated phages in the longitudinal data of subjects 916 to 925 as determined by IPEV (details of the annotations can be found in the Supplementary Materials and Methods).

In our study, we analyzed a dataset comprising 130 samples collected from 10 subjects, which were originally obtained in Shkoporov et al.’s [50] study. We processed and annotated the raw data following the methodology outlined in the Materials and Methods section. We utilized the IPEV tool and its associated software on each distinct sample to generate predictions and compute AUC scores, leveraging BLAST annotation in the process. Our analysis, illustrated in Fig. 8A, demonstrated that IPEV exhibited higher accuracy than HTP in over 90% of the real virus samples. Furthermore, IPEV’s mean AUC value of 0.64 was significantly superior to those of KNN (0.55), SVC (0.51), LR (0.54), and QDA (0.51), according to the Wilcoxon rank-sum test results using Benjamini–Hochberg adjustment (IPEV with KNN: *P*_adj_ < 2.89e-19; IPEV with SVC: *P*_adj_ < 1.02e-33; IPEV with LR: *P*_adj_ < 6.97e-22; IPEV with QDA: *P*_adj_ < 1.52e-36).

Additionally, the median AUC for IPEV was 0.63, significantly higher than that of 0.54, 0.52, 0.53, and 0.51 for KNN, SVC, LR, and QDA, respectively. The median difference between the AUC of IPEV and KNN, SVC, LR, and QDA for each sample is shown in Fig. 8B, with median differences of 0.09, 0.12, 0.11, and 0.12, respectively. We observed that the performance of QDA is equivalent to random guessing, with median 0.51 AUC scores. While the performance of IPEV on the simulated dataset was not as remarkable as that on the real virome data, this disparity can be attributed to a notable factor. The assembled sequences in our dataset of 130 samples predominantly comprised short sequences, with those below 500 bp representing a significant portion of 80% of the total. Coping with this challenge, our model persevered and successfully identified viruses within the real virome, demonstrating its effectiveness and robustness.

The human gut virome is characterized by its immense diversity and abundance of virus particles. The gut feces contain up to approximately 10^9^ VLPs per gram, yet only a fraction of the virus genomes, ranging from 14.2% to 56.6%, can be annotated [57]. Previous research [50] has identified a highly individualized and persistent fraction in the gut virome, the PPV. Additionally, this research has observed temporal stability in the virus components at the individual level. To further explore the virome, we employed IPEV to *ab initio* annotate contigs in the virome while excluding bacterial contamination. The results showed that the average coefficient of variation of the phageome was significantly lower (0.04 ± 0.01) compared to the PPV (0.58 ± 0.05), indicating a high degree of temporal stability of the phageome, as illustrated in Fig. 8C and Supplementary Table S15. Our findings align with the studies of Shkoporov et al. [50] and offer further substantiation, this time at a higher taxonomic level, for the hypothesis that the “kill-the-winner” mechanism operates effectively in phage strains and substrains. This mechanism prevents the dominance of any single species, facilitating the coexistence of multiple species instead. Consequently, it enhances the diversity of the gut ecosystem and reinforces its overall resilience [58]. In contrast, the PPV is dominated by lytic life cycles and composed of virulent *CrAss-like* and *Microviridae phages* [50] that infect major representatives of the bacterial microbiota, resulting in higher variations than those seen in gut ecology.

We also observed limited transient disturbance in the phageome component in subjects 916 (T1 and T7) and 922 (T3, T5, and T8) with antibiotic usage. An intriguing observation emerged from our study: despite variations in individual characteristics such as gender, body mass index, and lifestyle factors like smoking and alcohol consumption, the relative abundances of the phageome remained remarkably similar among individuals. In contrast, the relative abundances of the PPV displayed substantial individual variations. To delve deeper into these findings, we employed IPEV to assess the contribution of phage and eukaryotic viromes to the variability of PPV. Notably, our analysis revealed that phages constituted the primary component and exerted significant dominance over fluctuations in PPV, as shown in Fig. 8C. This highlights the crucial role of phages in driving the variability of PPV. By elucidating these relationships, our results contribute to a better understanding of the overall stability and dynamics of the gut virome.

## Discussion

This study introduces IPEV, a novel method that utilizes a sequence pattern matrix and a 2D CNN to distinguish prokaryotic and eukaryotic virus-derived sequence fragments. To the authors’ best knowledge, IPEV is the first *de novo* identification algorithm tool developed to address this type of problem for virome data.

IPEV offers several advantages over traditional genomics techniques, such as *k*-mer methods and one-hot encoding. By integrating the position and frequency information of 3-mers into a sequence pattern matrix, IPEV enhances the efficiency of the neural network model and preserves valuable information about the order and position of trinucleotides.

To ensure the generalization evaluation of the presented tool, we opted not to use the traditional approach of dividing the dataset into training and test sets based on the date the sequence was discovered [37, 59]. This method can include sequences with high homology to the training set in the test set, inflating the algorithm’s accuracy and making it challenging to assess its performance accurately. Instead, we used a series of thresholds to gradually remove sequences from the test set that were homologous to the training set. For artificial contigs with less than 30% sequence homology against the training set, IPEV achieved an average AUC of 0.98, indicating that our model learned valuable information and did not rely solely on sequence similarity for prediction. Our results suggest that IPEV can generalize well and provide reliable predictions for prokaryotic and eukaryotic virus-derived sequence fragments.

Our objective was to analyze the neural network by examining its probability scores for phage prediction. The presence of the RBP is crucial for the specific interaction between the bacteriophage and its host. Therefore, we specifically focused on genes with manual confirmation that are associated with RBPs, as well as an equal number of non-RBP genes. Upon comparing the score distribution of IPEV between the 2 datasets, we observed a notable difference. The former dataset exhibited a higher score distribution in IPEV. This discrepancy suggests that RBPs with host information could substantially influence phage prediction within the IPEV framework. As the neural network model learns from well-annotated data, IPEV can also uncover valuable insights regarding the virus–host relationship. This contributes to our overall understanding of neural networks and their performance in relevant tasks.

We utilized the IPEV approach to conduct a comprehensive analysis of longitudinal data from the gut virome, which includes eukaryotic viruses primarily acquired from the environment and diet as well as prokaryotic viruses that target microorganisms [50]. Our investigation revealed that the phage community exhibits remarkable resilience to environmental disturbances, with the “kill the winner” theory having a minimal impact at a higher taxonomic level. This finding aligns with previous research and supports the hypothesis that the “kill-the-winner” theory fosters a more diverse species community, ultimately enhancing ecosystem resilience. Furthermore, we observed significant fluctuations in the abundance of PPV, primarily driven by phages that specifically target representative gut microbiota.

Although IPEV was initially designed for identifying short viral sequences, our study also encompassed an evaluation over extended lengths of 3,000 to 5,000 bp. This length spectrum allowed us to include vConTACT v.2.0 and iPHoP in our assessment. We constructed 3 datasets with contig lengths of 3,000–5,000 bp (details on dataset construction and evaluation methods are provided in the Supplementary Materials and Methods). Our findings indicate that IPEV consistently outperformed the others, achieving an average F1-score of 0.99, as depicted in Supplementary Fig. S11. This score significantly exceeds the F1-scores of 0.02 for vConTACT v.2.0 (*t* = −31.29, *P*_adj_ < 0.0001, 2-tailed independent *t*-tests using Benjamini–Hochberg adjustment) and 0.41 for iPHoP (*t* = −395.63, *P*_adj_ < 0.0001, 2-tailed independent *t*-tests), respectively.

Moreover, to fulfill the demands of high-throughput data processing, we implemented a design for IPEV that enhances the efficiency of analyzing large datasets. IPEV optimizes processing time by returning all taxon prediction scores in a single iteration, requiring only 4 loads of the neural network weight. Unlike HTP, which expends processing time on file input/output operations, this approach allows IPEV to handle large datasets more effectively. This substantial reduction in computational overhead significantly improves the running speed of IPEV. As shown in Supplementary Fig. S2, IPEV operates 50 times faster than HTP, 30 times faster than vConTACT v.2.0, and 1,225 times faster than iPHoP, taking only 9.6 minutes to analyze 20,000 sequences of 1,200 to 1,800 bp when using the same computational resources (CPU: Intel^®^ Xeon^®^ 20 cores, GPU: NVIDIA Tesla V100).

An important consideration when using IPEV is the potential bacterial and fungal contamination in virome datasets [60, 61]. To address this, we designed a feature within IPEV that eliminates false-positive nonviral components (bacteria and fungi). This feature is available as an optional switch. Our current tool has limitations as it does not encompass downstream virus classification or provide specific virus–host predictions for virome data. A more precise and systematic classification system is required to understand the influence viruses have on microbial communities and hosts. We eagerly anticipate the development of additional tools that will reduce the extent of virome dark matter and enhance our comprehension of the virome’s intricacies.

## Availability of Supporting Source Code and Requirements

Project name: IPEV

Project homepage: https://github.com/basehc/IPEV or https://cqb.pku.edu.cn/zhulab/info/1006/1156.htm (including code and a detailed tutorial)

Operating system: IPEV is platform independent.

Programming language: Python

Other requirements: IPEV is built on Python 3.8.6 and Tensorflow 2.3.1 (RRID:SCR_016345).

License: GNU GPL v3

RRID:SCR_023702

BioTools ID: IPEV

## Supplementary Material

giae018_GIGA-D-23-00357_Original_Submission

giae018_GIGA-D-23-00357_Revision_1

giae018_GIGA-D-23-00357_Revision_2

giae018_Response_to_Reviewer_Comments_Original_Submission

giae018_Response_to_Reviewer_Comments_Revision_1

giae018_Reviewer_1_Report_Original_Submission Guillermo Andres Rangel-Pineros -- 1/22/2024 Reviewed

giae018_Supplemental_File

## Data Availability

Our study contains only publicly available viral genome sequences and reference bacterial genome sequences (ENA study accession: PRJEB22493, NCBI BioProject accession: PRJNA545408). An archival copy of the code is available via Software Heritage [62]. DOME-ML annotations are available via the DOME wizard [63]. Code and data for transparent and reproducible results are documented in Zenodo [64] and a Docker image [65].

## Additional Files

**Supplementary Fig. S1**. The ROC curves and AUC value of IPEV and HTP performances in each set of 5-fold cross-validation.

**Supplementary Fig. S2**. Performance of IPEV, HTP, vConTACT2, and iPHoP as the number of sequences increases under the same computing configuration (1,200–1,800 bp).

**Supplementary Fig. S3–S7**. Comparison of IPEV and HTP on datasets I2 to I6.

**Supplementary Fig. S8**. Mean accuracy, mean loss, and mean of IPEV’s 5-fold cross-validation on group A.

**Supplementary Fig. S9**. The mean validation loss and accuracy of IPEV’s 5-fold cross-validation on group A.

**Supplementary Fig. S10**. (A) Performance (accuracy, specificity, sensitivity, precision, and F1-score) of IPEV and HTP (KNN, SVC, LR, QDA) on the duck gut virome data. (B). The ROC curve and AUC value of IPEV and HTP (KNN, SVC, LR, QDA) on the duck gut virome data.

**Supplementary Fig. S11**. Average performance of IPEV, HTP, iPHoP, and vConTACT v.2.0 across 3 independent test sets with sequence lengths of 3,000 to 5,000 bp.

**Supplementary Fig. S12**. The confusion matrices show the false-positive reduction capability of IPEV on datasets with a 1:1 ratio of viruses to nonviruses across groups A to D.

**Supplementary Table S1**. Details the distribution of contigs across various length ranges within a 5-fold cross-validation dataset based on Virus–Host DB.

**Supplementary Table S2**. Number of simulated contigs based on RVDB and IMG/VR v4 databases.

**Supplementary Table S3**. The average performance of IPEV and HTP (KNN) with 5-fold cross-validation for various sequence lengths, expressed as a percentage.

**Supplementary Table S4**. The average AUC value for 4 length groups on a 5-fold cross-validation.

**Supplementary Table S5**. Enumerates the numbers of the prokaryotic virus contigs in datasets I1 to I6.

**Supplementary Table S6**. Enumerates the numbers of the eukaryotic virus contigs in datasets I1 to I6.

**Supplementary Table S7–S12**. Compare the performance of IPEV and HTP under various query parameters for coverage and identity in datasets I1 to I6.

**Supplementary Table S13**. Performance of IPEV and HTP on the ducks’ gut virome data a total of 682 eukaryotic contigs and 1453 prokaryotic virus contigs.

**Supplementary Table S14**. Likelihood scores predicted by IPEV algorithm on capping enzymes.

**Supplementary Table S15**. *P* values show the phageome’s average coefficient of variation was significantly lower compared to the PPV (Wilcoxon rank-sum test, adjusted using the Benjamini–Hochberg correction).

## Abbreviations

2D: 2-dimensional; AUC: area under the curve; CNN: convolutional neural network; ENA: European Nucleotide Archive; HTP: Host Taxon Predictor; IPEV: Identify Prokaryotic and Eukaryotic Virus-derived sequences; NCBI: National Center for Biotechnology Information; NGS: next-generation sequencing; PPV: personal persist virome; RBP: receptor-binding protein; ReLU: Rectified Linear Unit; ROC: receiver operating characteristic; RVDB: Reference Viral Database; SGT: Sequence Graph Transform; Sn: sensitivity; Sp: specificity; VLP: virus-like particle.
