# Supplementary material for: IPEV: identification of prokaryotic and eukaryotic virus-derived sequences in virome using deep learning
Source: Gigascience. 2024 Apr 22;13:giae018. doi: 10.1093/gigascience/giae018 (PMC11034026; doi:10.1093/gigascience/giae018)
Supplement: giae018_GIGA-D-23-00357_Original_Submission [file giae018_giga-d-23-00357_original_submission.pdf]

GigaScience

IPEV: identification of prokaryotic and eukaryotic virus-derived sequences in virome using deep learning

--Manuscript Draft--

Manuscript Number:	GIGA-D-23-00357
Full Title:	IPEV: identification of prokaryotic and eukaryotic virus-derived sequences in virome using deep learning
Article Type:	Technical Note
Funding Information:	National Natural Science Foundation of China (32070667) — Dr Huaiqiu Zhu<br>National Natural Science Foundation of China (31671366) — Dr Huaiqiu Zhu<br>National Natural Science Foundation of China (32300078) — Dr Xiaoqing Jiang<br>National Key Research and Development Program of China (2021YFC2300300) — Dr Huaiqiu Zhu
Abstract:	Background: The virome obtained through virus-like particle enrichment contains a mixture of prokaryotic and eukaryotic virus-derived fragments. Accurate identification and classification of these elements are crucial to understanding their roles and functions in microbial communities. However, the rapid mutation rates of viral genomes pose challenges in developing high-performance tools for classification, potentially limiting downstream analyses.<br>Findings: We present IPEV, a novel method to distinguish prokaryotic and eukaryotic viruses in viromes, with a 2D convolutional neural network combining trinucleotide pair relative distance and frequency. Cross-validation assessments of IPEV demonstrate its state-of-the-art precision, significantly improving the F1-score by approximately 22% on an independent test set compared to existing methods when query viruses share less than 30% sequence similarity with known viruses. Furthermore, IPEV outperforms other methods in accuracy on marine and gut virome samples based on annotations by sequence alignments. IPEV reduces runtime by at most 1,225 times compared to existing methods under the same computing configuration. We also utilized IPEV to analyze longitudinal samples and found that the gut virome exhibits a higher degree of temporal stability than previously observed in persistent personal viromes, providing novel insights into the resilience of the gut virome in individuals.<br>Conclusions: IPEV is a high-performance, user-friendly tool that assists biologists in identifying and classifying prokaryotic and eukaryotic viruses within viromes. The tool is available at https://github.com/basehc/IPEV.
Corresponding Author:	Huaiqiu Zhu<br><br>CHINA
Corresponding Author Secondary Information:	
Corresponding Author's Institution:	
Corresponding Author's Secondary Institution:	
First Author:	Hengchuang Yin
First Author Secondary Information:	
Order of Authors:	Hengchuang Yin<br>Shufang Wu<br>Jie Tan

Powered by Editorial Manager® and ProduXion Manager® from Aries Systems Corporation

	Qian Guo
	Mo Li
	Jinyuan Guo
	Yaqi Wang
	Xiaoqing Jiang
	Huaiqiu Zhu
**Order of Authors Secondary Information:**	
**Additional Information:**	
**Question**	**Response**
Are you submitting this manuscript to a special series or article collection?	No
**Experimental design and statistics**<br><br>Full details of the experimental design and statistical methods used should be given in the Methods section, as detailed in our Minimum Standards Reporting Checklist. Information essential to interpreting the data presented should be made available in the figure legends.<br><br>Have you included all the information requested in your manuscript?	Yes
**Resources**<br><br>A description of all resources used, including antibodies, cell lines, animals and software tools, with enough information to allow them to be uniquely identified, should be included in the Methods section. Authors are strongly encouraged to cite Research Resource Identifiers (RRIDs) for antibodies, model organisms and tools, where possible.<br><br>Have you included the information requested as detailed in our Minimum Standards Reporting Checklist?	Yes
**Availability of data and materials**<br><br>All datasets and code on which the	Yes

conclusions of the paper rely must be either included in your submission or deposited in publicly available repositories (where available and ethically appropriate), referencing such data using a unique identifier in the references and in the “Availability of Data and Materials” section of your manuscript.<br><br>Have you have met the above requirement as detailed in our Minimum Standards Reporting Checklist?	

Powered by Editorial Manager® and ProduXion Manager® from Aries Systems Corporation

Manuscript
Click here to access/download;Manuscript;IPEV_R1_revised.docx

IPEV: identification of prokaryotic and eukaryotic virus-derived sequences in virome using deep learning

Hengchuang Yin[1], Shufang Wu[1], Jie Tan[1], Qian Guo[1], Mo Li[1,2], Jinyuan Guo[1,3], Yaqi Wang[1], Xiaoqing Jiang[1,4] and Huaiqiu Zhu[1,2,3]*

[1] Department of Biomedical Engineering, College of Future Technology, and Center for Quantitative Biology, Peking University, Beijing 100871, Beijing, China;

[2] School of Life Sciences, Peking University, Beijing 100871, Beijing, China;

[3] Department of Biomedical Engineering, Georgia Institute of Technology and Emory University, GA 30332, Atlanta, USA

[4] Beijing Institute of Genomics, Chinese Academy of Sciences, and China National Center for Bioinformation, Beijing 100101, China

* To whom correspondence should be addressed. Tel: 8610-6276 7261; Email: hqzhu@pku.edu.cn

Abstract

Background: The virome obtained through virus-like particle enrichment contains a mixture of prokaryotic and eukaryotic virus-derived fragments. Accurate identification and classification of these elements are crucial to understanding their roles and functions in microbial communities. However, the rapid mutation rates of viral genomes pose challenges in developing high-performance tools for classification, potentially limiting downstream analyses.

Findings: We present IPEV, a novel method to distinguish prokaryotic and eukaryotic viruses in viromes, with a 2D convolutional neural network combining trinucleotide pair relative distance and frequency. Cross-validation assessments of IPEV demonstrate its state-of-the-art precision, significantly improving the F1-score by approximately 22% on an independent test set compared to existing methods when query viruses share less than 30% sequence similarity with known viruses. Furthermore, IPEV outperforms other methods in accuracy on marine and gut virome samples based on annotations by sequence alignments. IPEV reduces runtime by at most 1,225 times compared to existing methods under the same computing configuration. We also utilized IPEV to analyze longitudinal samples and found that the gut virome exhibits a higher degree of temporal stability than previously observed in persistent personal viromes, providing novel insights into the resilience of the gut virome in

individuals. **Conclusions:** IPEV is a high-performance, user-friendly tool that assists biologists in identifying and classifying prokaryotic and eukaryotic viruses within viromes. The tool is available at https://github.com/basehc/IPEV.

INTRODUCTION

Viruses and virus-like particles (VLPs) are abundant and diverse biological entities on Earth. It is estimated that there are approximately 10^{31} viral particles [1], showcasing their pervasive nature. Notably, even in human feces, there can be as many as 10^{9} VLPs per gram, further emphasizing their prevalence and ubiquity [2, 3]. The advent of next-generation sequencing (NGS) technology has revolutionized virome studies, enabling the discovery of novel viruses and significantly advancing our understanding of their potential influence on both environmental and human body microbiomes [4-7]. Nonetheless, it is essential to note that enriched sample approaches carry the risk of losing valuable host or environmental information [8] and can cause inaccurate virus host identification, thereby constraining subsequent analyses. A noteworthy example is our previous study, in which we found a strong correlation between alterations in the proportion of temperate phages within the gut virome and the occurrence of ulcerative colitis in patients [9]. Similarly, the significance of the eukaryotic virome should not be overlooked when examining the virome, as it is believed to play a vital role in both host health and disease [10-14]. Recent studies have shed light on intricate trans-kingdom interactions involving eukaryotic viruses, bacteria, and the host within the intestinal ecosystem [15-18]. Obviously, these analyses first require the precise differentiation of eukaryotic viruses from prokaryotic viral sequences, and it's beneficial for gaining a comprehensive understanding of the viral landscape [9, 15].

However, the precise identification of prokaryotic and eukaryotic viruses poses a significant challenge owing to the highly diverse and fast-mutating genetic elements within the virome. Furthermore, the limitations of assembly tools arise from factors such as mutations, recombination events, and often low or uneven sequencing coverage across the viral genome [19]. The abundance of short and frequently inadequate reads further complicates the task of virus identification. Moreover, the absence of a well-conserved genetic marker such as the bacterial 16S rRNA gene presents a barrier in constructing a phylogenetic tree to effectively differentiate between eukaryotic and prokaryotic viruses [20]. Virus identification typically involves aligning sequences against known viruses in genomic repositories, such as the National Center for Biotechnology Information (NCBI) Taxonomy Databases

[21] and European Nucleotide Archive (ENA) [22]. Despite the rapid increase in viral sequences, the number of viral reference sequences in public reference databases is still limited, constraining the effectiveness of sequence-based alignment methods. For instance, it has been estimated that there are millions of viral species, but the International Committee on Taxonomy of Viruses (ICTV) has recognized only 11,273 species to date [23].

Currently, some computational tools for identifying viruses have been introduced in metagenomes, such as HoPhage [24], iPHoP [25, 26], WIsH [27], CHERRY [28], PHP [29], and VHM-Net [30], designed to assign the host for a given phage contig using sequence similarity search or ab initio identification. Some tools, like DeePhage [9], PHACTS [31], and PhagePred [32], have been developed to answer questions about the lifestyles of phages. Other tools, like PPR-Meta [33], DeepVirFinder [34], VIBRANT [35], vConTACT2 [36], VirSorter [37], and the most recent version, VirSorter2 [38], can be used to identify viruses from metagenomics data. While analyzing and classifying phages in virome data is a current focus, it is important to note that eukaryotic viruses also play a critical role in influencing host immunity and disease phenotypes by infecting host cells and interacting with the bacterial microbiome through trans-kingdom interactions. However, existing methods for analyzing virome data are limited in distinguishing eukaryotic viruses from viromes, assigning hosts, and classifying them accurately [39]. Some methods, such as the Host Taxon Predictor (HTP) [40], can be used to bridge the gap, differentiating between phages and eukaryotic viruses based on sequence information and nucleic acid type (such as DNA or RNA). Given that HTP's performance is highly dependent on the nucleic acid type used, and that some experimental protocols for virome research may result in mixed datasets containing both DNA and RNA sequences [41], it might be challenging accurately determine the origin of these sequences. This uncertainty could, in our observations, influence the performance of HTP in classifying viruses.

In this paper, we present the IPEV (Identify Prokaryotic and Eukaryotic Virus-derived sequences), a high-performance, user-friendly tool for differentiating prokaryotic and eukaryotic viruses from virome sequence fragments. To achieve high performance on short viral sequences, we developed a 2D convolutional neural network (CNN) based on a sequence pattern matrix using the Sequence Graph Transform (SGT) model. Cross-validation tests demonstrate that IPEV significantly outperforms related methods in terms of F1-score metrics by at most 21.1% while requiring only 1/50th the time of HTP in the same computing environment. We also designed various homology layouts for independent sets

based on known sequence data to assess IPEV's generalization capabilities. IPEV outperforms HTP (KNN) by approximately 22% in terms of the F1-score on an independent set with highly imbalanced labels when the sequence identity between the training and independent test sets is less than 30%. IPEV's evaluations of marine virome samples are better than HTP, with much more accurate results. We applied IPEV to analyse the longitudinal gut virome data from ten healthy individuals over 12 months and achieved the best performance in at least 90% of the samples compared to other methods. Our analysis revealed that the gut virome exhibits temporal stability beyond that observed in persistent personal viromes, thus enhancing our understanding of gut virome stability in individuals.

MATERIALS AND METHODS

Dataset construction

Without accurately host-annotated virome datasets that could serve as benchmarks, we generated simulated datasets based on well-annotated complete virus genomes. Firstly, we downloaded the taxonomy ID list of viruses and corresponding host lineages from Virus-Host DB [42] and genome sequences from the NCBI database [43] on Oct. 31, 2021. As a result, we established our first dataset, referred to as Dataset-1, which contains 11,022 eukaryotic virus genomes and 5,051 prokaryotic virus genomes (of which 113 are attributed to archaeal viruses). To enhance the model's generalizability, we incorporated additional data from the Reference Viral Database (RVDB) [44] and 25,644 eukaryotic sequences along with 5,598 prokaryotic sequences from IMG/VR v4 [45], collectively termed Dataset-2. Details regarding the data inclusion criteria are outlined in the Supplementary Materials and Methods. Dataset-1 is sourced from reference sequences and manually curated with credible host annotations, while Dataset-2 is not. Therefore, based on genomes, we used all viruses in Dataset-2 and 10,000 eukaryotic and 4,000 prokaryotic viruses in Dataset-1 randomly divided for 5-fold cross-validation, while the remaining subset served as an independent test set for assessing generalizability.

Considering the limitations of current mainstream sequencing technologies and the length constraints of assembled contigs, we simulated four contig length groups (A-D) using MetaSim (v0.9.1) [46] with "exact" pre-set and "Uniform" distribution types. The contig length groups were as follows: Group A (100-400 bp), Group B (400-800 bp), Group C (800-1,200 bp), and Group D (1,200-1,800 bp). The specific contig numbers can be found in Tables S1 and S2 in the Supplementary Materials. We evaluated IPEV's generalization ability using an independent test set of 1,022 eukaryotic and 1,051

prokaryotic virus sequences. Using MetaSim, we totally generated 20,000 contigs and ensured low similarity against the training set using BLASTn (v2.7.1), following the above length groups. We generated six low homology independent test sets (Dataset I1-I6) with varying query coverage and identity thresholds relative to the training set. The number of corresponding prokaryotic and eukaryotic virus contigs in each independent test set can be found in Table S5 and Table S6, respectively.

We evaluated the effect of sequencing errors on IPEV's performance, and we generated a total of 10,000 contigs (1,200 to 1,800 bp) with 5%, 10%, and 15% sequencing errors based on the independent test set using MetaSim (related details can be found in the Supplementary Materials and Methods). Furthermore, we assessed the capability of the IPEV tool by analyzing protein sequences with functional annotations. We constructed a dataset of 7,384 RBPs and corresponding negative samples, which were manually verified. Our selection criteria revolved around methods that are oriented towards function, GO annotation, or product description and that feature RBP-related keywords. These protein sequences originated from a wide range of prokaryotic viruses, spanning seven orders and 28 families, including *Tubulavirales* and *Timlovirales*. To evaluate our model's efficacy in predicting eukaryotic viruses, we also assembled a collection of seven experimentally confirmed capping enzymes [47].

We also use real virome to evaluate IPEV and related tools. We first downloaded a dataset comprising 243 marine virome samples from the ENA (accession number: PRJEB22493 [48, 49]). Besides, we analyzed longitudinal data from Shkoporov et al.'s [50] study to evaluate IPEV's accuracy and the stability of gut virome data. We retrieved the raw human gut virome dataset from the NCBI SRA (accession number: PRJNA545408). This dataset included 130 virome samples from ten healthy adults (subjects 916-925) collected over 12 months (T1-T12) through monthly synchronous samplings. We utilized the SPAdes (v3.13.0) [51] software to assemble short reads and conducted BLASTn searches against a bacterial database to eliminate bacterial contamination with an e-value of e-5, an identity of 50%, and query coverage of 90%. Our reference bacterial dataset comprised 20,003 complete prokaryotic genomes sourced from the NCBI RefSeq database, comprising 19,629 bacterial genomes and 374 archaeal genomes. Following Shkoporov's personal persist virome (PPV) definition, we used cd-hit-est (v.4.8.1) with parameters (c 0.8, aS 0.8, d 0, n 5) to cluster decontaminated contigs and defined clusters containing contigs from at least six months as PPV clusters. For subject 917, sampled for 11 months, we modified the PPV definition to include contigs appearing in at least five months. We aligned the assembled contigs with reference virus sequences using BLASTn to assign virus taxon

labels. The potential prokaryotic or eukaryotic viruses were inferred from viral contigs with an e-value less than the cutoff of 1e-4.

Mathematical model of DNA sequences

In this study, we developed a sequence pattern matrix using the Sequence Graph Transform (SGT) model to extract meaningful information based on the relative positions of trinucleotides [52]. The pattern matrix is a numerical representation of the frequency and order of trinucleotide pairs. We generated a trinucleotide set by combining three nucleotides to represent a DNA sequence (*S*) and calculated the weights of trinucleotides u and v using the following formula:

$$\psi_{uv}(s) = \frac{\sum_{\forall(l,m)} e^{-k|m-l|}}{|\Lambda_{uv}(s)|}. \quad (1)$$

Herein, $e^{-\kappa|m-l|}$ represent the weight of a trinucleotide pair of u and v at the position of m and l. The relative distances of a trinucleotide pair of u and v are measured by $|m-l|$. $\left|\Lambda_{uv}\right|$ is the size of the set Λ_{uv}. It represents the size of total (u, v) pairs in the trinucleotide set of a DNA sequence. A schematic representation of the sequence pattern matrix can be found in Figure 1B. Finally, the DNA sequence is converted to a 64 $\times$ 64 matrix of relationship weights for the trinucleotide pair set.

Structure of the deep learning neural network

We constructed a 2D convolutional neural network (CNN) to predict taxon information using sequence pattern matrices. The CNN has ten layers: two convolution layers (with a 7 $\times$ 7 kernel and the same padding), two max pooling layers (with a 2 $\times$ 2 filter), two dropout layers, a flatten layer and two fully connected layers followed by a softmax activation function. Here, the Conv2D layer takes the sequence pattern matrix I of dimensions $(L \times L)$ as the sequence pattern matrix and generates total F feature maps as output by corresponding F (kernels) of dimensions $k_1 \times k_2$ with the same padding. Those kernels were used to extract information on the viral sequence. Using ReLU (Rectified Linear Unit) as the activation function, the Conv2D layer output an $\mathrm{F} \times k_1 \times k_2$ matrix Y^C and computes the fth feature map at the $(i^{\mathrm{th}}, j^{\mathrm{th}})$ location, and the value is given as:

$$(Y_{ij}^C)_f = \mathrm{ReLU}\left(\sum_{m=0}^{k_1-1}\sum_{n=0}^{k_2-1} K_f(m,n) I(i-m, j-n) + b_f^C\right), \quad (2)$$

$$\text{for } i, j = 0,1,2, \dots, \mathrm{L}-1, \mathrm{f} = 0,1,2, \dots, \mathrm{F}-1.$$

Where K^f and $b_{\mathrm{f}}^{\mathrm{C}}$ are a $k_1 \times k_2$ weight matrix and a bias of the fth kernel. Mainly, the ReLU function mentioned above is defined as follows:

$$\mathrm{ReLU}(\mathrm{x}) = \begin{cases} \mathrm{x} & \text{if } \mathrm{x} \geq 0 \\ 0 & \text{if } \mathrm{x} < 0 \end{cases}. \tag{3}$$

The next layer in the model is a Maxpooling layer, taking the maximum value over an input channel with a pooling size $\mathrm{S1} \times \mathrm{S1}$ and a stride size $\mathrm{S2} \times \mathrm{S2}$. The padding option is set to “valid”. The window is shifted along with each channel independently and can generate F new channels with the size of $\mathrm{L}' \times \mathrm{L}'(\mathrm{L}' = [(\mathrm{L} - \mathrm{S1})/\mathrm{S2} + 1])$. The Maxpooling layer outputs an $\mathrm{L}' \times \mathrm{L}' \times \mathrm{F}$ feature matrix Y^M and one of the pooling operations for a specific channel at the $(i^{\mathrm{th}}, j^{\mathrm{th}})$ location was calculated as:

$$Y_{(i,j),\mathrm{f}}^{\mathrm{M}} = \max(Y_{(i\times\mathrm{S2},j\times\mathrm{S2}),\mathrm{f}}^{\mathrm{C}}, Y_{(i\times\mathrm{S2}+1,j\times\mathrm{S2}+1),\mathrm{f}}^{\mathrm{C}}, Y_{(i\times\mathrm{S2}+2,j\times\mathrm{S2}+2),\mathrm{f}}^{\mathrm{C}}, \dots, Y_{(i\times\mathrm{S2}+\mathrm{S1}\text{-}1,j\times\mathrm{S2}+\mathrm{S1}\text{-}1)\text{-}1,\mathrm{f}}^{\mathrm{C}}), \tag{4}$$

$$\text{for } i,j = 0,1,2,\dots,\mathrm{L}' - 1,\ \ \mathrm{f} = 0,1,2,\dots,\mathrm{F} - 1.$$

The features, the neural network learns in the Maxpooling layer are transferred to the Dropout layer. The output $\tilde{y}^{\mathrm{Dp}}$ is formulated as:

$$\tilde{y}^{\mathrm{Dp}} = \tilde{k} * Y^{\mathrm{M}} \quad \text{Where } \tilde{k} \sim B(1, \mathrm{P}). \tag{5}$$

Here $*$ denotes an element-wise product. For any layer Y, The drop mask $\tilde{k}$ denotes an independent Bernoulli distribution with random variables, each having a probability p of 1. It could effectively reduce overfitting. We employed Flatten layer to convert all the elements $\boldsymbol{Y}^{\mathrm{Dp}}$ in the tensor into a $\boldsymbol{Y}^{\mathrm{F}}$one-dimensional array one by one. The Dense1 layer uses the ReLU function to output R units. It has an R×F weight matrix W^{D1} and an R-dimensional bias vector b^{D1}. Each output unit is given as follows:

$$y_{\mathrm{r}}^{D1} = \mathrm{ReLu}\left(\sum_{\mathrm{f}=0}^{\mathrm{F}-1} W_{\mathrm{r,f}}^{D1}\, y_{\mathrm{f}}^{\mathrm{F}} + b_{\mathrm{r}}^{D1}\right), \text{for } \mathrm{r} = 0,1,2,\dots,\mathrm{R}-1. \tag{6}$$

The Dense1 layer can generate an R-dimensional vector $\boldsymbol{y}^{\mathrm{D1}}$ while a Conv1D layer extracts features into different feature maps, and we use a SoftMax function as an activation function. The final layer is the Dense2 layer, which outputs only a 1×2 dimension array to represent the likelihood of phages and eukaryotic viruses. The output score is calculated as follows:

$$\hat{y}_i^{D2} = \mathrm{softmax}(y_i) = \frac{e^{y_i}}{\sum_{j=1}^{n} e^{y_j}}. \tag{7}$$

Moreover, the loss function is defined below:

$$Loss = -\sum_{i=1}^{C} y_i^{D2} \cdot \log \hat{y}_i^{D2}. \tag{8}$$

We employed the Adam optimizer (learning rate = 0.0005) and batch size 16 to train the neural network and update network weights (F = 128, M = 2, S1 = S2 = 2, P = 0.32, and R = 64). The

architecture of the IPEV neural network is depicted in Figure 1C. When using IPEV, the final viral taxon scores are obtained by weighted averaging of the subsequence predictions. The detailed calculation methodology is outlined in the Supplementary Materials and Methods section.

RESULTS

Performance on viral genome fragments using cross-validation

To evaluate the performance of IPEV, we implemented a 5-fold cross-validation procedure on Groups A-D. The HTP tool, which we compared, comprises four distinct classifiers: KNN, SVC, LR, and QDA. The results showed that IPEV performed better than KNN, SVC, LR, and QDA by an average AUC (Area Under the Curve) value increase of 0.16, 0.18, 0.20, and 0.22, respectively, in Group D (1,200-1,800 bp). This is further depicted in Figure 3 and Table S4. Additionally, we observed that the performance of the model's predictions is directly proportional to the nucleotide sequence length. The AUC value of IPEV increased from 0.88 to 0.99, from Group A (100-400 bp) to Group D (1,200-1,800 bp). In contrast, the AUC value of HTP (KNN) increased from 0.66 to 0.83, as shown in Figure S1.

In binary classification, sensitivity (Sn) and specificity (Sp) evaluate the ability to predict positive and negative samples. As shown in Figure 2 and Table S3, In Group A (100–400 bp), the performance of HTP (KNN) in accurately identifying prokaryotic viruses (considered as positive samples) was found to be modest, achieving Sn of 52.6% and Sp of 71.6%. In contrast, our IPEV tool demonstrated rather better performance under the same conditions, with Sn of 78.4% and Sp of 80.9%. This suggests that IPEV outperforms HTP (KNN) in accurately classifying short viral fragments. This implies that for some species with low abundance or insufficient sequencing depth, it may not be possible to assemble longer phage sequences, and HTP may fail to identify them. IPEV, on the other hand, does not exhibit a specific preference. In Group D (1,200–1,800 bp), the performance of HTP (KNN) in accurately identifying eukaryotic viruses was found to be modest, with Sn of 80.8% and Sp of 70.1%. Conversely, under the same conditions, the IPEV tool exhibited superior performance, with Sn of 95.0% and Sp of 96.1%.

Performance on novel viruses with low homology to known databases.

Tools designed for predicting viral taxa aim to accurately identify newly discovered viruses, especially those exhibiting low homology to existing viral databases [40, 53, 54]. However, evaluating

the performance of such tools is challenging owing to the lack of accurate labels for new viral sequences. This study defines a novel virus as one with very low homology to known viruses. To evaluate IPEV's effectiveness, we constructed several independent test sets in which sequence identity increased compared to the training set of IPEV, these evaluations are designed to measure IPEV's performance on "unseen" viruses. These six low homology test sets (Dataset I1-I6) were generated with varying degrees of query coverage and identity relative to the training set. Related details are shown in the Supplementary Materials and Methods. For Dataset I1, the majority of high homology sequences were eliminated, applying a threshold of 30% identity and 30% coverage. This highly labeled and unbalanced Dataset I1 comprises Groups A, B, C, and D, containing 3,180, 3,152, 3,050, and 3,171 prokaryotic virus contigs, respectively, and 1,106, 1,117, 1,011, and 1,036 eukaryotic virus contigs, respectively.

Despite removing sequences with high homology to the training set, IPEV still outperformed HTP, as shown in Figure 4 and Table S7. Specifically, in Group A (100-400 bp) of Dataset I1, IPEV reported Sn of 76.94% and Sp of 81.19%. On the other hand, HTP (KNN) reported Sn of 55.52% and Sp of 78.55%. In Group D (1,200-1,800 bp), IPEV outperforms HTP(KNN) in Sn and Sp by 10% and 16%, respectively. These results underscore the superior performance of IPEV in distinguishing prokaryotic viruses from eukaryotic viruses. Meanwhile, we observed that as the sequence length increased from Group A (100-400 bp) to Group D (1,200-1,800 bp), the F1-score of IPEV improved from 66.61% to 89.44%. In contrast, the F1-score for HTP (KNN) increased from 51.12% to 67.81%. This performance is consistent with the results of a 5-fold cross-validation. As shown in Figure 4 and Figure 5, in terms of the AUC metric, IPEV outperformed HTP (KNN) by 0.13, 0.11, 0.11, and 0.09 in Groups A, B, C, and D, respectively. These results demonstrate the advantage of IPEV in handling short fragments and datasets with low homology.

Furthermore, to evaluate the performance of IPEV, we utilized Dataset I2-I6, each exhibiting different levels of homology to the training set. As the number of high homology sequences between the independent test set and the training set increases, we observe a gradual improvement in the performance of IPEV, as shown in Table S7-S12 and Figure S3-S7. The results indicate that the similarity between the test and training sets is crucial to determining the classification performance. Table S12 demonstrates that in Group D (1,200-1,800 bp) of Dataset I6 (Query parameter: coverage = 100% identity = 60%), the F1-score that can be achieved with IPEV is 96%. In contrast, the highest F1-score reported with HTP (KNN) is 77%.

On the other hand, it is also important to consider the impact of label imbalance on model performance. In Dataset I1-I6, which vary in their similarity to the training set of IPEV and in their respective length ranges (from Group A to Group D), IPEV's Sn and Sp did not show significant variation as the label imbalance increased, while HTP (KNN) exhibited obvious changes. This indicates that IPEV maintains robust predictive performance, even when tested on sets with low sequence homology to the training set, and does not exhibit a bias in binary classification.

Performance on test sets with sequencing errors

In this subsection, we evaluate the performance of IPEV and HTP on a dataset with varying levels of sequencing errors, including base insertion, deletion, and substitution. Errors in NGS are determined by the sequencing method and the experimental environment. Specifically, NGS has an error rate of 0.06% to 0.24% per base, while third-generation sequencing, such as PacBio, even exhibits a higher error rate of 5% to 15% per base [55, 56]. To evaluate the robustness of IPEV, we employed MetaSim to generate 2,000 short reads for both eukaryotic and prokaryotic viruses with different sequencing errors ranging from 1,200 to 1,800 bp. As shown in Table 1, the AUC values for both IPEV and HTP decrease as an increasing proportion of sequencing errors. When the error rate (base substitutions) reaches 15%, IPEV outperforms HTP (KNN) by approximately 16% in terms of the AUC metric. When the error rate (base insertions or deletions) reaches 15%, IPEV outperforms HTP (KNN) by approximately 17% in terms of the AUC metric. The observed phenomenon may be attributed to the sequence pattern matrix's capacity to tolerate errors. Notably, our results indicate that the performance of IPEV is minimally affected by the percentage of sequencing errors introduced. Specifically, when the substitution-induced error rate rises from 0% to 15%, the AUC value declines slightly from 0.99 to 0.91. These findings underscore the robustness and reliability of IPEV, showing its ability to maintain its performance despite the presence of sequencing errors.

Table 1. Comparison of IPEV and HTP's AUC value on artificial datasets (1,200-1,800 bp) with varying error rates.

Error Rate (%)	Base Substitutions		Base Insertions or Deletions	
	IPEV	HTP	IPEV	HTP
0	0.99	0.82	0.99	0.82
5	0.98	0.80	0.98	0.80

10	0.95	0.77	0.97	0.80
15	0.91	0.75	0.95	0.78

Performance on functional protein sequences

In addition to evaluating short sequence fragments, we comprehensively evaluated the capability of the IPEV tool by incorporating protein sequences with functional annotations. One key aspect of understanding virus classification is identifying critical markers that play a role in the classification process, even though deep learning is often considered a black box.

Considering the crucial role of receptor-binding proteins (RBPs) in the adsorption and host invasion of bacteriophages, we formulated a hypothesis suggesting their substantial contribution to the accuracy of phage prediction. To test this hypothesis, we carefully assembled a dataset of 7,384 RBPs, complemented with corresponding negative samples, verified manually.–we aimed to evaluate the impact of RBPs on phage prediction accuracy. The IPEV prediction results showed that RBPs significantly contributed to phage prediction accuracy. The predicted likelihood score by IPEV had a mean of 0.90 and a median of 0.98 for the RBP set, while for the non-RBP set, the mean was 0.77 and the median was 0.83, as shown in Figure 6 (Wilcoxon rank-sum test, p-value < 2.2e-16). The findings from our study also indicate that our model possesses the capability to learn host-related information to a certain degree. This emphasizes the significance of integrating host-related data into phage prediction models. To assess our model's performance in predicting eukaryotic viruses, we conducted a focused analysis of seven experimentally confirmed capping enzymes. The results revealed that our model accurately predicted the likelihood of these protein sequences, achieving a score close to 1. This result provides compelling evidence of the model's high accuracy and effectiveness in predicting eukaryotic viruses. For additional details regarding these results, please refer to Table S14.

Performance on the marine virome

We collected data from 243 marine virome samples with assembled contigs and annotated them for sequence type using Blastn. We evaluated our tool, IPEV, along with other related tools. We reported the overall average and median AUC for the samples, and we noted that our tool, IPEV, outperforms HTP (KNN, and others) across all samples. As shown in Figure 7, in our comprehensive evaluation, IPEV demonstrated its advanced capabilities by consistently outperforming other tools

with its higher average AUC values: it outperforms SVC by 0.18, exceeds QDA by 0.20, surpasses LR by 0.16, and betters KNN by 0.19. These results indicate that IPEV is a highly competitive tool.

Applying IPEV to analyze the longitudinal gut virome in a cohort study

Within this subsection, we provide a comprehensive overview of our analysis encompassing two primary aspects. Firstly, we assess the accuracy of IPEV, along with its related tool, in analyzing the gut virome. Secondly, we explore the temporal stability of healthy gut virome utilizing longitudinal data spanning a one-year period, employing the IPEV tool.

In our study, we analyzed a dataset comprising 130 samples collected from 10 subjects, which were originally obtained in Shkoporov et al.'s [50] study. We processed and annotated the raw data following the methodology outlined in the Materials and Methods section. We utilized the IPEV tool and its associated software on each distinct sample to generate predictions and compute AUC scores, leveraging BLAST annotation in the process. Our analysis, illustrated in Figure 8A, demonstrated that IPEV exhibited higher accuracy than HTP in over 90% of the real virus samples. Furthermore, IPEV's mean AUC value of 0.64 was significantly superior to those of KNN (0.55), SVC (0.51), LR (0.54), and QDA (0.51), according to the Wilcoxon rank-sum test results using Benjamini-Hochberg adjustment (IPEV with KNN: $P_{adj} < 2.89e-19$; IPEV with SVC: $P_{adj} < 1.02e-33$; IPEV with LR: $P_{adj} < 6.97e-22$; IPEV with QDA: $P_{adj} < 1.52e-36$).

Additionally, the median AUC for IPEV was 0.63, significantly higher than that of 0.54, 0.52, 0.53, and 0.51 for KNN, SVC, LR, and QDA, respectively. The median difference between the AUC of IPEV and KNN, SVC, LR, and QDA for each sample is shown in Figure 8B, with median differences of 0.09, 0.12, 0.11, and 0.12, respectively. We observed that the performance of QDA is equivalent to random guessing, with median 0.51 AUC scores. While the performance of IPEV on the simulated dataset was not as remarkable as that on the real virome data, this disparity can be attributed to a notable factor. The assembled sequences in our dataset of 130 samples predominantly comprised short sequences, with those below 500 bp representing a significant portion of 80% of the total. Coping with this challenge, our model persevered and successfully identified viruses within the real virome, demonstrating its effectiveness and robustness.

The human gut virome is characterized by its immense diversity and abundance of virus particles. The gut feces contain up to approximately 10^9 VLPs per gram, yet only a fraction of the virus genomes, ranging from 14.2% to 56.6%, can be annotated [57]. Previous research [50] has identified a highly

individualized and persistent fraction in the gut virome, the personal persistent virome (PPV). Additionally, this research has observed temporal stability in the virus components at the individual level. To further explore the virome, we employed IPEV to ab initio annotate contigs in the virome while excluding bacterial contamination. The results showed that the average coefficient of variation of the phageome was significantly lower (0.04 ± 0.01) compared to the PPV (0.58 ± 0.05), indicating a high degree of temporal stability of the phageome, as illustrated in Figure 8C and Table S15. Our findings align with the studies of Shkoporov et al. and offer further substantiation, this time at a higher taxonomic level, for the hypothesis that the "kill-the-winner" mechanism operates effectively in phage strains and substrains. This mechanism prevents the dominance of any single species, facilitating the coexistence of multiple species instead. Consequently, it enhances the diversity of the gut ecosystem and reinforces its overall resilience [58]. In contrast, the PPV is dominated by lytic life cycles and composed of virulent *CrAss-like* and *Microviridae phages* [50] that infect major representatives of the bacterial microbiota, resulting in higher variations than those seen in gut ecology.

We also observed limited transient disturbance in the phageome component in subjects 916 (T1 and T7) and 922 (T3, T5, and T8) with antibiotic usage. An intriguing observation emerged from our study: despite variations in individual characteristics such as gender, BMI, and lifestyle factors like smoking and alcohol consumption, the relative abundances of the phageome remained remarkably similar among individuals. In contrast, the relative abundances of the PPV displayed substantial individual variations. To delve deeper into these findings, we employed IPEV to assess the contribution of phage and eukaryotic viromes to the variability of PPV. Notably, our analysis revealed that phages constituted the primary component and exerted significant dominance over fluctuations in PPV as shown in Figure 8C. This highlights the crucial role of phages in driving the variability of PPV. By elucidating these relationships, our results contribute to a better understanding of the overall stability and dynamics of the gut virome.

DISCUSSION

This study introduces IPEV, a novel method that utilizes a sequence pattern matrix and a 2D convolutional neural network to distinguish prokaryotic and eukaryotic virus-derived sequence fragments. To the author's best knowledge, IPEV is the first de novo identification algorithm tool developed to address this type of problem for virome data.

IPEV offers several advantages over traditional genomics techniques, such as k-mer methods and one-hot encoding. By integrating the position and frequency information of 3-mers into a sequence pattern matrix, IPEV enhances the efficiency of the neural network model and preserves valuable information about the order and position of trinucleotides.

To ensure the generalization evaluation of the presented tool, we opted not to use the traditional approach of dividing the dataset into training and test sets based on the date the sequence was discovered [37, 59]. This method can include sequences with high homology to the training set in the test set, inflating the algorithm's accuracy and making it challenging to assess its performance accurately. Instead, we used a series of thresholds to gradually remove sequences from the test set that were homologous to the training set. During artificial contigs with less than 30% sequence homology against the training set, IPEV achieved an average AUC of 0.98, indicating that our model learned valuable information and did not rely solely on sequence similarity for prediction. Our results suggest that IPEV can generalize well and provide reliable predictions for prokaryotic and eukaryotic virus-derived sequence fragments.

Our objective was to analyze the neural network by examining its probability scores for phage prediction. The presence of the RBP is crucial for the specific interaction between the bacteriophage and its host. Therefore, we specifically focused on genes manual confirmation that are associated with RBPs, as well as an equal number of non-RBP genes. Upon comparing the score distribution of IPEV between the two datasets, we observed a notable difference. The former dataset exhibited a higher score distribution in IPEV. This discrepancy suggests that RBPs with host information could substantially influence phage prediction within the IPEV framework. As the neural network model learns from well-annotated data, IPEV can also uncover valuable insights regarding the virus-host relationship. This contributes to our overall understanding of neural networks and their performance in relevant tasks.

We utilized the IPEV approach to conduct a comprehensive analysis of longitudinal data from the gut virome, which includes eukaryotic viruses primarily acquired from the environment and diet as well as prokaryotic viruses that target microorganisms [50]. Our investigation revealed that the phage community exhibits remarkable resilience to environmental disturbances, with the "kill the winner" theory having a minimal impact at a higher taxonomic level. This finding aligns with previous research and supports the hypothesis that the "kill the winner" theory fosters a more diverse species community,

ultimately enhancing ecosystem resilience. Furthermore, we observed significant fluctuations in the abundance of PPV, primarily driven by phages that specifically target representative gut microbiota.

Although IPEV was initially designed for identifying short viral sequences, our study also encompassed an evaluation over extended lengths of 3,000 to 5,000 bp. And this length spectrum allowed us to include vConTACT v.2.0 and iPHoP in our assessment. We constructed three datasets with contig lengths of 3,000–5,000 bp (details on dataset construction and evaluation methods are provided in the Supplementary Materials and Methods). Our findings indicate that IPEV consistently outperformed the others, achieving an average F1-score of 0.99 as depicted in Figure S11. This score significantly exceeds the F1-scores of 0.02 for vConTACT v.2.0 ($t = -31.29$, $P_{adj} < 0.0001$, two-tailed independent t-tests using Benjamini-Hochberg adjustment) and 0.41 for iPHoP ($t = -395.63$, $P_{adj} < 0.0001$, two-tailed independent t-tests), respectively.

Moreover, to fulfill the demands of high-throughput data processing, we implemented a design for IPEV that enhances the efficiency of analyzing large datasets. IPEV optimizes processing time by returning all taxon prediction scores in a single iteration, requiring only four loads of the neural network weight. Unlike HTP, which expends processing time on file I/O operations, this approach allows IPEV to handle large datasets more effectively. This substantial reduction in computational overhead significantly improves the running speed of IPEV. As shown in Figure S2, IPEV operates 50 times faster than HTP, 30 times faster than vConTACT v.2.0, and 1,225 times faster than iPHoP, taking only 9.6 minutes to analyze 20,000 sequences of 1,200-1,800 bp when using the same computational resources (CPU: Intel(R) Xeon(R), 20 cores, GPU: NVIDIA Corporation GV100GL [Tesla V100 PCIe 32 GB]). We plotted the training loss, validation loss, training accuracy, and validation accuracy curves with respect to the number of epochs using 5-fold cross-validation. We observed that the model converged at 30 epochs, with training and validation losses and accuracies remaining consistent and overlapping. This indicates that the model achieved high performance while avoiding overfitting or underfitting the data, as shown in Figure S8 and Figure S9.

An important consideration when using IPEV is the potential bacterial and fungal contamination in virome datasets [60, 61]. To address this, we designed a feature within IPEV that eliminates false-positive non-viral components (bacteria and fungi). Our results show IPEV's effectiveness in differentiating viral from non-viral genome fragments in datasets where viruses and non-viruses are present in a 50:50 ratio. As shown in Figure S12, across Groups A to D, IPEV achieved Sn scores of

0.73, 0.833, 0.905, and 0.931, respectively (details on sample construction and methods can be found in Supplementary Materials and Methods). This feature is available as an optional switch. Our current tool has limitations as it does not encompass downstream virus classification or provide specific virus-host predictions for virome data. A more precise and systematic classification system is required to understand the influence viruses have on microbial communities and hosts. We eagerly anticipate the development of additional tools that will reduce the extent of virome dark matter and enhance our comprehension of the virome's intricacies.

Availability of supporting source code and requirements

Project name: IPEV

Project home page: https://github.com/basehc/IPEV.

Operating system: IPEV is platform-independent.

Programming language: Python

Other requirements: IPEV is built on Python 3.8.6 and Tensorflow 2.3.1.

License: The source code of IPEV is distributed as open source under the GNU GPL V3.

RRID:SCR_023702

Availability of data and materials

Our study contains only publicly available viral genome sequences, reference bacterial genome s equences, the IPEV program, and a detailed tutorial that can be publicly available at Zhu Lab home pa ge (https://cqb.pku.edu.cn/zhulab/info/1006/1156.htm) or GitHub (https://github.com/basehc/IPEV) un der GNU General Public License V3. Code, data and Docker image for transparent and reproducible r esults were documented on Zenodo (https://doi.org/10.5281/zenodo.10118192).

Additional files

Supplementary Materials for this manuscript include the following:

Table S1: Details the distribution of contigs across various length ranges within a 5-fold cross-validation dataset based on Virus-Host DB.

Table S2: Number of simulated contigs based on RVDB and IMG/VR v4 databases.

Table S3: The average performance of IPEV and HTP (KNN) with 5-fold cross-validation for various sequence lengths, expressed as a percentage.

Table S4: The average AUC value for four length groups on a 5-fold cross-validation.

Table S5: Enumerates the numbers of the prokaryotic virus contigs in Dataset I1-I6.

Table S6: Enumerates the numbers of the eukaryotic virus contigs in Dataset I1-I6.

Table S7-S12: Compare the performance of IPEV and HTP under various query parameters for coverage and identity in Dataset I1-I6.

Table S13: Performance of IPEV and HTP on the ducks' gut virome data a total of 682 eukaryotic contigs and 1453 prokaryotic virus contigs).

Table S14: Likelihood scores predicted by IPEV algorithm on capping enzymes. And Table S15: P-values show the phageome's average coefficient of variation was significantly lower compared to the PPV (Wilcoxon rank-sum test, adjusted using the Benjamini-Hochberg correction).

Figure S1: The ROC curves and AUC value of IPEV and HTP performances in each set of 5-fold cross-validation.

Figure S2: Performance of IPEV, HTP, vConTACT2, and iPHoP as the number of sequences increases under the same computing configuration (1,200-1,800 bp).

Figure S3-S7: Comparison of IPEV and HTP on the Dataset I2-I6.

Figure S8: Mean accuracy, mean loss, and mean of IPEV's 5-cross-validation on Group A.

Figure S9: The mean validation loss and accuracy of IPEV's 5-cross-validation on Group A.

Figure S10: A. Performance (accuracy, specificity, sensitivity, precision, and F1-score) of IPEV and HTP (KNN, SVC, LR, QDA) on the duck gut virome data. B. The ROC curve and AUC value of IPEV and HTP (KNN, SVC, LR, QDA) on the duck gut virome data.

Figure S11. Average performance of IPEV, HTP, iPHoP, and vConTACT v.2.0 across three independent test sets with sequence lengths of 3,000-5,000 bp and Figure S12. the confusion matrices show the false positive reduction capability of IPEV on datasets with a 1:1 ratio of viruses to non-viruses across Groups A to D.

Author contributions statements

H.Q.Z., H.C.Y. and S.F.W. conceived and designed the project; H.C.Y., J.T. and S.F.W. constructed the datasets; H.C.Y. and S.F.W. wrote and optimized the model of IPEV; H.C.Y. and S.F.W. performed the data analysis and design of the pipeline and prepared all the figures and tables; H.C.Y. and H.Q.Z. drafted the manuscript. H.Q.Z., H.C.Y., S.F.W., Q.G., M.L., J.Y.G., Y.Q. W., X.Q.J., and J.T. revised and edited the manuscript, and all authors proofread and improved the manuscript.

Acknowledgments

This work was supported by the National Key Research and Development Program of China (2021YFC2300300) and the National Natural Science Foundation of China (32070667, 31671366, 32300078). Part of the analysis was performed on the High-Performance Computing Platform of Peking University. We thank Tianze Wang of Peking University for helpful discussions

Conflict of interest

The authors declare that there is no conflict of interest.

Tables and Figures Legends

Figure 1. Workflow for extracting the sequence pattern matrix and using a deep learning neural network structure to predict taxon. **A.** The virus genomes are initially divided into five subsets, and then each subset is simulated to represent four groups with different contig lengths. **B.** Overlapping trinucleotides are used to represent the virus contigs. For example, if the nucleotides of the viral fragment are "ATTCATAACTT", the trinucleotide set would consist of "ATT, TTC, TCA, CAT, ATA, TAA, AAC, ACT, CTT". The trinucleotide set is then converted to a 64x64 sequence pattern matrix using a sequence pattern function. **C.** The IPEV tool employs a 2D CNN model as the classifier. The CNN model accepts the sequence pattern matrix as input and outputs a 1x2 array representing the likelihood of prokaryotic and eukaryotic viruses.

Figure 2. Panels A, B, C, and D display the comparative performance of IPEV and HTP (KNN, SVC, LR, and QDA) with 5-fold cross-validation across Groups A, B, C, and D, respectively. * Sn = TP / (TP + FN), Sp = TN / (TN + FP), ACC = (TP + TN) / (TP + TN + FP + FN), Precision = TP / (TP + FP), F1-score = 2 × Precision × Recall / (Precision + Recall), where TP, TN, FP, and FN respectively represent true positive, true negative, false positive, and false negative. As the method with the best performance in HTP, KNN is selected for comparison. The mean and standard deviation of 5-fold cross-validation are computed to elaborate on performance evaluation. Due to a lack of reconstruction between the train and validation set, the performance of HTP (KNN) is overestimated. (In this paper, prokaryotic viruses are treated as positive samples.)

Figure 3. The Average performance of IPEV and HTP (KNN) with 5-fold cross-validation for various sequence lengths, expressed as a percentage.

Figure 4. Panels A, B, C, and D display the comparative performance of IPEV and HTP (KNN) across Groups A, B, C, and D, respectively, of Dataset I1. (parameter: query coverage = 30%, identity = 30%).

Figure 5. Performance comparison between IPEV and HTP (KNN) on Dataset I1. A. The ROC (Receiver operating characteristic) curve demonstrates the discrimination capability, particularly in class-balanced test sets, with higher

AUC values preferred. B. The Precision-Recall curves measure discrimination capability in class-imbalanced test sets, with AP representing the Average Precision.

Table 1. Comparison of IPEV and HTP's AUC value on artificial datasets (1,200-1,800 bp) with varying error rates.

Figure 6. Histogram illustrating the predicted likelihood scores generated by IPEV for receptor-binding proteins (RBPs) and non-RBPs.

Figure 7. A. Box plots representing AUC scores of the ROC curves for IPEV, KNN, SVC, LR, and QDA. **B.** Violin plots displaying the AUC scores differences of each tool relative to IPEV.

Figure 8. **A.** Box plots representing the AUC scores of ROC curves for IPEV, KNN, SVC, LR, and QDA. **B.** Violin plots displaying the AUC scores differences of each tool relative to IPEV. **C.** Relative abundances of phages, PPV, and PPV-associated phages in the longitudinal data of subjects 916-925 as determined by IPEV (Details of the annotations can be found in the Supplementary Materials and Methods).

References

1. Mushegian AR. Are There 1031 Virus Particles on Earth, or More, or Fewer?, *Journal of Bacteriology.* 2020;202(9).
2. Mya B, Forest R. Here a virus, there a virus, everywhere the same virus?, *Trends Microbiol.* 2005;13(6):278-84.
3. Carding SR, Davis N, Hoyles L. Review article: the human intestinal virome in health and disease, *Aliment. Pharmacol. Ther.* 2017;46(9):800-15.
4. Reyes A, Semenkovich NP, Whiteson K et al. Going viral: next-generation sequencing applied to phage populations in the human gut, *Nat. Rev. Microbiol.* 2012;10(9):607-17.
5. Santiago-Rodriguez TM, Hollister EB. Human Virome and Disease: High-Throughput Sequencing for Virus Discovery, Identification of Phage-Bacteria Dysbiosis and Development of Therapeutic Approaches with Emphasis on the Human Gut, *Viruses.* 2019;11(7):656.
6. Mandal RS, Saha S, Das S. Metagenomic surveys of gut microbiota, *Genomics Proteomics Bioinf.* 2015;13(3):148-58.
7. Lim ES, Zhou Y, Zhao G et al. Early life dynamics of the human gut virome and bacterial microbiome in infants, *Nature Medicine.* 2015;21(10):1228-34.
8. Edwards RA, McNair K, Faust K et al. Computational approaches to predict bacteriophage–host relationships, *FEMS Microbiology Reviews.* 2016;40(2):258-72.

9. Wu S, Fang Z, Tan J et al. DeePhage: distinguishing virulent and temperate phage-derived sequences in metavirome data with a deep learning approach, *GigaScience.* 2021;10(9):giab056.
10. Hall AJ. Noroviruses: The Perfect Human Pathogens?, *The Journal of Infectious Diseases.* 2012;205(11):1622-24.
11. Anderson EJ, Weber SG. Rotavirus infection in adults, *Lancet Infect. Dis.* 2004;4(2):91-99.
12. Bosch A, Pintó RM, Guix S. Human Astroviruses, *Clin. Microbiol. Rev.* 2014;27(4):1048-74.
13. Feng Z, Hensley L, McKnight KL et al. A pathogenic picornavirus acquires an envelope by hijacking cellular membranes, *Nature.* 2013;496(7445):367-71.
14. Ghebremedhin B. Human adenovirus: Viral pathogen with increasing importance, *Eur J Microbiol Immunol.* 2014;4(1):26-33.
15. Pfeiffer JK, Virgin HW. Viral immunity. Transkingdom control of viral infection and immunity in the mammalian intestine, *Science.* 2016;351(6270):aad5872.
16. Conceição-Neto N, Deboutte W, Dierckx T et al. Low eukaryotic viral richness is associated with faecal microbiota transplantation success in patients with UC, *Gut.* 2018;67(8):1558-59.
17. Jones MK, Watanabe M, Zhu S et al. Enteric bacteria promote human and mouse norovirus infection of B cells, *Science.* 2014;346(6210):755-9.
18. Metzger RN, Krug AB, Eisenacher K. Enteric Virome Sensing-Its Role in Intestinal Homeostasis and Immunity, *Viruses.* 2018;10(4):146.
19. Rose R, Constantinides B, Tapinos A et al. Challenges in the analysis of viral metagenomes, *Virus Evol.* 2016;2(2):vew022.
20. Jenkins C, Ling CL, Ciesielczuk HL et al. Detection and identification of bacteria in clinical samples by 16S rRNA gene sequencing: comparison of two different approaches in clinical practice, *J. Med. Microbiol.* 2012;61(Pt 4):483-88.
21. Federhen S. The NCBI Taxonomy database, *Nucleic Acids Research.* 2011;40(D1):D136-D43.
22. Yuan D, Ahamed A, Burgin J et al. The European Nucleotide Archive in 2023, *Nucleic Acids Res.* 2023; gkad1067.
23. Krishnamurthy SR, Wang D. Origins and challenges of viral dark matter, *Virus Res.* 2017;239:136-42.
24. Tan J, Fang Z, Wu S et al. HoPhage: an ab initio tool for identifying hosts of phage fragments from metaviromes, *Bioinformatics.* 2021;38(2):543-45.

25. Roux S, Camargo AP, Coutinho FH et al. iPHoP: An integrated machine learning framework to maximize host prediction for metagenome-derived viruses of archaea and bacteria, *PLoS Biol.* 2023;21(4):e3002083.

26. Coutinho FH, Zaragoza-Solas A, Lopez-Perez M et al. RaFAH: Host prediction for viruses of Bacteria and Archaea based on protein content, *Patterns (N Y).* 2021;2(7):100274.

27. Galiez C, Siebert M, Enault F et al. WIsH: who is the host? Predicting prokaryotic hosts from metagenomic phage contigs, *Bioinformatics.* 2017;33(19):3113-14.

28. Shang J, Sun Y. CHERRY: a Computational metHod for accuratE pRediction of virus–pRokarYotic interactions using a graph encoder–decoder model, *Briefings Bioinf.* 2022; *23*(5), bbac182.

29. Lu C, Zhang Z, Cai Z et al. Prokaryotic virus host predictor: a Gaussian model for host prediction of prokaryotic viruses in metagenomics, *BMC Biology.* 2021;19(1):5.

30. Wang W, Ren J, Tang K et al. A network-based integrated framework for predicting virus–prokaryote interactions, *NAR: Genomics Bioinf.* 2020;2(2).

31. McNair K, Bailey BA, Edwards RA. PHACTS, a computational approach to classifying the lifestyle of phages, *Bioinformatics.* 2012;28(5):614-8.

32. Song K. Classifying the Lifestyle of Metagenomically-Derived Phages Sequences Using Alignment-Free Methods, *Frontiers in Microbiology.* 2020;11:567769.

33. Fang Z, Tan J, Wu S et al. PPR-Meta: a tool for identifying phages and plasmids from metagenomic fragments using deep learning, *GigaScience.* 2019;8(6).

34. Ren J, Song K, Deng C et al. Identifying viruses from metagenomic data using deep learning, *Quant Biol.* 2020;8(1):64-77.

35. Kieft K, Zhou Z, Anantharaman K. VIBRANT: automated recovery, annotation and curation of microbial viruses, and evaluation of viral community function from genomic sequences, *Microbiome.* 2020;8(1):90.

36. Bin Jang H, Bolduc B, Zablocki O et al. Taxonomic assignment of uncultivated prokaryotic virus genomes is enabled by gene-sharing networks, *Nat Biotechnol.* 2019;37(6):632-39.

37. Roux S, Enault F, Hurwitz BL et al. VirSorter: mining viral signal from microbial genomic data, *PeerJ.* 2015;3:e985.

38. Guo J, Bolduc B, Zayed AA et al. VirSorter2: a multi-classifier, expert-guided approach to detect diverse DNA and RNA viruses, *Microbiome.* 2021;9(1):37.

39. Wang D. 5 challenges in understanding the role of the virome in health and disease, *PLoS Pathog.* 2020;16(3):e1008318.

40. Galan W, Bak M, Jakubowska M. Host Taxon Predictor - A Tool for Predicting Taxon of the Host of a Newly Discovered Virus, *Sci. Rep.* 2019;9(1):3436.

41. Greninger AL. A decade of RNA virus metagenomics is (not) enough, *Virus Res.* 2018;244:218-29.

42. Mihara T, Nishimura Y, Shimizu Y et al. Linking Virus Genomes with Host Taxonomy, *Viruses.* 2016;8(3):66.

43. Schoch CL, Ciufo S, Domrachev M et al. NCBI Taxonomy: a comprehensive update on curation, resources and tools, *Database (Oxford).* 2020; baaa062.

44. Goodacre N, Aljanahi A, Nandakumar S et al. A Reference Viral Database (RVDB) To Enhance Bioinformatics Analysis of High-Throughput Sequencing for Novel Virus Detection, *mSphere.* 2018;3(2).

45. Camargo AP, Nayfach S, Chen IA et al. IMG/VR v4: an expanded database of uncultivated virus genomes within a framework of extensive functional, taxonomic, and ecological metadata, *Nucleic Acids Res.* 2023;51(D1):D733-D43.

46. Richter DC, Ott F, Auch AF et al. MetaSim—A Sequencing Simulator for Genomics and Metagenomics, *PloS One.* 2008;3(10):e3373.

47. Jais PH, Decroly E, Jacquet E et al. C3P3-G1: first generation of a eukaryotic artificial cytoplasmic expression system, *Nucleic Acids Res.* 2019;47(5):2681-98.

48. Rangel-Pineros G, Almeida A, Beracochea M et al. VIRify: an integrated detection, annotation and taxonomic classification pipeline using virus-specific protein profile hidden Markov models, *PLoS Comput. Biol.* 2023;19(8):e1011422.

49. Gregory AC, Zayed AA, Conceição-Neto N et al. Marine DNA viral macro-and microdiversity from pole to pole, *Cell.* 2019;177(5):1109-23. e14.

50. Shkoporov AN, Clooney AG, Sutton TDS et al. The Human Gut Virome Is Highly Diverse, Stable, and Individual Specific, *Cell Host Microbe.* 2019;26(4):527-41.e5.

51. Bankevich A, Nurk S, Antipov D et al. SPAdes: a new genome assembly algorithm and its applications to single-cell sequencing, *J. Comput. Biol.* 2012;19(5):455-77.

52. Ranjan C, Ebrahimi S, Paynabar KJapa. Sequence graph transform (SGT): A feature extraction function for sequence data mining, *Data Mining and Knowledge Discovery* 2022;36(2):668-708.

53. Mock F, Viehweger A, Barth E et al. VIDHOP, viral host prediction with deep learning, *Bioinformatics.* 2020;37(3):318-25.

54. Bahir I, Fromer M, Prat Y et al. Viral adaptation to host: a proteome - based analysis of codon usage and amino acid preferences, *Mol. Syst. Biol.* 2009;5(1):311.

55. Dohm JC, Peters P, Stralis-Pavese N et al. Benchmarking of long-read correction methods, *NAR: Genomics Bioinf.* 2020;2(2):lqaa037.

56. Pourmohammadi R, Abouei J, Anpalagan A. Error analysis of the PacBio sequencing CCS reads, *The International Journal of Biostatistics.* 2023.

57. Roux S, Hallam SJ, Woyke T et al. Viral dark matter and virus-host interactions resolved from publicly available microbial genomes, *elife.* 2015;4:e08490.

58. Garmaeva S, Gulyaeva A, Sinha T et al. Stability of the human gut virome and effect of gluten-free diet, *Cell Rep.* 2021;35(7):109132.

59. Ren J, Ahlgren NA, Lu YY et al. VirFinder: a novel k-mer based tool for identifying viral sequences from assembled metagenomic data, *Microbiome.* 2017;5(1):69.

60. Zhang F, Zuo T, Yeoh YK et al. Longitudinal dynamics of gut bacteriome, mycobiome and virome after fecal microbiota transplantation in graft-versus-host disease, *Nat. Commun.* 2021;12(1):65.

61. Zolfo M, Pinto F, Asnicar F et al. Detecting contamination in viromes using ViromeQC, *Nat. Biotechnol.* 2019;37(12):1408-12.

Figure_1

Click here to access/download;Figure;Figure_1.jpg

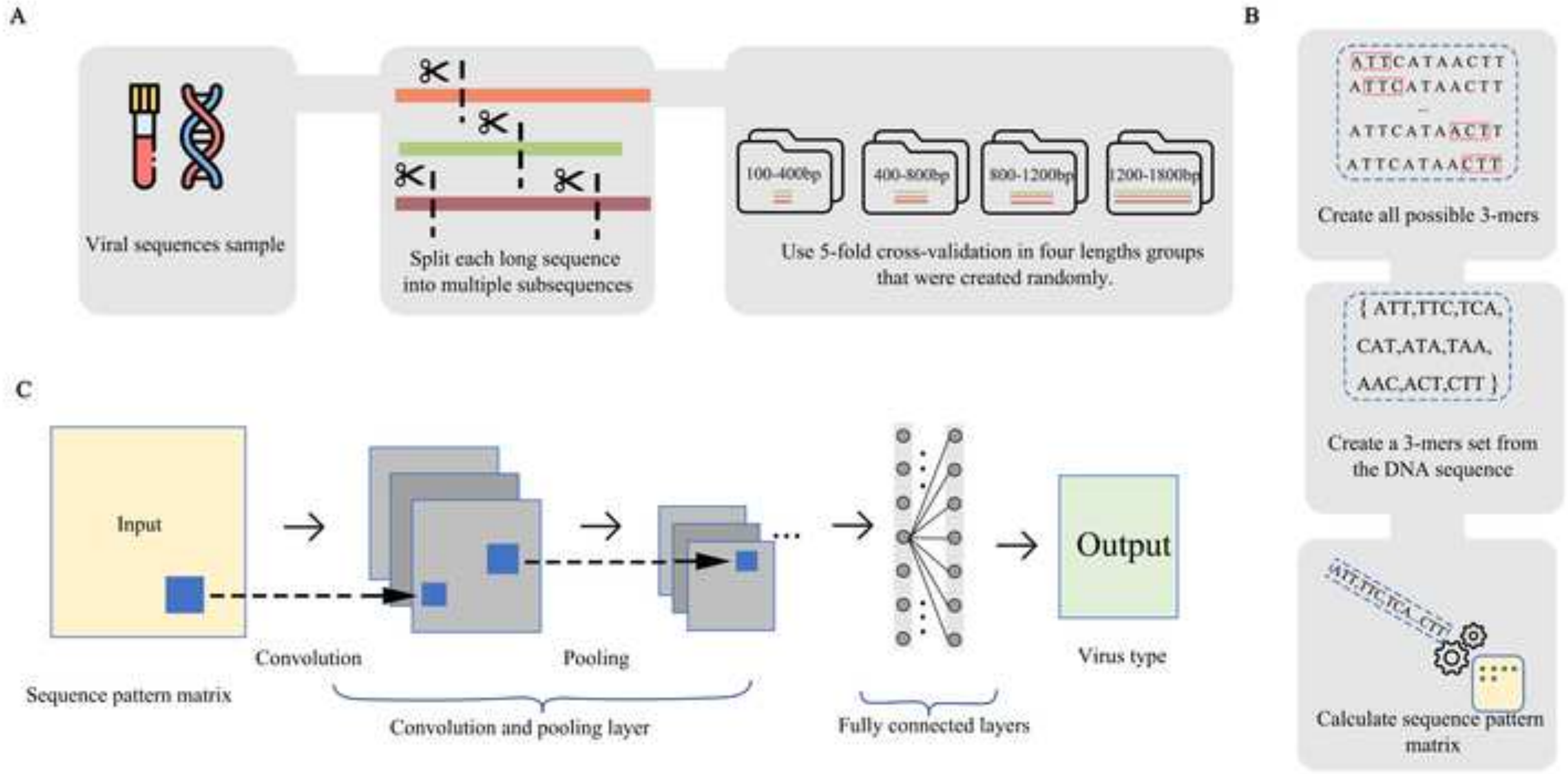

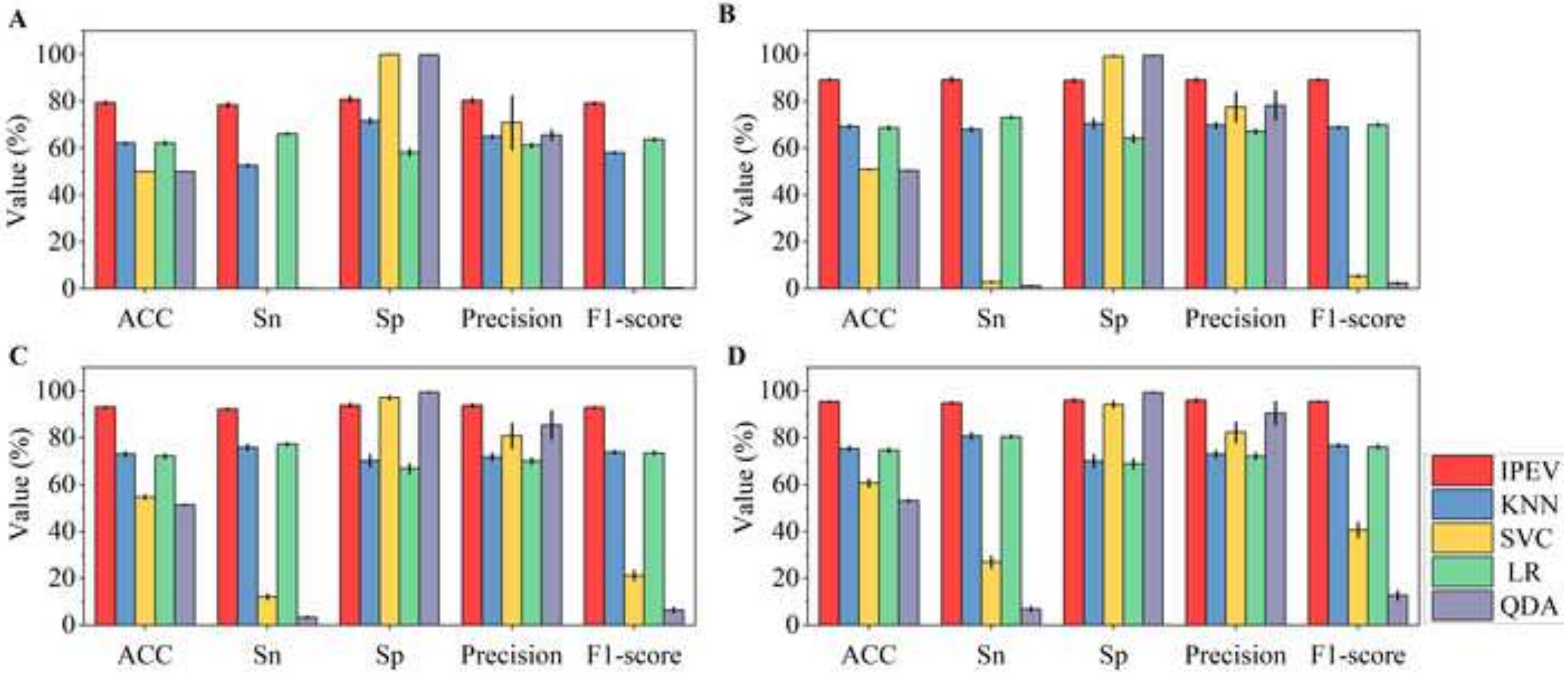
A
B
C
D
Value (%)
0
20
40
60
80
100
ACC
Sn
Sp
Precision
F1-score
IPEV
KNN
SVC
LR
QDA

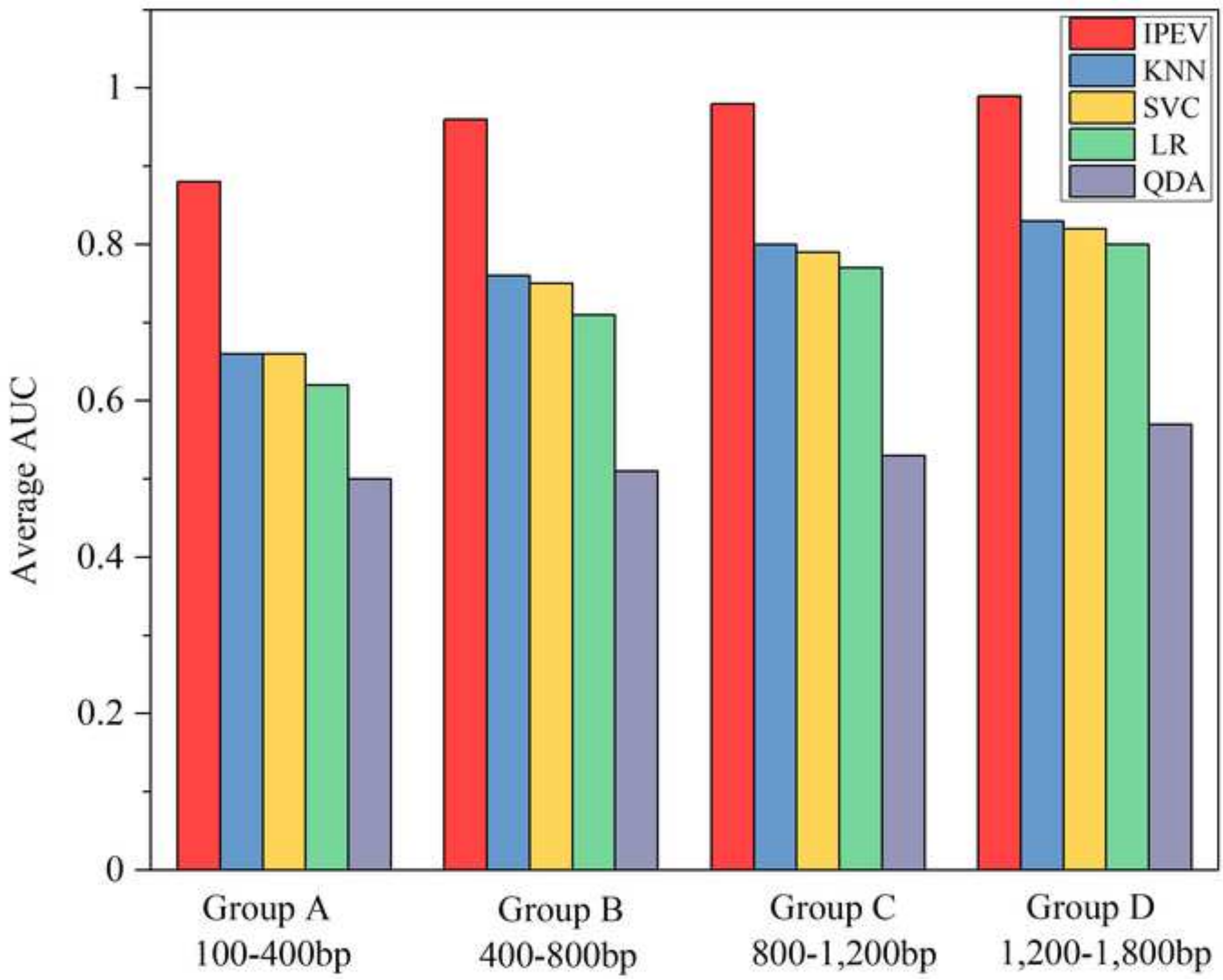

IPEV
KNN
SVC
LR
QDA
Average AUC
0
0.2
0.4
0.6
0.8
1
Group A
100-400bp
Group B
400-800bp
Group C
800-1,200bp
Group D
1,200-1,800bp

Figure_4_revised

Click here to access/download;Figure;Figure_4.jpg

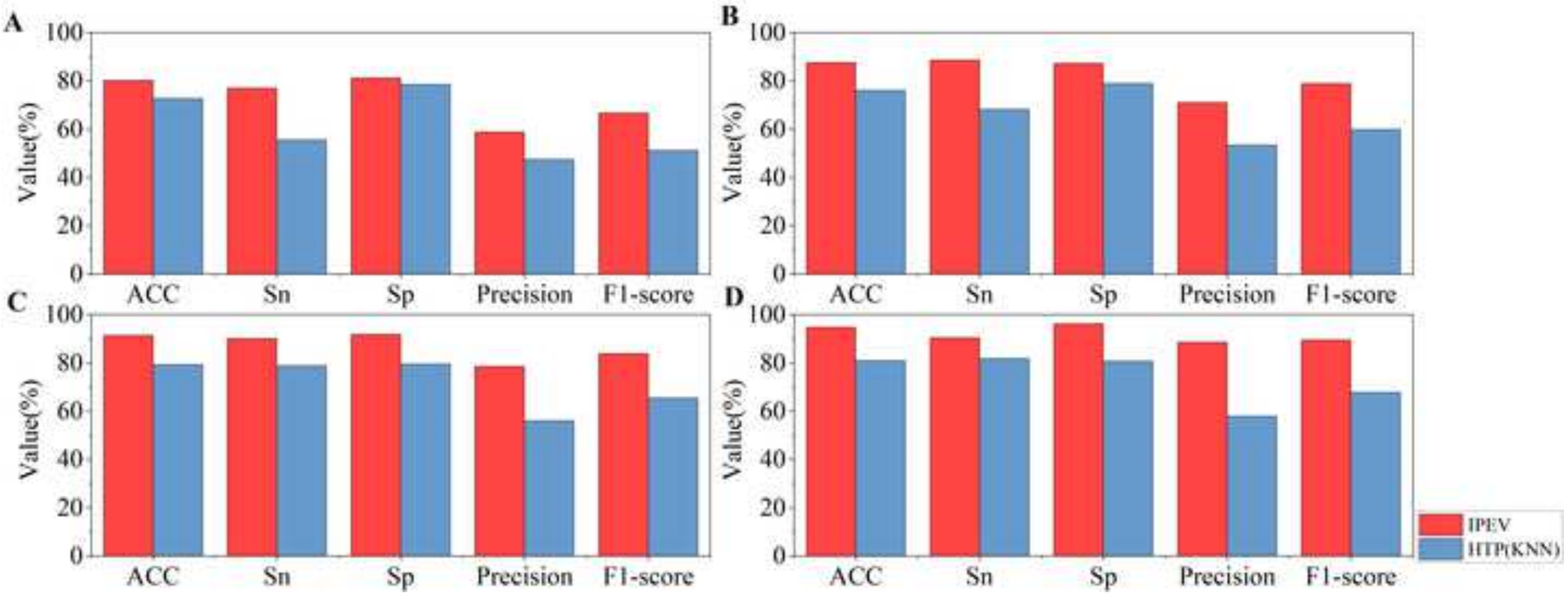

Figure_5

Click here to access/download;Figure;Figure_5.jpg

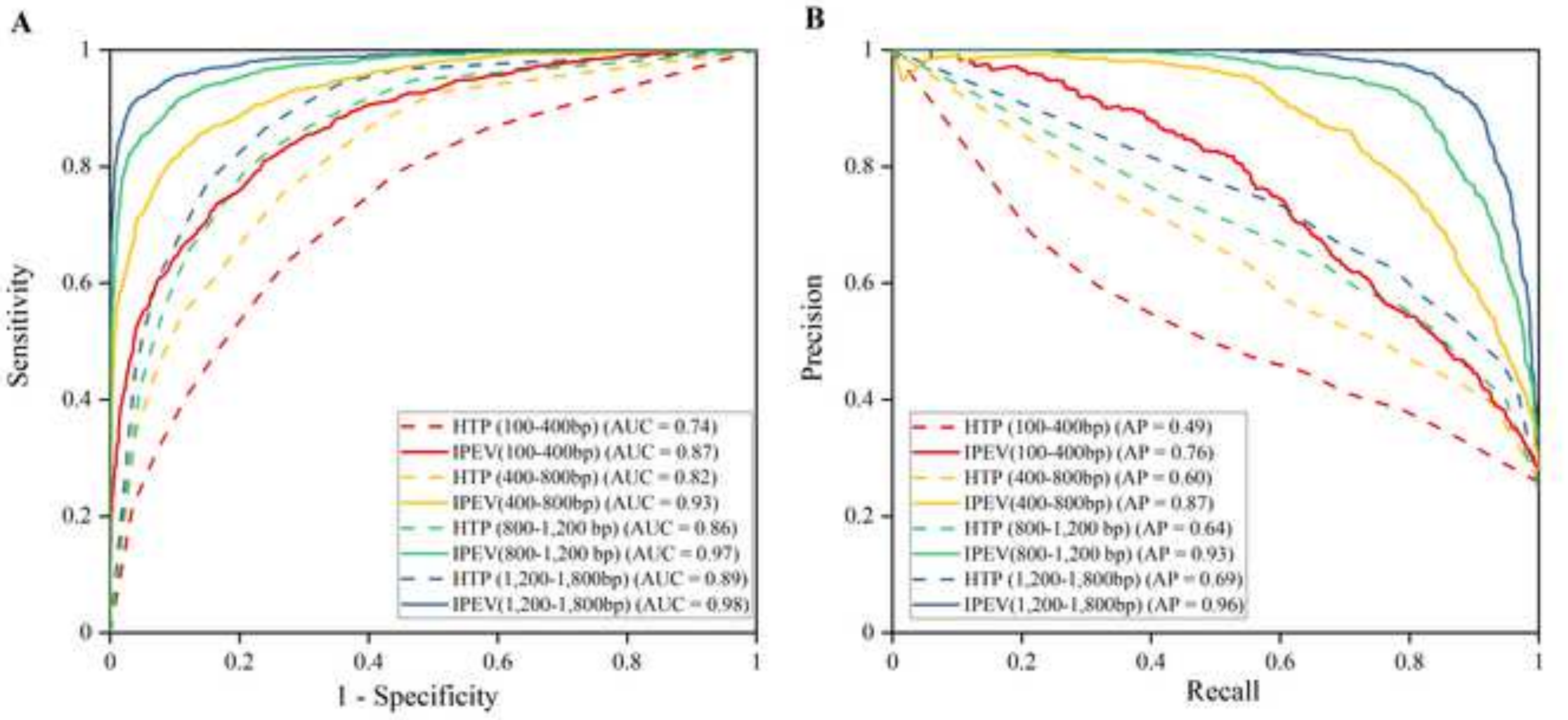

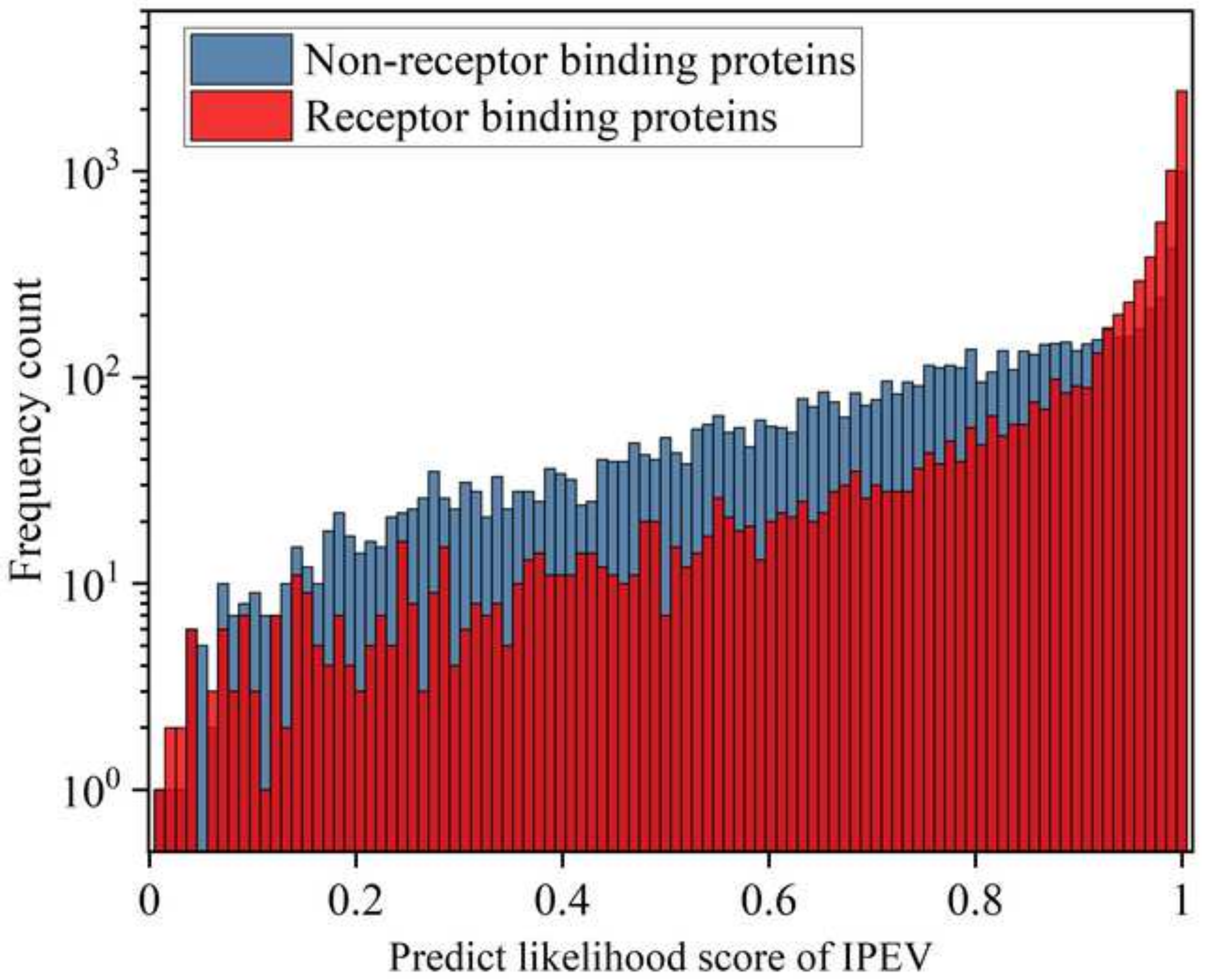
Non-receptor binding proteins
Receptor binding proteins
10^3
10^2
10^1
10^0
Frequency count
0
0.2
0.4
0.6
0.8
1
Predict likelihood score of IPEV

Figure_7_revised

Click here to access/download;Figure;Figure_7.jpg

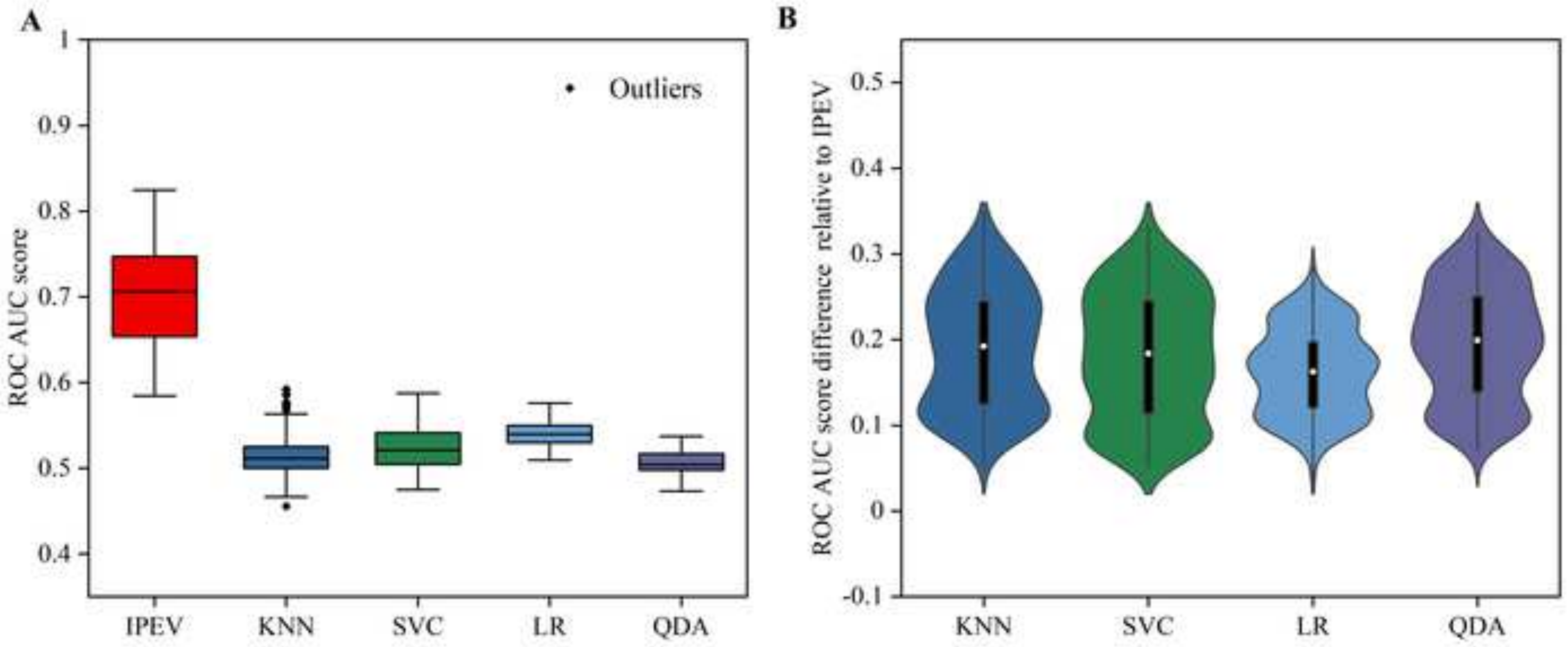

Figure_8

Click here to access/download;Figure;Figure_8.jpg

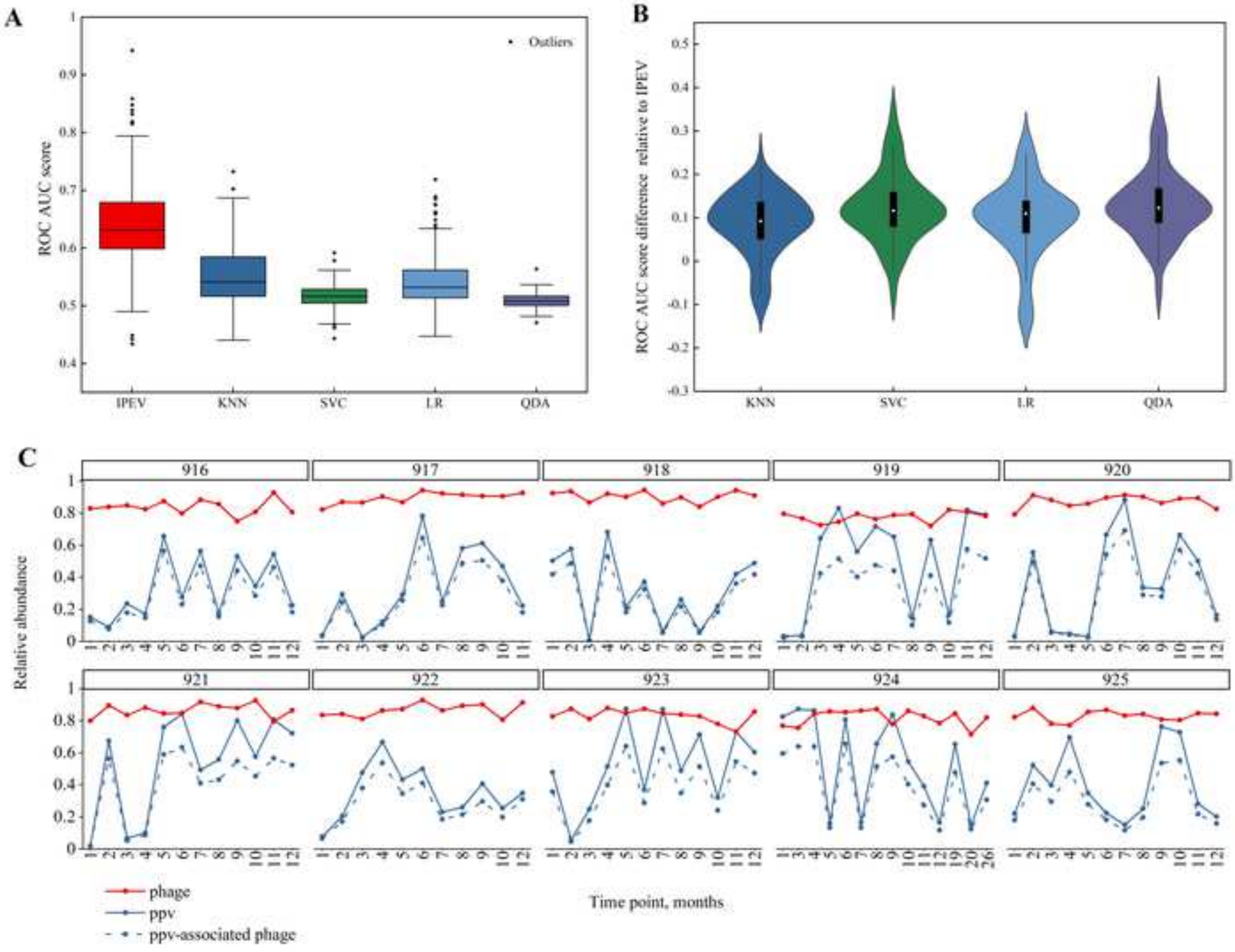

Click here to access/download
Supplementary Material
IPEV_R1_Supplementary_Materials.docx

Cover_Letter_for_Resubmission

Click here to access/download;Personal Cover;Cover_Letter_Resubmission.docx

Cover Letter

Dear Editor,

Thank you very much for your previous E-mail on September 11, 2023, regarding our manuscript, "IPEV: identification of prokaryotic and eukaryotic virus-derived sequences in virome using deep learning" (Manuscript ID: GIGA-D-23-00176). We first want to thank you very much for providing the opportunity to revise and resubmit our work. Meanwhile, we are really appreciative of two Reviewers' positive comments about our work of that it "… *represents a novel method*" and "… *efficiently covers a wide range of sequence lengths*", and "… *offers a potential benefit to the community*". Moreover, we appreciate so much substantial and valuable advice and comments from you and two Reviewers, which absolutely helped us improve our work.

Following your advice and two Reviewers' comments, we have made an effort to revise the manuscript substantially (both the main document and Supplementary Materials). In this cover letter and the revised manuscript, we used red text for all the changed words, sentences, or paragraphs. Detailed responses to Reviewers' comments are provided in a point-by-point manner below. Before we report our responses to Reviewers' comments, we would like to first summarize the main changes in the revised manuscript as follows:

1. We integrated two additional data sets for cross-validation purposes, in addition to the reference sequences, and concurrently adjusted the hyperparameters of the neural network in IPEV.

To improve the generalization capabilities of the IPEV model, we incorporated two additional data sets as part of the 5-fold cross-validation process, as recommended by Reviewer 2. This included 25,644 eukaryotic sequences from the RVDB database (Goodacre et al., mSphere, 2018) and 5,598 prokaryotic sequences from IMG/VR v4 (Camargo et al., Nucleic Acids Res, 2023). In response to the increased dataset volume, we proportionally expanded the capacity of our neural network by enhancing both the number and size of the filters, thereby increasing the parameter count from 1,152,898 to 2,906,690. Following these adjustments, we re-conducted 5-fold cross-validation on Group A (100–400 bp) - Group D (1,200–1,800 bp), re-evaluated IPEV and related tools on independent test sets with different levels of homology against the training set of IPEV, data sets

with sequencing errors, data sets with functional proteins, real virome data, and re-evaluated the stability of the gut virome using the most recent version of IPEV.

We observed that these changes resulted in an improvement in our tool's performance in metrics like the F1-score, both on the 5-fold cross-validation process and gut virome. Additionally, our findings regarding functional proteins and the stability of the gut virome remain unchanged. We deeply appreciate the reviewers' insights and suggestions.

The corresponding revisions are detailed in Section "MATERIALS AND METHODS", and Section "RESULTS" in the revised manuscript, along with Section "Supplementary Materials and Methods" in Supplementary Materials.

2. We manually curated a new independent test set that includes longer sequences for a more comprehensive evaluation and specifically incorporated the tools vConTACT v.2.0 and iPHoP to conduct this assessment.

Following Reviewer 1's suggestion, we incorporated evaluation tools, including vConTACT v.2.0 (Jang et al., Nat Biotechnol, 2019) and iPHoP (Roux et al., PLoS Biol, 2023). We proceeded to manually simulate independent test sets of 3,000-5,000 bp lengths (each containing 500 eukaryotic virus genomes and 500 prokaryotic virus genomes) three times (the reason for this approach was explained in our point-by-point response to Reviewer 1). Using tools including IPEV, HTP, vConTACT v.2.0, and iPHoP, evaluations were performed, and average metrics were computed and reported.

Our findings revealed that IPEV consistently exhibited superior performance, achieving an average F1-score of 0.99. This score significantly surpasses the F1-scores of 0.02 ($t = -31.29$, $P_{adj} < 0.0001$, two-tailed independent t-tests) and 0.41 ($t = -395.63$, $P_{adj} < 0.0001$, two-tailed independent t-tests) for vConTACT v.2.0 and iPHoP, respectively. We also observed that under identical configurations and data sets, IPEV operates 50 times faster than HTP, 30 times faster than vConTACT v.2.0, and 1,225 times faster than iPHoP. We deeply appreciate Reviewers' invaluable suggestions.

The corresponding revisions are detailed in Section "DISCUSSION" in the revised manuscript and Section "Supplementary Materials and Methods" in Supplementary Materials.

3. We extended the validation set of IPEV to marine viromes, thereby demonstrating its applicability beyond gut viromes.

Following Reviewer 2's suggestion, we downloaded a data set comprising 243 marine virome samples from the European Nucleotide Archive (accession number: PRJEB22493 (Rangel-Pineros et al., PLOS Computational Biology, 2023)), which is part of the Global Ocean Viromes 2.0 (GOV 2.0) dataset (Gregory et al., Cell, 2019), with a combined size of approximately 166 GB, to evaluate the efficacy of IPEV alongside comparable tools. Initially, we used BLASTn against reference viral genomes with an e-value threshold of e-5 for annotation, which allowed us to assign genomes with eukaryotic and prokaryotic virus labels. On these 243 marine virome samples, we calculated the Area Under the Curve (AUC) scores for each tool individually.

We reported the overall average and median AUC value for each sample, and we noted that our tool, IPEV, outperforms HTP (KNN and others) across all samples. In our comprehensive evaluation, IPEV demonstrated its advanced capabilities by consistently outperforming other tools with its higher average AUC values: it outperforms SVC by 0.18, exceeds QDA by 0.20, surpasses LR by 0.16, and betters KNN by 0.19. These results indicate that IPEV is a highly competitive tool. We are very grateful to Reviewer for the insightful advice.

The corresponding revisions are detailed in Section "MATERIALS AND METHODS" and Section "RESULT" in the revised manuscript.

4. We integrated a contaminant filtration feature into IPEV, which is designed to effectively remove false-positive samples originating from prokaryotic and eukaryotic contaminants in virome data sets.

In response to Reviewer 2's suggestions and requirements, we have developed an additional algorithm that distinguishes between viral and non-viral components (bacteria and fungi) using the sequence pattern matrix and a neural network framework within IPEV. We performed searches on the NCBI Assembly database using the commands "(Fungi[orgn]) AND (representative genome[filter])" and "(Bacteria[orgn]) AND (representative genome[filter])" to download data sets. We downloaded 1,428 bacterial genome sequences, ensuring to exclude plasmids and include only those labeled as "complete", and 99 fungal sequences, excluding macrofungi. Using MetaSim, we simulated contigs to create negative sample groups for both bacterial and fungal data sets separately:

1 million for Group A (100-400 bp), 900,000 for Group B (400-800 bp), 800,000 for Group C (800-1,200 bp), and 700,000 for Group D (1,200-1,800 bp). Based on the sequence pattern matrix and neural network framework outlined in our manuscript, we retrained, validated, and tested on the constructed Groups A to D using an 8:1:1 split for training, validation, and testing, respectively.

In this experiment, we constructed test sets with an equal number of virus-like and non-virus-like organisms (bacteria and fungi) in Group A - Group D. We then calculated the sensitivity (Sn) and specificity (Sp) for each group. For viral identification, Sn rates were found to be 73.1% for Group A, 83.3% for Group B, 90.5% for Group C, and 93.1% for Group D. Correspondingly, for non-viral identification, Sp rates were 84.5% for Group A, 93.7% for Group B, 95.7% for Group C, and 97.3% for Group D. This enhanced functionality, designed to reduce false-positive (non-viral) samples, has been integrated into the IPEV tool, complete with a user-activated switch. We are grateful for Reviewer's invaluable advice.

The corresponding revisions are detailed in Section "DISCUSSION" in the revised manuscript, and Section "Supplementary Materials and Methods" in Supplementary Materials.

Besides, following the requirements from our previous submission, we have updated the data and code in the following links. Furthermore, we have incorporated the Zenodo data location description into the Data Availability section of our manuscript.

Below are the locations that contain the latest versions, including the data, code, analysis, and supplemental files:

(1) https://doi.org/10.5281/zenodo.10118192 This contains collection of datasets for training, validation, and testing as detailed in the manuscript, along with a Docker image that holds all the essential data and codes for repeatable results. (To simplify the process of obtaining the data, the previous link (https://github.com/basehc/IPEV_analysis/tree/main) has been removed, and its contents have been integrated into the Zenodo link.)

(2) https://github.com/basehc/IPEV This is the official website for the IPEV tool.

(3) https://dome.ds-wizard.org/projects/5e3673f9-217c-474e-a5e7-91c6768638ad This is the data and method illustration of IPEV, as required by GigaScience.

(4) RRID:SCR_023702

(5) and any manuscript supplemental files.

More details are listed in the following point-by-point responses to the reviewers' comments. We then report our revisions and responses to all two Reviewers' comments (*italic text*) one by one as follows:

To Reviewer 1:

General Comments: *Yin et al. have developed a new tool to differentiate eukaryotic and prokaryotic viruses. The tool offers a potential benefit to the community, but there are several issues with the contribution in its current form, as discussed below.*

Response:

We are deeply grateful for Reviewer 1's constructive feedback. Herein, we are glad to see Reviewer 1's positive comments on the present work, as our tool "*... offers a potential benefit to the community*". We especially thank Reviewer 1 for the careful reading of our manuscript. The suggestions and comments raised by Reviewer 1 were certainly very valuable and helpful for us to improve the manuscript. Below, we itemize the revisions in response to Reviewer 1's points.

Comment 1: *The authors should separate their training and testing databases. Ideally, their testing dataset should include a set of previously unseen viruses that have their host experimentally confirmed. In addition, the performance of IPEV should be compared with tools commonly used in the field, including vcontact2: https://doi.org/10.1038/s41587-019-0100-8 and iPHoP: https://doi.org/10.1371/journal.pbio.3002083. However, none of these tools are developed to directly differentiate eukaryotic and prokaryotic viruses, identification of viral taxonomy or host range could lead to the identification of viral type. Moreover, the authors have used multiple approaches for their assessment of the type of viruses. Yet, it is not clear how they combined the results they generated by these approaches in their decisions.*

Response:

We are grateful for Reviewer 1's insightful comments. We would like to address the above comments sequentially, as follows:

(1) About the construction and splitting of the IPEV's data set

Herein, we are sorry that we did not provide a clear statement of the training and test sets used

in our work. We would like to take this opportunity to detail the process of our dataset construction.

We understand Reviewer 1's concern. Defining "unseen viruses" for the purpose of validating a training model remains an open question in the field. In our work, we adopted a strategy different from that of some previous tools that divided their data sets by date, such as VirSorter (Roux et al., PeerJ, 2015), VirSorter2 (Guo et al., Microbiome, 2021), and our previously developed tools like PPR-Meta (Fang et al., Gigascience, 2019), and DeePhage (Wu et al., GigaScience, 2021). **In fact, their temporal separation approaches for training and test sets can inadvertently introduce homologous sequences, which we aimed to avoid in our work.**

Following the approach in our previously developed HoPhage (Tan et al., Bioinformatics, 2021), we first randomly divided the data set into a cross-validation set and an independent test set. Within the independent test set, we constructed a series of data sets with varying degrees of homologous sequences against the training set of IPEV to evaluate the performance of IPEV. The data collection and partitioning strategy was as follows: starting from downloading the taxonomy ID list of viruses and corresponding host lineages from Virus-Host DB (Mihara et al., Viruses, 2016), a manually curated and well-annotated virus host database, as well as genome sequences from the NCBI database (Schoch et al., Database (Oxford), 2020), we first established a 5-fold cross-validation dataset along with an independent test set by randomly dividing the whole database. Subsequently, the data set was randomly split into 5-fold for cross-validation. In each iteration, a one-fold set was used as the validation purpose, while the remaining folds constituted the training set. On the divided training and validation sets, we simulated four groups of sequence lengths: Group A (100-400 bp), Group B (400-800 bp), Group C (800-1,200 bp), and Group D (1,200-1,800 bp). This strategy effectively ensured that the training and validation sets did not overlap. By rotationally repeating this procedure five times, we constructed datasets for 5-fold cross-validation for Groups A to D.

We then created a series of data sets with varying degrees of homology for the independent test set. We specifically ensured that the identity and coverage of sequences in the independent test sets were below a certain threshold z (z < query coverage: x, query identity: y; where x, y = 30%, 60%, 100%) compared to the training set. We generated six low homologies independent test sets (Dataset I1-I6) with varying levels of query coverage and identity relative to the training set, as shown in Tables S5 and S6, as shown below. These steps can be found in Supplementary Materials and Methods. These data sets represent a rigorous collection of low-homology sequences. In the revised

manuscript, we have also included cross-validation with 25,644 eukaryotic sequences from the RVDB database and 5,598 prokaryotic sequences from IMG/VR v4, further strengthening the generalization ability of IPEV.

Therefore, to clarify, we have included the statement "these evaluations are designed to measure IPEV's performance on 'unseen' viruses" in Subsection "Performance on novel viruses with low homology to known databases". (Please refer to Line 240-241, Page 9 in the revised manuscript.)

Table S5. The number of the corresponding prokaryotic virus contigs on Dataset I1-I6.

Dataset	Group A (100-400 bp)	Group B (400-800 bp)	Group C (800-1,200 bp)	Group D (1,200-1,800 bp)
I1	3,180	3,152	3,050	3,171
I2	3,178	3,149	3,050	3,170
I3	4,244	4,124	4,164	4,499
I4	4,242	4,125	4,170	4,499
I5	8,101	8,216	8,411	8,432
I6	8,104	8,216	8,409	8,427

Table S6. The number of the corresponding eukaryotic virus contigs on Dataset I1-I6.

Dataset	Group A (100-400 bp)	Group B (400-800 bp)	Group C (800-1,200 bp)	Group D (1,200-1,800 bp)
I1	1,106	1,117	1,011	1,036
I2	1,106	1,117	1,011	1,036
I3	1,739	1,705	1,785	1,938
I4	1,739	1,703	1,784	1,935
I5	5,356	5,591	6,097	6,069
I6	5,359	5,595	6,082	6,065

In summary, we hope that our responses and changes can adequately address the concerns raised by Reviewer 1 regarding the test set split, particularly ensuring that the test set contains "unseen data" relative to the training set.

(2) Comparisons with vConTACT v.2.0 and iPHoP.

We are grateful to Reviewer 1 for the advice on comparing IPEV with vConTACT v.2.0 (Jang et al., Nat Biotechnol, 2019) and iPHoP (Roux et al., PLoS Biol, 2023). To our best understanding,

vConTACT v.2.0 operates by clustering genes and determining the classification of a given new viral contig by checking for the presence of reference phages within this cluster. On the other hand, iPHoP functions as an automated command-line pipeline designed for predicting the host genus of novel bacteriophages. iPHoP incorporates several tools and databases in its prediction process, including BLASTn for host genome comparison, BLASTn against CRISPR spacer databases, the WIsH (Galiez et al., Bioinformatics, 2017) model, VHM (Ahlgren et al., Nucleic Acids Res, 2017) for similarity analysis, and PHP (Lu et al., BMC Biol, 2021) for host prediction. We therefore began by installing vConTACT v.2.0 and iPHoP according to their manuals and applying them with default parameters to the test sets of the 5-fold cross-validation and independent test sets for Groups A-D. Considering that vConTACT v.2.0 can only accept amino acid sequence inputs from genes, we followed the methods of Wu et al. (Wu et al., GigaScience, 2021) and utilized Prodigal (Hyatt et al., BMC Bioinformatics, 2010) to predict genes in virus contigs and prepared the necessary input files (a FASTA-formatted amino acid file, a "gene-to-genome" mapping file) of vConTACT v.2.0.

However, **we observed that both vConTACT v.2.0 and iPHoP failed to generate effective output.** More specifically, when testing these tools on the test sets of our 5-fold cross-validation and the independent test set (Dataset I1-I6) for Groups A-D, we found that vConTACT v.2.0 was unable to classify the sequences effectively, often identifying them as singletons. On the other hand, while iPHoP functions as a combined pipeline, its RaFAH results were empty. Furthermore, the program generated a warning regarding CRISPR due to the absence of usable hits. **We believe this ineffectiveness of both vConTACT v.2.0 and iPHoP is attributed to the relatively short sequences we designed for our study, with the longest group (Group D) only having a sequence length ranging from 1,200-1,800 bp.**

Therefore, we follow the evaluation methods of Shang et al. (Shang et al., Bioinformatics, 2021). We proceeded to manually simulate test sets of 3,000-5,000 bp lengths (each containing 500 eukaryotic virus genomes and 500 prokaryotic virus genomes) three times based on independent test sets. Using tools including IPEV, HTP, vConTACT v.2.0, and iPHoP, evaluations were performed, and average metrics (Sn, Sp, ACC, Precision, and F1-score) were computed and reported. For a more detailed evaluation, we first utilized Prodigal to predict genes in viral contigs. In our assessment criteria, for each class, positive samples without predictions were counted as false negatives (FN). Conversely, negative samples that lacked prediction results were regarded as true

negatives (TN). In our manuscript, prokaryotic viruses were considered the positive samples. Our findings revealed that IPEV consistently exhibited superior performance, achieving an average F1-score of 0.99, as shown in the newly updated Figure S11. This score significantly surpasses the F1-scores of 0.02 (t = −31.29, P_{adj}<0.0001, two-tailed independent t-tests) and 0.41 (t = −395.63, P_{adj}<0.0001, two-tailed independent t-tests) for vConTACT v.2.0 and iPHoP, respectively. We also observed that under the same configurations and test sets, IPEV operates 50 times faster than HTP, 30 times faster than vConTACT v.2.0, and 1,225 times faster than iPHoP, as shown in the newly updated Figure S2. According to the above findings, we believe that IPEV maintains high accuracy and speed when processing long sequences.

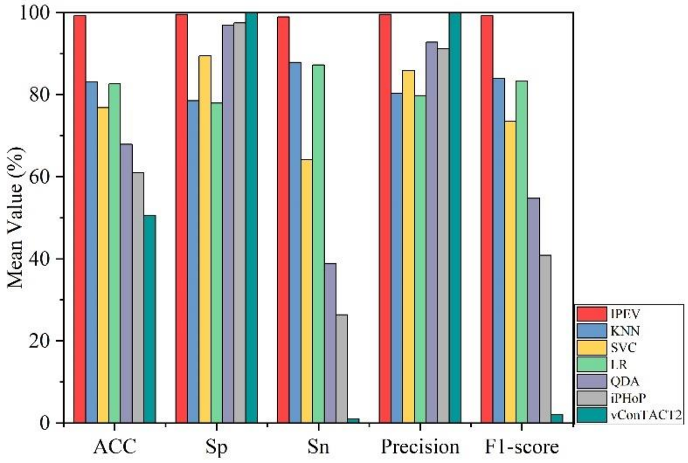

Figure S11. Average performance of IPEV, HTP, iPHoP, and vConTACT v.2.0 across three independent test sets with sequence lengths of 3,000-5,000 bp. (Please refer to Supplementary Materials.)

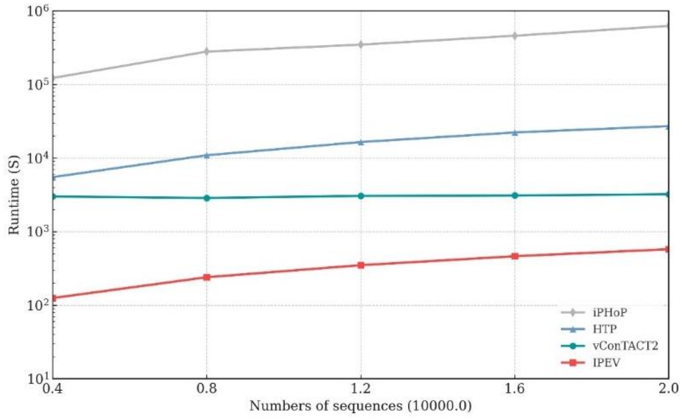

Figure S2. Performance of IPEV, HTP, vConTACT2, and iPHoP as the number of sequences increases under the same computing configuration (1,200-1,800 bp). (Please refer to Supplementary Materials.)

-- In view of the above-mentioned, we first revised "IPEV reduces runtime by 50 times compared to existing methods under the same computing configuration." as "IPEV reduces runtime by at most 1,225 times compared to existing methods under the same computing configuration." in Section "Abstract". (Please refer to Line 26-27, Page 1 in the revised manuscript.)

-- To provide a background for these two tools, we included a brief introduction in the "INTRODUCTION" section in the revised manuscript. We revised "Current computational tools for analyzing virome data focus on classifying the host and lifestyles of phages, such as VirSorter and HoPhage, designed to assign the host for a given phage contig using sequence similarity search or ab initio identification. Other tools, like DeePhage, have been developed to answer questions about the lifestyles of phages" as "Currently, some computational tools for identifying viruses have been introduced in metagenomes, such as HoPhage, iPHoP, WIsH, CHERRY, PHP, and VHM-Net, designed to assign the host for a given phage contig using sequence similarity search or ab initio identification. Some tools, like DeePhage, PHACTS, and PhagePred, have been developed to answer questions about the lifestyles of phages. Other tools, like PPR-Meta, DeepVirFinder, VIBRANT, vConTACT2, VirSorter, and the most recent version, VirSorter2, can be used to identify viruses from metagenomics data". (Please refer to Line 66-72, Page 3 in the revised manuscript.)

-- To maintain consistency in our manuscript, in Section "DISCUSSION", we added the following sentences: "Although IPEV was initially designed for identifying short viral sequences, our study also encompassed an evaluation over extended lengths of 3,000 to 5,000 bp. And this length spectrum allowed us to include vConTACT v.2.0 and iPHoP in our assessment. We constructed three datasets with contig lengths of 3,000-5,000 bp (details on dataset construction and evaluation methods are provided in the Supplementary Materials and Methods). Our findings indicate that IPEV consistently outperformed the others, achieving an average F1-score of 0.99, as depicted in Figure S11. This score significantly exceeds the F1-scores of 0.02 for vConTACT v.2.0 ($t = -31.29$, $P_{adj} < 0.0001$, two-tailed independent t-tests) and 0.41 for iPHoP ($t = -395.63$, $P_{adj} < 0.0001$, two-tailed independent t-tests), respectively." in the revised manuscript (please refer to Line 414-422, Page 15 in the revised manuscript).

-- To make our paper easier to follow, we rearranged the section about processing time, moving the evaluation of runtime to follow the above-discussed results. We revised "As shown in Figure S2,

IPEV can reduce the time required by 50%, taking only 9.6 minutes to analyze 20,000 sequences of 1,200-1,800 bp when using the same computational resources (CPU: Intel(R) Xeon(R), 20 cores, GPU: NVIDIA Corporation GV100GL [Tesla V100 PCIe 32 GB])" as "As shown in Figure S2, IPEV operates 50 times faster than HTP, 30 times faster than vConTACT v.2.0, and 1,225 times faster than iPHoP, taking only 9.6 minutes to analyze 20,000 sequences of 1,200-1,800 bp when using the same computational resources (CPU: Intel(R) Xeon(R), 20 cores, GPU: NVIDIA Corporation GV100GL [Tesla V100 PCIe 32 GB])". (Please refer to Line 428-431, Page 15 in the revised manuscript.)

We added a subsection in Section "Supplementary Materials and Methods" of Section "Supplementary Materials" as:

Construction of extended-length datasets:

In addition to our viral genome fragment evaluations, we extended our assessment of IPEV's performance to include viral genome fragments of longer lengths. Specifically, we focused on test sets ranging from 3,000 to 5,000 base pairs (bp). To achieve a comprehensive evaluation, we manually simulated these sets based on independent test sets, each consisting of an equal number of eukaryotic and prokaryotic virus genomes, totaling 500 contigs from each category. This simulation was repeated three times. This approach was designed to ensure robustness in our results. (Please refer to Supplementary Materials.)

We also added below references into Section "References":

"Roux S, Camargo AP, Coutinho FH et al. iPHoP: An integrated machine learning framework to maximize host prediction for metagenome-derived viruses of archaea and bacteria, PLoS Biol. 2023;21(4):e3002083."

"Bin Jang H, Bolduc B, Zablocki O et al. Taxonomic assignment of uncultivated prokaryotic virus genomes is enabled by gene-sharing networks, Nat Biotechnol. 2019;37(6):632-39."

"Coutinho FH, Zaragoza-Solas A, Lopez-Perez M et al. RaFAH: Host prediction for viruses of Bacteria and Archaea based on protein content, Patterns (N Y). 2021;2(7):100274."

Therefore, we hope that the above responses and revisions have addressed Reviewer 1's concerns regarding the comparison with existing tools. Given that both tools are unable to generate effective outputs for evaluation on particularly short sequences, we have intentionally designed an evaluation over a longer length range.

(3) Assessment of the types of viruses in IPEV

Herein, we apologize for not providing a clear description of the virus type assessment process within IPEV in our initial manuscript. We would like to take this opportunity to clarify:

As previously outlined in the Supplementary Materials, we obtain viral taxon scores from subsequence predictions. When the IPEV model encounters a query viral fragment shorter than 1,800 bp, it utilizes the model from the corresponding length group for prediction. For fragment lengths exceeding 1,800 bp, the sequence is first segmented into 1,800 bp windows, with each segment then assessed using the model from Group D. Any remaining subsequences, whether they measure 0 to 400 bp, 400 to 800 bp, or 800 to 1,200 bp, belong to use model from Groups A, Group B, or Group C, respectively. The overall prediction likelihood score is then calculated as the weighted average of the scores derived from each subsequence's evaluation.

Therefore, to provide clarity, we have included the statement, "When using IPEV, the final viral taxon scores are obtained by weighted averaging of the subsequence predictions. The detailed calculation methodology is outlined in the Supplementary Materials and Methods section" in Section "MATERIALS AND METHODS" (please refer to Line 207-209, Page 8 in the revised manuscript). We thus believe that this added sentence will prevent any further confusion to readers.

Minor comments:

Please use either phageome or phages instead of phage virome. There are some typos in the text that need to be fixed.

Response:

We thank Reviewer 1 for this helpful advice to have a clear expression of our work. In the revised manuscript, we have replaced "phage virome" with "phageome" or "phages".

Furthermore, we have improved the quality of our grammar and spelling, and corrected the following typos in the revised manuscript such as:

-- We have standardized the notation of SN to Sn throughout the manuscript.

-- We have replaced the word "identification" with "differentiation" as "Obviously, these analyses first require the precise differentiation of eukaryotic viruses from prokaryotic viral sequences, and it's beneficial for gaining a comprehensive understanding of the viral landscape."

(Please refer to Line 50-51, Page 2 in the revised manuscript.)

-- We have replaced "base insert or delete" with "insertions or deletions" in Line 284 and 293, in the revised manuscript.

-- We italicize names of viral taxa. All the viral taxa mentioned in the manuscript have been italicized such as *CrAss-like* and *Microviridae* phages in the Line 364 in the revised manuscript.

-- In Line 357, 358, 366, and 369 in the revised manuscript, we revised "phage virome" to "phageome".

Here, we list some of the modifications related to the above-mentioned grammatical and accuracy issues. For additional amendments, please refer to red texts of the main text.

In summary, we are thankful to Reviewer 1 for so many constructive suggestions and comments. In response to the guidance, we have conducted the relevant calculations and expanded our comparative analysis to include a broader array of tools. We have manually curated a new independent test set for a more in-depth evaluation, integrating the tools vConTACT v.2.0 and iPHoP specifically for this objective. Our results demonstrate that IPEV continues to maintain outstanding performance, achieving average F1-score of 0.99, which significantly surpasses the F1-scores of 0.02 and 0.41 for vConTACT v.2.0 and iPHoP, respectively. Besides, performance on running time shows that IPEV operates 50 times faster than HTP, 30 times faster than vConTACT v.2.0, and 1,225 times faster than iPHoP under the same configurations and datasets.

To Reviewer 2:

General Comments: *Yin et al described the development and testing of IPEV, a deep-learning-based model that detects and discriminates sequences derived from prokaryotic and eukaryotic viruses in virome datasets. The model was developed using a set of reference viral sequences with known host information. The sequences were represented as sequence pattern matrices that contained values derived from the frequency and order of trinucleotide pairs. These matrices were subsequently used to train a 2D convolutional neural network that generates a 2-value vector for each input sequence, indicating the probability that the sequence corresponds to a prokaryotic or eukaryotic virus. The model was trained and tested using 5-fold cross validation on the reference*

set, and the authors assessed the robustness of the method using input datasets covering a range of homology and mutation rate values. Finally, the authors applied their model to a gut virome dataset from Shkoporov et al 2019.

Indeed, IPEV represents a novel method that classifies viral sequences based on the type of host they target (prokaryotic or eukaryotic), and the results presented indicate that it efficiently covers a wide range of sequence lengths (from 100 bp). A model like IPEV provides a focus on eukaryotic viruses that is relatively shallow, in comparison with phages for which a wide range of prediction tools have been developed to date. Nevertheless, there are a few points that the authors need to address, particularly in relation to the robustness of the model.

Response:

We first appreciate Reviewer 2's positive comments on our novelty, as "*IPEV represents a novel method that classifies viral sequences based on the type of host they target*". We appreciate Reviewer 2's suggestion about improving the robustness of the model. We also especially thank Reviewer 2 for other comments and suggestions, which were certainly helpful and valuable for us to improve our work. We recognize the importance of these comments and have carefully considered each point in our revisions. Our responses to Reviewer 2's specific concerns and the subsequent revisions to our manuscript are detailed below.

Comment 1: *I am concerned about the number of reference sequences that were employed to train the model, and it makes me question its general applicability to viromes from any kind of biome. It would be great if the authors incorporated more sequences to their training and validation. Sources of viral sequences such as IMG/VR (https://img.jgi.doe.gov/cgi-bin/vr/main.cgi) and RVDB (https://rvdb.dbi.udel.edu/) could be useful for identifying further sequences, and generate a set that cover a much wider range of viral diversity. Perhaps, this could also lead to an improved performance for the gut datasets.*

Response:

Herein, we should apologize that the initially provided datasets were not comprehensive enough, which led to Reviewer 2's concerns regarding the general applicability of our tool. We are also grateful to Reviewer 2 for suggesting two additional data sets, which have helped us enhance the generalizability of our tool.

After consultation with Dr. Antonio, the lead author of IMG/VR v4 (Camargo et al., Nucleic

Acids Res, 2023), we learned that the database chiefly contains annotations for viruses that target bacteria and archaea. Recently, we accessed IMG/VR v4 through the following link: https://genome.jgi.doe.gov/portal/pages/dynamicOrganismDownload.jsf?organism=IMG_VR. We proceeded to download the high-confidence nucleotide database (IMGVR_all_nucleotides-high_confidence.fna). The "high-confidence" designation refers to the estimated accuracy regarding the completeness and purity of single-scaffold viral genomes, as assessed by CheckV software, a tool utilized by the creators of IMG/VR v4 for qualification purposes. To cluster the sequences, we applied the cd-hit utility, setting the similarity threshold at 100%. The precise command executed was: cd-hit -i $INPUT_FILE -o $OUTPUT_FILE_100 -c 1.0 -n 10 -M 40000 -T 40. Following this, we conducted a filtering process on the IMGVR_UViG database, selectively retaining rows where the "Host prediction method" included references to "CRISPR" and "Isolate taxonomy". Considering that our tool's scope does not encompass proviruses, we excluded them and selected only the high-quality sequences. From these procedures, we get 5,598 prokaryotic sequences.

Secondly, following the recommendation, we incorporated data from the Reference Viral Database (RVDB) (Goodacre et al., mSphere, 2018). We therefore accessed RVDB on September 15, 2023, via the website https://rvdb.dbi.udel.edu/ and procured the clustered version of the database v26.0 (released on April 10, 2023), named "C-RVDBv26.0.fasta". We then clustered the sequences in C-RVDB using cd-hit with the command: cd-hit -i $INPUT_FILE -o $OUTPUT_FILE_100 -c 1.0 -n 10 -M 40000 -T 40. We excluded unverified, partial sequences, and refseq entries since we already have the latter in our previous collection. For each gene, we chose to retain only one strain variant, setting a threshold of 30 for each virus. Through these methods, we obtained 25,644 eukaryotic sequences. After obtaining the data mentioned above, we used MetaSim to simulate Groups A through D, with quantities as shown in Table R1.

Table R1. Number of simulated contigs newly added based on RVDB and IMG/VR v4 databases.

Group	Number of prokaryotic virus contigs newly added	Numbers of eukaryotic virus contigs newly added
Group A (100-400 bp)	500,000	500,000
Group B (400-800 bp)	450,000	450,000

Group C (800-1,200 bp)	400,000	400,000
Group D (1,200-1,800 bp)	350,000	350,000

In light of the viral genomes in the RVDB that include segments of uncertain non-viral origin, and considering that IMG/VR v4 primarily employs computational methods for host identification, it stands in contrast to the initial construction of IPEV, which was based on manually curated reference genomes. Therefore, we have decided to integrate datasets from IMG/VR v4 and RVDB solely into the training set for 5-fold cross-validation, aiming to enhance the generalizability of our model. In response to the increased dataset volume, we have accordingly increased our neural network's capacity by adding to the number and size of the filters, which increase the parameter count from 1,152,898 to 2,906,690. And we set the learning rate of the neural network as 0.0005 to optimize the loss function better. More specifically, the original dropout rate of 0.30 and a convolutional layer with 64 filters of size 5×5 have been adjusted to a dropout rate of 0.32 and a convolutional layer with 128 filters of size 7×7. Furthermore, we have refined our training process by reducing the batch size to 16 from the previous 32, allowing for more robust weight updates during training.

Following the above adjustments, we re-initiated the 5-fold cross-validation on Groups A (100-400 bp) - Group D (1,200-1,800 bp), re-evaluated IPEV and related tools on independent test sets with different levels of homology from the training set of IPEV, datasets with sequencing errors, datasets with functional proteins, real virome data, and re-evaluated the stability of the gut virome using the most recent version of IPEV. We observed that these changes resulted in an improvement in our tool's performance. Therefore, we have updated Figure 2 in the main manuscript accordingly, as shown in the newly updated Figure 2. Specifically, in the 5-fold cross-validation for Group A, there was an average increase of 0.1% in accuracy (ACC), specificity (Sp), and precision, while sensitivity (Sn) improved by 0.8% and the F1-score by 0.2%. For Group B, there was a 0.3% enhancement in both ACC and F1-score, and a 0.5% increase in Sn. Precision in Group C showed an increase of 0.3%. In Group D, ACC improved by 0.3%, Sn and Sp both by 0.6%, and the F1-score by 0.3%.

Besides, we observed about a 1% improvement in the independent test set (Dataset I1-I6), on

different metrics. Furthermore, in the analysis of the gut virome, the average AUC score improved by 0.3%. Additionally, our findings regarding the stability of the gut virome remain unchanged. We deeply appreciate the reviewers' insights and suggestions.

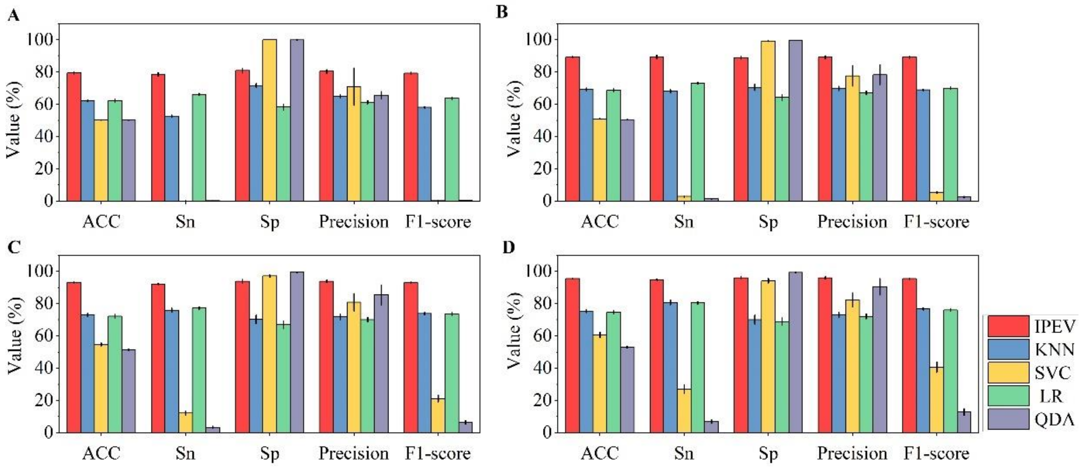

Figure 2. Panels A, B, C, and D display the comparative performance of IPEV and HTP (KNN, SVC, LR, and QDA) with 5-fold cross-validation across Groups A, B, C, and D, respectively. * Sn = TP / (TP + FN), Sp = TN / (TN + FP), ACC = (TP + TN) / (TP + TN + FP + FN), Precision = TP / (TP + FP), F1-score = 2 × Precision × Recall / (Precision + Recall), where TP, TN, FP, and FN respectively represent true positive, true negative, false positive, and false negative. As the method with the best performance in HTP, KNN is selected for comparison. The mean and standard deviation of 5-fold cross-validation are computed to elaborate on performance evaluation. Due to a lack of reconstruction between the train and validation set, the performance of HTP (KNN) is overestimated. (In this paper, prokaryotic viruses are treated as positive samples.)

-- To make our paper easier to follow, in Subsection "Dataset construction" of Section "MATERIALS AND METHODS" in the revised manuscript, we revised the sentences as: "Without accurately host-annotated virome datasets that could serve as benchmarks, we generated simulated datasets based on well-annotated complete virus genomes. Firstly, we downloaded the taxonomy ID list of viruses and corresponding host lineages from the Virus-Host DB and genome sequences from the NCBI database on Oct. 31, 2021. As a result, we established our first dataset, referred to as Dataset-1, which contains 11,022 eukaryotic virus genomes and 5,051 prokaryotic virus genomes (of which 113 are attributed to archaeal viruses). To enhance the model's generalizability, we incorporated additional data from the Reference Viral Database (RVDB) and 25,644 eukaryotic sequences, along with 5,598 prokaryotic sequences from IMG/VR v4, collectively termed Dataset-2. Details regarding the data inclusion criteria are outlined in the Supplementary Materials and Methods. Dataset-1 is sourced from reference sequences and manually curated with credible host annotations, while Dataset-2 is not. Therefore, based on genomes, we used all viruses in Dataset-2

and 10,000 eukaryotic and 4,000 prokaryotic viruses in Dataset-1 randomly divided for 5-fold cross-validation, while the remaining subset served as an independent test set for assessing generalizability." (Please refer to Line 102-114, Page 4 in the revised manuscript.)

-- We added the sentences "the CNN has ten layers: two convolution layers (with a 7×7 kernel and the same padding), two max pooling layers (with a 2×2 filter), two dropout layers, a flatten layer and two fully connected layers followed by a softmax activation function." into Subsection "Structure of the deep learning neural network" in Section "MATERIALS AND METHODS". (Please refer to Line 169-171, Page 6 in the revised manuscript.)

-- We revised the sentences as "We employed the Adam optimizer (learning rate = 0.0005) and batch size 16 to train the neural network and update network weights (F = 128, M = 2, S1 = S2 = 2, P = 0.32, and R = 64). The architecture of the IPEV neural network is depicted in Figure 1C. When using IPEV, the final viral taxon scores are obtained by weighted averaging of the subsequence predictions. The detailed calculation methodology is outlined in the Supplementary Materials and Methods section" (Please refer to Line 205-209, Page 7 in the revised manuscript.)

-- We revised "In contrast, our IPEV tool demonstrated rather better performance under the same conditions, with Sn of 77.6% and Sp of 80.8%." as "In contrast, our IPEV tool demonstrated rather better performance under the same conditions, with Sn of 78.4% and Sp of 80.9%." (Please refer to Line 223-225, Page 8 in the revised manuscript.)

-- We revised "Conversely, under the same conditions, the IPEV tool exhibited superior performance, with Sn of 95.0% and Sp of 95.3%." as "Conversely, under the same conditions, the IPEV tool exhibited superior performance, with Sn of 95.0% and Sp of 96.1%." (Please refer to Line 232, Page 8 in the revised manuscript.)

-- We revised "Specifically, in Group A (100-400 bp) of Dataset I1, IPEV reported Sn of 76.22% and Sp of 79.78%." as "Specifically, in Group A (100-400 bp) of Dataset I1, IPEV reported Sn of 76.94% and Sp of 81.19%." (Please refer to Line 249, Page 9 in the revised manuscript.)

-- We revised "the F1-score of IPEV improved from 66.05% to 89.14%." as "the F1-score of IPEV improved from 66.61% to 89.44%." (Please refer to Line 254, Page 9 in the revised manuscript.)

-- We added below references into Section "References":

"Goodacre N, Aljanahi A, Nandakumar S et al. A Reference Viral Database (RVDB) To

Enhance Bioinformatics Analysis of High-Throughput Sequencing for Novel Virus Detection, mSphere. 2018;3(2).”

“Camargo AP, Nayfach S, Chen IA et al. IMG/VR v4: an expanded database of uncultivated virus genomes within a framework of extensive functional, taxonomic, and ecological metadata, Nucleic Acids Res. 2023;51(D1):D733-D43.”

We observed approximately a 1% improvement in I1-I6 of the independent test set across various metrics. The corresponding updates have been made in the tables of the Supplementary Materials, which we will not repeat here in brief.

In the Supplementary Materials, we add the Table S2 as follows:

Table S2. Number of simulated contigs based on RVDB and IMG/VR v4 databases. (Please refer to Supplementary Materials)

Group	Number of prokaryotic virus contigs	Numbers of eukaryotic virus contigs
Group A (100-400 bp)	500,000	500,000
Group B (400-800 bp)	450,000	450,000
Group C (800-1,200 bp)	400,000	400,000
Group D (1,200-1,800 bp)	350,000	350,000

We added a subsection in Section “Supplementary Materials and Methods” of Section “Supplementary Materials” as:

Construction of Dataset-2 from RVDB and IMG/VR:

We accessed IMG/VR v4 on September 15, 2023, through the following link: https://genome.jgi.doe.gov/portal/pages/dynamicOrganismDownload.jsf?organism=IMG_VR. We proceeded to download the high-confidence nucleotide database (IMGVR_all_nucleotides-high_confidence.fna). The “high-confidence” designation refers to the estimated accuracy regarding the completeness and purity of single-scaffold viral genomes, as assessed by CheckV software—a tool utilized by the creators of IMG/VR v4 for qualification purposes. To cluster the sequences, we applied the cd-hit utility, setting the similarity threshold at 100%. The precise command executed was: cd-hit -i $INPUT_FILE -o $OUTPUT_FILE_100 -c 1.0 -n 10 -M 40000 -T 40. Following this,

we conducted a filtering process on the IMGVR_UViG database, selectively retaining rows where the "Host prediction method" included references to "CRISPR" and "Isolate taxonomy". Considering that our tool's scope does not encompass proviruses, we excluded them and selected only the high-quality sequences. From these procedures, we get 5,598 prokaryotic sequences.

Secondly, we incorporated data from the Reference Viral Database (RVDB) (Goodacre et al., mSphere, 2018). We accessed RVDB on September 15, 2023, via the website https://rvdb.dbi.udel.edu/ and procured the clustered version of the database v26.0 (released on April 10, 2023), named "C-RVDBv26.0.fasta". We then clustered the sequences in C-RVDB using cd-hit with the command: cd-hit -i $INPUT_FILE -o $OUTPUT_FILE_100 -c 1.0 -n 10 -M 40000 -T 40. We excluded UNVERIFIED, partial sequences, and REFSEQ entries since we already have the latter in our collection. For each gene, we chose to retain only one strain variant, setting a threshold of 30 for each virus. Through these methods, we obtained 25,644 eukaryotic sequences. (Please refer to Supplementary Materials.)

Comment 2: *Even though viral enrichment methods increase the concentration of viral DNA, the presence of contaminant DNA from other microbes in the enriched viral samples is common. Currently, the results do not indicate what the performance of the model would be in the presence of contaminating sequences. I suggest the authors to carry out tests that demonstrate the performance of IPEV when analysing a sample containing microbial contamination (ideally from both prokaryotes and eukaryotes) and demonstrate that IPEV is not prone to wrongly reporting these sequences as viruses.*

Response:

We are thankful for Reviewer 2's suggestions and apologize for not having adequately detailed our approach to virome samples with contamination in our manuscript. Actually, several previous studies have indicated that bacterial and fungal contamination is a prevalent issue in virome datasets (Zhang et al., Nature communications, 2021; Zolfo et al., Nature biotechnology, 2019). To address this issue, in this revision we have developed an additional algorithm that distinguishes between viral and non-viral components (bacteria and fungi) using the sequence pattern matrix and a neural network framework within IPEV. This model currently serves as an optional module in our IPEV program, which users may choose to enable or disable as required. The following outlines the

construction details for our decontamination model:

We first designed a comprehensive set, creating 2,000,000 sequences for Group A (100-400 bp), 3,600,000 for Group B (400-800 bp), 3,200,000 for Group C (800-1,200 bp), and 2,800,000 for Group D (1,200-1,800 bp), maintaining a 1:1 ratio of viral to non-viral contigs.

For construction of the non-viral component data set, we performed searches on the NCBI Assembly database using the commands "(Fungi[orgn]) AND (representative genome[filter])" and "(Bacteria[orgn]) AND (representative genome[filter])" to download data sets. We thus downloaded 1,428 bacterial genome sequences, ensuring to exclude plasmids and include only those labeled as "complete", and 99 fungal sequences, excluding macrofungi. We then simulated contigs to create negative sample groups: 2 million for Group A (100-400 bp), 1,800,000 for Group B (400-800 bp), 1,600,000 for Group C (800-1,200 bp), and 1,400,000 for Group D (1,200-1,800 bp). Based on the sequence pattern matrix and neural network framework outlined in our manuscript, we retrained, validated, and tested on the constructed Groups A to D using an 8:1:1 split for training, validation, and testing, respectively. Furthermore, we conducted two separate experiments where the non-viral components constituted 50% of the test set.

In this experiment, we made sample sets with an equal number of virus-like and non-virus-like organisms (bacteria and fungi) in Group A to D. We then calculated Sn and Sp for each group. As shown in the newly added Figure S12, for viral identification, Sn values were found to be 73.1% for Group A, 83.3% for Group B, 90.5% for Group C, and 93.1% for Group D. Correspondingly, for non-viral identification, Sp values were 84.5% for Group A, 93.7% for Group B, 95.7% for Group C, and 97.3% for Group D. This enhanced functionality, designed to reduce false-positive (non-viral) samples, has been integrated into the IPEV tool, complete with a user-activated switch. We are grateful for Reviewer 2's invaluable advice.

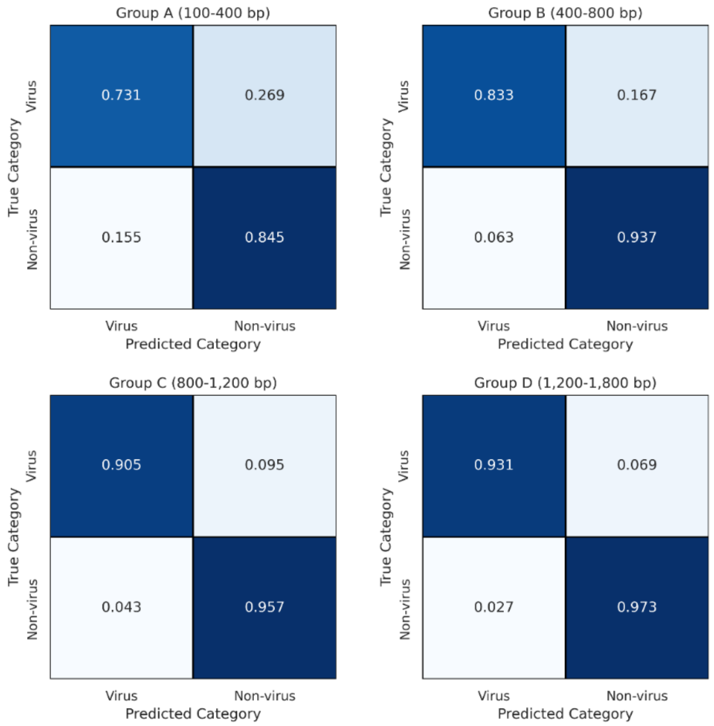

Figure S12. the confusion matrices show the false positive reduction capability of IPEV on datasets with a 1:1 ratio of viruses to non-viruses across Groups A to D. (Please refer to Supplementary Materials)

Given the focus of our paper on distinguishing between prokaryotic and eukaryotic viruses within virome data, to make our paper easier to follow, we have added these experiment results into Supplementary Materials. In Section "DISCUSSION" in the revised manuscript, we addressed the issue of contamination in viromes and introduce our functionality that tackles this problem.

-- In Section "DISCUSSION" in the revised manuscript, we added the sentences as "An important consideration when using IPEV is the potential bacterial and fungal contamination in virome datasets. To address this, we designed a feature within IPEV that eliminates false-positive non-viral components (bacteria and fungi). Our results show IPEV's effectiveness in differentiating viral from non-viral genome fragments in datasets where viruses and non-viruses are present in a 50:50 ratio. As shown in Figure S12, across Groups A to D, IPEV achieved Sn scores of 0.73, 0.833, 0.905, and 0.931, respectively (details on sample construction and methods can be found in Supplementary Materials and Methods). This feature is available as an optional switch." (Please refer to Line 437-443, Page 16 in the revised manuscript.)

-- In Section "Supplementary Materials and Methods" in Supplementary Materials, we added a subsection as:

Construction of viral and non-viral datasets

We designed a comprehensive dataset, creating 4,000,000 sequences for Group A (100-400 bp), 3,600,000 for Group B (400-800 bp), 3,200,000 for Group C (800-1,200 bp), and 2,800,000 for Group D (1,200-1,800 bp), maintaining a 1:1 ratio of viral to non-viral contigs. For the construction of the non-viral component dataset, we performed searches on the NCBI Assembly database using the commands "(Fungi[orgn]) AND (representative genome[filter])" and "(Bacteria[orgn]) AND (representative genome[filter])" to download data sets. We downloaded 1,428 bacterial genome sequences, ensuring to exclude plasmids and include only those labeled as "complete", and 99 fungal sequences, excluding macrofungi. We simulated contigs to create negative sample groups: 2 million for Group A (100-400 bp), 1,800,000 for Group B (400-800 bp), 1,600,000 for Group C (800-1,200 bp), and 1,400,000 for Group D (1,200-1,800 bp). Based on the sequence pattern matrix and neural network framework outlined in our manuscript, we retrained, validated, and tested on the constructed Groups A to D using an 8:1:1 split for training, validation, and testing, respectively. Furthermore, we conducted experiments where the non-viral components constituted 50% of the test set. Viruses are treated as the positive sample.

-- In addition, we added below references into Section "References":

"Zhang F, Zuo T, Yeoh YK et al. Longitudinal dynamics of gut bacteriome, mycobiome and virome after fecal microbiota transplantation in graft-versus-host disease, Nat. Commun. 2021;12(1):65."

"Zolfo M, Pinto F, Asnicar F et al. Detecting contamination in viromes using ViromeQC, Nat. Biotechnol. 2019;37(12):1408-12."

Comment 3: *I find the results of the gut samples interesting and appropriate for the scope of IPEV. However, if IPEV is meant to be a general-purpose tool for virome analysis, it would be ideal if the authors provided results demonstrating the performance of the tool with samples from other biomes. For example, the authors could analyse datasets from the TARA Oceans project (e.g., 10.1016/j.cell. 2019.03.040), some of which have already been assembled (https://www.ebi.ac.uk/ena/browser/ view/PRJEB22493).*

Response:

We are grateful and appreciative that Reviewer 2 finds that "*the results of the gut samples*

interesting and appropriate for the scope of IPEV". Moreover, we are thankful to Reviewer 2's for the helpful advice. Following this suggestion, we downloaded a dataset comprising 243 marine virome samples from the European Nucleotide Archive (accession number: PRJEB22493) (Rangel-Pineros et al., PLOS Computational Biology, 2023), which is part of the Global Ocean Viromes 2.0 (GOV 2.0) dataset (Gregory et al., Cell, 2019), with a combined size of approximately 166 GB, to evaluate the efficacy of IPEV alongside comparable tools. Initially, we used BLASTn against reference viral genomes with an e-value threshold of e-5 for annotation, which allowed us to assign genomes with eukaryotic and prokaryotic virus labels. Subsequently, we employed IPEV and HTP to score and assess the annotated prokaryotic and eukaryotic viral sequences in each sample.

On these 243 marine virome samples, we calculated the AUC scores for each tool individually. We reported the overall average and median AUC for these samples, and we noted that our tool, IPEV, outperforms HTP (KNN, and others) across all samples. In our comprehensive evaluation, IPEV demonstrated its advanced capabilities by consistently outperforming other tools with its higher average AUC values: it outperforms SVC by 0.18, exceeds QDA by 0.20, surpasses LR by 0.16, and betters KNN by 0.19. These results indicate that IPEV is a highly competitive tool. We observed that in marine viromes the performance of IPEV was not as high as in the test set. Consequently, we analyzed the sequence length proportions in these 243 samples. We found that nearly every sample contained more than 60% of sequences shorter than 1,000 bp. This prevalence of short sequences might be the reason for the reduced performance of IPEV.

-- In Section "INTRODUCTION", we revised the sentences as "Virus identification typically involves aligning sequences against known viruses in genomic repositories, such as the National Center for Biotechnology Information (NCBI) Taxonomy Databases and European Nucleotide Archive (ENA)". (Please refer to Line 61, Page 3 in the revised manuscript.)

-- In Section "MATERIALS AND METHODS" in the revised manuscript, we added the sentence "We also use real virome to evaluate IPEV and related tools. We first downloaded a dataset comprising 243 marine virome samples from the ENA (accession number: PRJEB22493)". (Please refer to Line 136-137, Page 5 in the revised manuscript.)

-- In Section "RESULT" in the revised manuscript, we added a subsection as follows:

Performance on the marine virome

We collected data from 243 marine virome samples with assembled contigs and annotated them for

sequence type using Blastn. We evaluated our tool, IPEV, along with other related tools. We reported the overall average and median AUC for the samples, and we noted that our tool, IPEV, outperforms HTP (KNN, and others) across all samples. As shown in Figure 7, in our comprehensive evaluation, IPEV demonstrated its advanced capabilities by consistently outperforming other tools with its higher average AUC values: it outperforms SVC by 0.18, exceeds QDA by 0.20, surpasses LR by 0.16, and betters KNN by 0.19. These results indicate that IPEV is a highly competitive tool. (Please refer to Line 317-324, Page 11 in the revised manuscript.)

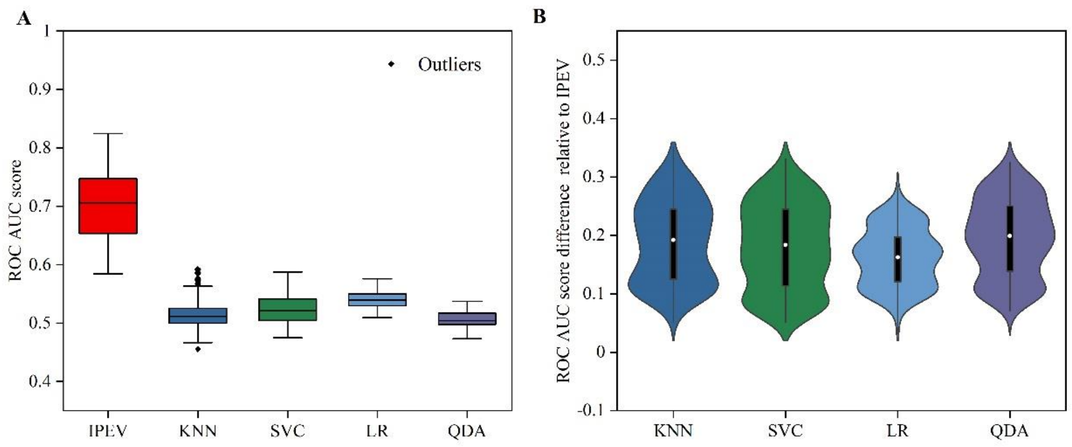

Figure 7. A. Box plots representing the AUC scores of the ROC curves for IPEV, KNN, SVC, LR, and QDA. **B.** Violin plots displaying the AUC scores differences of each tool relative to IPEV.

-- In addition, we added below references into Section "References":

"Yuan D, Ahamed A, Burgin J et al. The European Nucleotide Archive in 2023, Nucleic Acids Res. 2023; gkad1067."

"Rangel-Pineros G, Almeida A, Beracochea M et al. VIRify: an integrated detection, annotation and taxonomic classification pipeline using virus-specific protein profile hidden Markov models, PLoS Comput. Biol. 2023;19(8):e1011422."

"Gregory AC, Zayed AA, Conceição-Neto N et al. Marine DNA viral macro-and microdiversity from pole to pole, Cell. 2019;177(5):1109-23. e14."

Comment 4: *There are several instances in the manuscript where the authors indicate the existence of significant differences between metrics measured to compare the performance of tools (e.g., line 326: "which was significantly higher than the mean AUC values of …"), but there is no mention of*

statistical analyses conducted to reach those conclusions (except for the Wilcoxon rank-sum test in line 305). Please provide information on statistical tests conducted to identify the significant differences.

Response:

Herein we agree with Reviewer 2 that we should provide information on statistical tests to identify the significant differences. We apologize for this oversight and have now recalculated all instances where significance was mentioned, performing the appropriate hypothesis testing.

-- We thus revised "Our analysis, as illustrated in Figure 8A, demonstrated that IPEV exhibited higher accuracy than HTP in more than 90% of the real virus samples and outperformed the other four models (KNN, SVC, LR, and QDA), with a mean AUC value of 0.64, which was significantly higher than the mean AUC values of 0.55, 0.51, 0.54, and 0.51 for KNN, SVC, LR, and QDA, respectively" as "Our analysis, illustrated in Figure 8A, demonstrated that IPEV exhibited higher accuracy than HTP in over 90% of the real virus samples. Furthermore, IPEV's mean AUC value of 0.64 was significantly superior to those of KNN (0.55), SVC (0.51), LR (0.54), and QDA (0.51), according to the Wilcoxon rank-sum test results using Benjamini-Hochberg adjustment (IPEV with KNN: $p < 2.89e-19$; IPEV with SVC: $p < 5.08e-34$; IPEV with LR: $p < 5.23e-22$; IPEV with QDA: $p < 3.79e-36$)". (Please refer to Line 334-339, Page 12-13 in the revised manuscript.)

-- We revised "The results showed that the average coefficient of variation of the phagevirome was significantly lower (0.04 ± 0.01) compared to the PPV (0.58 ± 0.05), indicating a high degree of temporal stability of the phagevirome as illustrated in Figure 8C." as "The results showed that the average coefficient of variation of the phageome was significantly lower (0.04 ± 0.01) compared to the PPV (0.58 ± 0.05), indicating a high degree of temporal stability of the phageome, as illustrated in Figure 8C and Table S15." (Please refer to Line 358, Page 13 in the revised manuscript.)

Table S15: P-values show the phageome's average coefficient of variation was significantly lower compared to the PPV (Wilcoxon rank-sum test, adjusted using the Benjamini-Hochberg correction). (Please refer to Supplementary Materials)

Subject	Unadjusted P-Value	Adjusted P-Value
Subject 916	0.000674	0.002245
Subject 917	0.004389	0.006270
Subject 918	0.000674	0.002245

Subject 919	0.043208	0.043208
Subject 920	0.001522	0.003806
Subject 921	0.002080	0.004161
Subject 922	0.004389	0.006270
Subject 923	0.010080	0.012600
Subject 924	0.014882	0.016536
Subject 925	0.000674	0.002245

-- In addition, we added a paragraph as "Although IPEV was initially designed for identifying short viral sequences, our study also encompassed an evaluation over extended lengths of 3,000 to 5,000 bp. And this length spectrum allowed us to include vConTACT v.2.0 and iPHoP in our assessment. We constructed three datasets with contig lengths of 3,000–5,000 bp (details on dataset construction and evaluation methods are provided in the Supplementary Materials and Methods). Our findings indicate that IPEV consistently outperformed the others, achieving an average F1-score of 0.99 as depicted in Figure S11. This score significantly exceeds the F1-scores of 0.02 for vConTACT v.2.0 ($t = -31.29$, $P_{adj} < 0.0001$, two-tailed independent t-tests using Benjamini-Hochberg adjustment) and 0.41 for iPHoP ($t = -395.63$, $P_{adj} < 0.0001$, two-tailed independent t-tests), respectively." in Section "DISCUSSION". (Please refer to Line 414-422, Page 15 in the revised manuscript.)

In summary, following above recommendations raised by Reviewer 2, we have increased additional datasets in our cross-validation dataset and re-train our neural network accordingly. After recalculating all results mentioned in the manuscript, we observed that these changes contributed positively to the model's performance, especially evident in the cross-validation datasets and the gut virome datasets. Our initial conclusions about the stability of the gut virome remain valid. Furthermore, we have demonstrated the applicability of our model to other viromes by evaluating it using a marine virome dataset consisting of 243 samples, where IPEV showed as the top-performing tool, particularly when utilizing BLAST for annotations. In addition to these improvements, we have developed a optional module that can remove contamination from the gut virome. It demonstrated that this module allows for accurate discrimination between prokaryotic and eukaryotic viruses in sequences with microbial contamination by eliminating the contaminated sequences. Overall, we are so thankful to Reviewer 2 for so many constructive suggestions and comments.

Minor comments:

Minor comment 1: *There is a reference missing in line 37.*

Response:

We are sorry for missing a reference in line 37. We have cited the reference (Mushegian AR. Are There 10(31) Virus Particles on Earth, or More, or Fewer? J Bacteriol. 2020) and have rearranged the order of cited reference in the revised manuscript.

Minor comment 2: *In the sentence between lines 41-44, it is not clear what you are referring to with "identification of viral sequences". Are you referring to viral vs non-viral, or to host identification?*

Response:

We are sorry for the misunderstanding expression. Actually, we are referring to issues with host identification. To make it clear, we revised "Nonetheless, it is essential to note that enriched sample approaches carry the risk of losing valuable host or environmental information, caused by inaccurate identification of viral sequences, thereby constraining subsequent analyses" as "Nonetheless, it is essential to note that enriched sample approaches carry the risk of losing valuable host or environmental information, and can cause inaccurate virus host identification, thereby constraining subsequent analyses." (Please refer to Line 42-44, Page 2 in the revised manuscript.)

Minor comment 3: *Line 50: you mean "identification" or "differentiation"?*

Response:

Thank Reviewer 2 for pointing out the ambiguity. we have changed the word "*identification*" with "*differentiation*", we are thankful for Reviewer 2's suggestion. (Please refer to Line 49-51, Page 2 in the revised manuscript.)

Minor comment 4: *The two sentences between lines 49 – 52 seem redundant. I would suggest rewriting these into a single sentence.*

Response:

We are grateful for Reviewer 2's advice. To make the expression brief, we replaced "Obviously, these analyses first require the precise identification of phage sequences from eukaryotic viruses.

Therefore, distinguishing between prokaryotic and eukaryotic viruses within virome data is essential for gaining a comprehensive understanding of the viral landscape" as "Obviously, these analyses first require the precise differentiation of eukaryotic viruses from prokaryotic viral sequences, and it's beneficial for gaining a comprehensive understanding of the viral landscape." (Please refer to Line 49-51, Page 2 in the revised manuscript.)

Minor comment 5: *Line 65: the latest version of ICTV taxonomy has 11,273 species. Please update this number.*

Response:

We are thankful to Reviewer 2 for reminding us to update the ICVT taxonomy information, which is currently the number of 11,273. We have revised the sentence as follows: "For instance, it has been estimated that there are millions of viral species, but the International Committee on Taxonomy of Viruses (ICTV) has recognized only 11,273 species to date." (Please refer to Line 63-65, Page 3 in the revised manuscript.)

Minor comment 6: *Line 67: there is a newer version of VirSorter (VirSorter2), which has an expended scope in comparison with the older version. Please, modify the text to include the most up-to-date version of this tool.*

Response:

Herein we are grateful for Reviewer 2's suggestion. We have added the sentence as following in Line 71-72 as "… and the most recent version, VirSorter2, can be used to identify viruses from metagenomics data." (Please refer to Line 66-72, Page 3 in the revised manuscript.) In addition, we added the reference into Section "Reference": "Guo J, Bolduc B, Zayed AA et al. VirSorter2: a multi-classifier, expert-guided approach to detect diverse DNA and RNA viruses, Microbiome. 2021;9(1):37."

Minor comment 7: *There are some more tools with a varied range of strategies for viral prediction that are widely known among the community, which I feel should be mentioned in the introduction (e.g., VIBRANT, DeepVirFinder, PPR-Meta, etc). Even though none of these were explicitly designed for prediction of eukaryotic viruses, it'd be worth commenting on them.*

Response:

We are grateful for Reviewer 2's advice. We therefore added the comment of these tools as: "Currently, some computational tools for identifying viruses have been introduced in metagenomes, such as HoPhage, iPHoP, WIsH, CHERRY, PHP, and VHM-Net, designed to assign the host for a given phage contig using sequence similarity search or ab initio identification. Some tools, like DeePhage, PHACTS, and PhagePred, have been developed to answer questions about the lifestyles of phages. Other tools, like PPR-Meta, DeepVirFinder, VIBRANT, vConTACT2, VirSorter,and the most recent version, VirSorter2, can be used to identify viruses from metagenomics data." in Section "INTRODUCTION" (please refer to Line 66-72, Page 3 in the revised manuscript). In addition, we added the references into Section "References":

"Roux S, Camargo AP, Coutinho FH et al. iPHoP: An integrated machine learning framework to maximize host prediction for metagenome-derived viruses of archaea and bacteria, *PLoS Biol.* 2023;21(4):e3002083."

"Coutinho FH, Zaragoza-Solas A, Lopez-Perez M et al. RaFAH: Host prediction for viruses of Bacteria and Archaea based on protein content, *Patterns (N Y).* 2021;2(7):100274."

"Galiez C, Siebert M, Enault F et al. WIsH: who is the host? Predicting prokaryotic hosts from metagenomic phage contigs, *Bioinformatics.* 2017;33(19):3113-14."

"Shang J, Sun Y. CHERRY: a Computational metHod for accuratE pRediction of virus–pRokarYotic interactions using a graph encoder–decoder model, *Briefings Bioinf.* 2022; *23*(5), bbac182."

"Lu C, Zhang Z, Cai Z et al. Prokaryotic virus host predictor: a Gaussian model for host prediction of prokaryotic viruses in metagenomics, *BMC Biology.* 2021;19(1):5."

"Wang W, Ren J, Tang K et al. A network-based integrated framework for predicting virus–prokaryote interactions, *NAR: Genomics Bioinf.* 2020;2(2)."

"Ren J, Song K, Deng C et al. Identifying viruses from metagenomic data using deep learning, Quant Biol. 2020;8(1):64-77."

"Kieft K, Zhou Z, Anantharaman K. VIBRANT: automated recovery, annotation and curation of microbial viruses, and evaluation of viral community function from genomic sequences, Microbiome. 2020;8(1):90."

"Bin Jang H, Bolduc B, Zablocki O et al. Taxonomic assignment of uncultivated prokaryotic virus genomes is enabled by gene-sharing networks, Nat Biotechnol. 2019;37(6):632-39."

"Roux S, Enault F, Hurwitz BL et al. VirSorter: mining viral signal from microbial genomic data, PeerJ. 2015;3:e985."

"Guo J, Bolduc B, Zayed AA et al. VirSorter2: a multi-classifier, expert-guided approach to detect diverse DNA and RNA viruses, Microbiome. 2021;9(1):37."

Minor comment 8: *Indicate the version of Virus-Host DB used, and the version or date when the viral data was retrieved from NCBI.*

Response:

We are grateful to Reviewer 2 for reminding us to add the version of the database we used. In the revised manuscript, we have indicated the date as: "Firstly, we downloaded the taxonomy ID list of viruses and corresponding host lineages from Virus-Host DB and genome sequences from the NCBI database on Oct. 31, 2021." in Subsection "Dataset construction" (please refer to Line 103-105, Page 4 in the revised manuscript).

Minor comment 9: *Line 124: do you mean 10 samples or 10 adults? If it's the latter, please correct the sentence.*

Response:

We are sorry for this wrong expression. Actually, the dataset included 130 virome samples from ten healthy adults. Therefore, we have revised the sentence as "This dataset included 130 virome samples from ten healthy adults (subjects 916-925) collected over 12 months (T1-T12) through monthly synchronous samplings." (Please refer to Line 140-141, Page 5 in the revised manuscript.)

Minor comment 10: *Line 130: by "genome sequences" are you referring to the assembled viral contigs? In that case, please clarify as it is currently ambiguous.*

Response:

We thank Reviewer 2 for this helpful advice to have a clear expression of our work. Here we should use assembled viral contigs. Therefore, we revised "We aligned the genome sequences with reference virus sequences using BLASTn to assign virus taxon labels" as "We aligned the assembled contigs with reference virus sequences using BLASTn to assign virus taxon labels". (Please refer to

Line 149-151, Page 5 in the revised manuscript.)

Minor comment 11: *Tables 1 and 2, perhaps consider presenting these results as plots? I feel that the tables are rather hard to process.*

Response:

We are really thankful for Reviewer 2's comment. To better represent the results, we have changed the tables to figures and have rearranged the order of other figures in the revision manuscript, as shown in the newly updated figures.

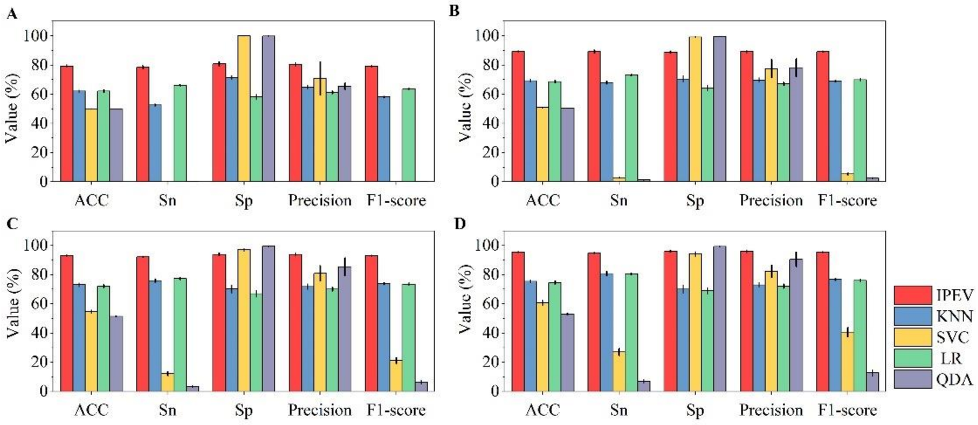

Figure 2. Panels A, B, C, and D display the comparative performance of IPEV and HTP (KNN, SVC, LR, and QDA) with 5-fold cross-validation across Groups A, B, C, and D, respectively. * Sn = TP / (TP + FN), Sp = TN / (TN + FP), ACC = (TP + TN) / (TP + TN + FP + FN), Precision = TP / (TP + FP), F1-score = 2 × Precision × Recall / (Precision + Recall), where TP, TN, FP, and FN respectively represent true positive, true negative, false positive, and false negative. As the method with the best performance in HTP, KNN is selected for comparison. The mean and standard deviation of 5-fold cross-validation are computed to elaborate on performance evaluation. Due to a lack of reconstruction between the train and validation set, the performance of HTP (KNN) is overestimated. (In this paper, prokaryotic viruses are treated as positive samples.)

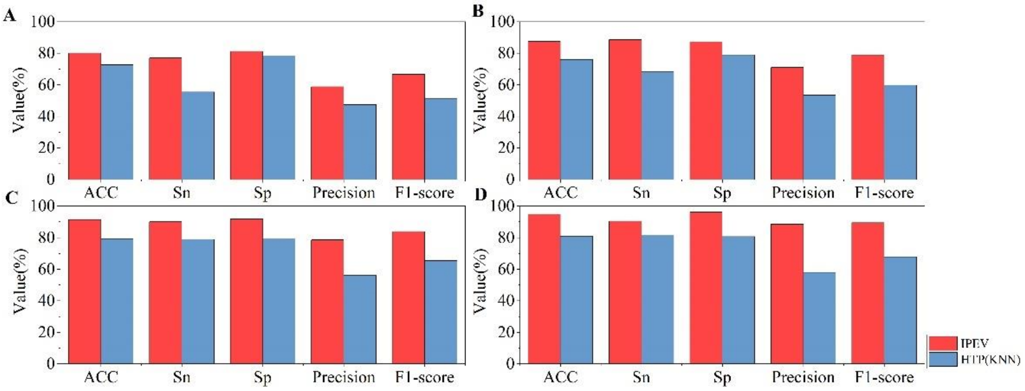

Figure 4. Panels A, B, C, and D display the comparative performance of IPEV and HTP (KNN) across Groups A, B, C, and D, respectively, of Dataset I1. (parameter: query coverage = 30%, identity = 30%).

Minor comment 12: *Line 274: This is a rather old reference, are you sure the error rate for PacBio is still this high? I would suggest looking at more up-to-date references.*

Response:

We appreciate Reviewer 2's constructive comment and recognize the concern regarding the error rate for PacBio sequencing. It is indeed true that the error rate of 10-15% has been widely accepted in the literature. However, with advancements in sequencing technology, lower error rates have been reported. We have accordingly updated our manuscript to reflect these developments by including two recent publications (Dohm et al., Benchmarking of long-read correction methods, 2020; Pourmohammadi et al., A. Error analysis of the PacBio sequencing CCS reads, 2023) that provide a more current analysis of error rates. Consequently, we have revised the text and updated citations as "while third-generation sequencing, such as PacBio, even exhibits a higher error rate of 5% to 15% per base" in Subsection "Performance on test sets with sequencing errors" (please refer to Line 278-279, Page 10 in the revised manuscript).

In addition, we added the references into Section "References":

"Dohm JC, Peters P, Stralis-Pavese N et al. Benchmarking of long-read correction methods, NAR: Genomics Bioinf. 2020;2(2):lqaa037."

"Pourmohammadi R, Abouei J, Anpalagan A. Error analysis of the PacBio sequencing CCS reads, The International Journal of Biostatistics. 2023."

Minor comment 13: *Line 279: replace "base insert or delete" with "insertions or deletions".*

Response:

We are thankful for Reviewer 2's comment. We have replaced "base insert or delete" with "insertions or deletions". (Please refer to Line 284, Page 10 in the revised manuscript.) Meanwhile, we updated all related texts.

Minor comment 14: *Table 3: Indicate the length range of the analysed sequences in the header.*

Response:

We are grateful for Reviewer 2's advice. We have indicated the artificial datasets (1,200-1,800 bp) in the header of Table 1 as "Comparison of IPEV and HTP's AUC value on artificial datasets (1,200-1,800 bp) with varying error rates." (Please refer to Line 292, Page 10 in the revised manuscript.)

Minor comment 15: *The section regarding the performance on functional proteins seems to include information that should be split between methods and results. Please modify accordingly.*

Response:

We are grateful for Reviewer 2's suggestion. We have now appropriately split the information between the methods and results sections. We extremely appreciate Reviewer 2's attention to detail, which has helped improve the clarity and structure of our manuscript. We therefore moved the sentences "Furthermore, we assessed the capability of the IPEV tool by analyzing protein sequences with functional annotations. We constructed a dataset of 7,384 RBPs and corresponding negative samples, which were manually verified. Our selection criteria revolved around methods that are oriented towards function, GO annotation, or product description and that feature RBP-related keywords. These protein sequences originated from a wide range of prokaryotic viruses, spanning seven orders and 28 families, including *Tubulavirales* and *Timlovirales*. To evaluate our model's efficacy in predicting eukaryotic viruses, we also assembled a collection of seven experimentally confirmed capping enzymes" into Subsection "Dataset construction" of Section "MATERIALS AND METHODS" from Section "REULTS". (Please refer to Line 128-135, Page 5 in the revised manuscript.)

Minor comment 16: *Please italicise names of viral taxa wherever they are mentioned in the*

manuscript (e.g., Tubulavirales and Timlovirales in Line 300).

Response:

We are thankful to Reviewer 2 for reminding us to italicise names of viral taxa. In this revision, all the viral taxa mentioned in the manuscript have been italicised. For example:

-- "These protein sequences originated from a wide range of prokaryotic viruses, spanning seven orders and 28 families, including *Tubulavirales* and *Timlovirales*. To evaluate our model's efficacy in predicting eukaryotic viruses, we also assembled a collection of seven experimentally confirmed capping enzymes." (please refer to Line 134-137, Page 5 in the revised manuscript.)

-- "… virulent *CrAss-like* and *Microviridae phages* …" (Please refer to Line 364 Page 13 in the revised manuscript.)

Minor comment 17: *Line 320: This sounds as if the authors had conducted the experiments to collect the gut virome data. Rewrite to make it clear that these data were retrieved from a previous study.*

Response:

We are sorry for the misunderstanding. In the current study, we performed the analysis just based on a collected dataset comprising 130 samples collected from 10 subjects, which were originally published in Shkoporov et al.'s study. Therefore, we revised "Our study involved a cohort of 130 samples collected from 10 subjects. The raw data underwent processing and annotation following the methodology outlined in the Materials and Methods section." as "In our study, we analyzed a dataset comprising 130 samples collected from 10 subjects, which were originally obtained in Shkoporov et al.'s study. We processed and annotated the raw data following the methodology outlined in the Materials and Methods section." (Please refer to Line 330-332, Page 12 in the revised manuscript.)

Minor comment 18: *Line 331: Based on which observation did you reach this conclusion?*

Response:

We thank Reviewer 2 for this helpful advice to have a clear expression of our work. In our study, we reached our conclusion based on the fact that the performance with median 0.51 AUC scores of QDA is equivalent to random guessing. To this end, we revised "We observed that the

performance of QDA is equivalent to random guessing" as "We observed that the performance of QDA is equivalent to random guessing, with median 0.51 AUC scores" in Subsection "Applying IPEV to analyze the longitudinal gut virome in a cohort study" of Section "RESULTS". (Please refer to Line 343-344, Page 13 in the revised manuscript.)

Minor comment 19: *Line 368: Wasn't HTP developed for addressing a similar question? Please clarify.*

Response:

We appreciate Reviewer 2's good question. In our manuscript, we intended to emphasize that our approach is specifically designed for virome data, which typically comprises shorter sequence fragments. HTP, while developed for similar analyses, did not originally consider the unique characteristics of virome data during designing. Therefore, we can confirm that our statement "… IPEV is the first de novo identification algorithm tool developed to address this type of problem for virome data" in Line 381-382 in Section "DISCUSSION".

Minor comment 20: *Line 409-410: The way the sentence is written seems to indicate that plant viruses can also infect human cells and microorganisms. Please rewrite to make it clearer.*

Response:

We are thankful to Reviewer 2 for pointing out this unclear expression. We revised "We utilized the IPEV approach to conduct a comprehensive analysis of longitudinal data from the gut virome, encompassing eukaryotic and prokaryotic viruses, with the former including plant viruses primarily acquired from the environment and diet. These viruses have the ability to infect various entities, including human cells and microorganisms (such as bacteria and archaea)" as "We utilized the IPEV approach to conduct a comprehensive analysis of longitudinal data from the gut virome, which includes eukaryotic viruses primarily acquired from the environment and diet as well as prokaryotic viruses that target microorganisms." (Please refer to Line 406-408, Page 14 in the revised manuscript.)

Minor comment 21: *Regarding the tool's text output, I would suggest modifying it to make it easier to parse (for example, leaving it as a tabular .csv file), and currently the header does not seem to*

accurately describe the contents of the file.

Response:

We are grateful for Reviewer 2's suggestion. Thank you for your insightful suggestions. We have made significant modifications to our main program for its text output. Now, after processing the input sequences, the program generates a TSV file with headers named: "Sequence_ID", "Prokaryotic_Virus_Score", "Eukaryotic_Virus_Score", and "Virus_Taxon". The "Sequence_ID" column will document the header information from the input sequences. We are genuinely appreciative of your advice and believe that these changes enhance the overall utility and user experience of our tool. In the revised work, the below texts are shown in website of the tool:

Sequence Scoring File: The final scores for the sequences will be stored in a TSV (Tab-Separated Values) file. This file is placed in a folder named with the current date and time. The TSV file includes scores for each sequence from your FASTA file, structured as follows:

Sequence_ID	Prokaryotic_Virus_Score	Eukaryotic_Virus_Score	Virus_Taxon
Sample_ID	Score1	Score2	Category

In hoping that the above revision has clarified all the points by two reviewers and given a point-by-point response to all the concerns, we hereby resubmit our manuscript to the journal. We thank you for your kind consideration.

Sincerely yours,

Huaiqiu Zhu, Ph. D., Professor

Peking University

References

Ahlgren, N.A., Ren, J., Lu, Y.Y., Fuhrman, J.A., and Sun, F. (2017). Alignment-free d_2 oligonucleotide frequency dissimilarity measure improves prediction of hosts from metagenomically-derived viral sequences. Nucleic Acids Res 45, 39-53.

Jang, H.B., Bolduc, B., Zablocki, O., Kuhn, J.H., Roux, S., Adriaenssens, E.M., Brister, J.R., Kropinski, A.M., Krupovic, M., Lavigne, R., et al. (2019). Taxonomic assignment of uncultivated prokaryotic virus genomes is enabled by gene-sharing networks. Nat Biotechnol 37, 632-639.

Camargo, A.P., Nayfach, S., Chen, I.A., Palaniappan, K., Ratner, A., Chu, K., Ritter, S.J., Reddy, T.B.K., Mukherjee, S., Schulz, F., et al. (2023). IMG/VR v4: an expanded database of uncultivated virus genomes within a framework of extensive functional, taxonomic, and ecological metadata. Nucleic Acids Res 51, D733-D743.

Fang, Z., Tan, J., Wu, S., Li, M., Xu, C., Xie, Z., and Zhu, H. (2019). PPR-Meta: a tool for identifying phages and plasmids from metagenomic fragments using deep learning. Gigascience 8.

Galiez, C., Siebert, M., Enault, F., Vincent, J., and Söding, J. (2017). WIsH: who is the host? Predicting prokaryotic hosts from metagenomic phage contigs. Bioinformatics 33, 3113-3114.

Goodacre, N., Aljanahi, A., Nandakumar, S., Mikailov, M., and Khan, A.S. (2018). A Reference Viral Database (RVDB) To Enhance Bioinformatics Analysis of High-Throughput Sequencing for Novel Virus Detection. mSphere 3.

Gregory, A.C., Zayed, A.A., Conceição-Neto, N., Temperton, B., Bolduc, B., Alberti, A., Ardyna, M., Arkhipova, K., Carmichael, M., and Cruaud, C. (2019). Marine DNA viral macro-and microdiversity from pole to pole. Cell 177, 1109-1123. e1114.

Guo, J., Bolduc, B., Zayed, A.A., Varsani, A., Dominguez-Huerta, G., Delmont, T.O., Pratama, A.A., Gazitúa, M.C., Vik, D., Sullivan, M.B., et al. (2021). VirSorter2: a multi-classifier, expert-guided approach to detect diverse DNA and RNA viruses. Microbiome 9.

Hyatt, D., Chen, G.L., Locascio, P.F., Land, M.L., Larimer, F.W., and Hauser, L.J. (2010). Prodigal: prokaryotic gene recognition and translation initiation site identification. BMC Bioinformatics 11, 119.

Lu, C., Zhang, Z., Cai, Z., Zhu, Z., Qiu, Y., Wu, A., Jiang, T., Zheng, H., and Peng, Y. (2021). Prokaryotic virus host predictor: a Gaussian model for host prediction of prokaryotic viruses in metagenomics. BMC Biol 19, 5.

Mihara, T., Nishimura, Y., Shimizu, Y., Nishiyama, H., Yoshikawa, G., Uehara, H., Hingamp, P., Goto, S., and Ogata, H. (2016). Linking Virus Genomes with Host Taxonomy. Viruses 8, 66.

Rangel-Pineros, G., Almeida, A., Beracochea, M., Sakharova, E., Marz, M., Reyes Muñoz, A., Hölzer, M., and Finn, R.D. (2023). VIRify: an integrated detection, annotation and taxonomic classification pipeline using virus-specific protein profile hidden Markov models. PLOS Computational Biology 19, e1011422.

Roux, S., Camargo, A.P., Coutinho, F.H., Dabdoub, S.M., Dutilh, B.E., Nayfach, S., and Tritt, A. (2023). iPHoP: An integrated machine learning framework to maximize host prediction for metagenome-derived viruses of archaea and bacteria. PLoS Biol 21, e3002083.

Roux, S., Enault, F., Hurwitz, B.L., and Sullivan, M.B. (2015). VirSorter: mining viral signal from microbial genomic data. PeerJ 3, e985.

Schoch, C.L., Ciufo, S., Domrachev, M., Hotton, C.L., Kannan, S., Khovanskaya, R., Leipe, D., McVeigh, R., O'Neill, K., Robbertse, B., et al. (2020). NCBI Taxonomy: a comprehensive update on curation, resources and tools. Database (Oxford) 2020.

Shang, J., Jiang, J., and Sun, Y. (2021). Bacteriophage classification for assembled contigs using graph convolutional network. Bioinformatics 37, i25-i33.

Tan, J., Fang, Z., Wu, S., Guo, Q., Jiang, X., and Zhu, H. (2021). HoPhage: an ab initio tool for identifying hosts of phage fragments from metaviromes. Bioinformatics 38, 543-545.

Wu, S., Fang, Z., Tan, J., Li, M., Wang, C., Guo, Q., Xu, C., Jiang, X., and Zhu, H. (2021). DeePhage: distinguishing virulent and temperate phage-derived sequences in metavirome data with a deep learning approach. GigaScience 10, giab056.

Zhang, F., Zuo, T., Yeoh, Y.K., Cheng, F.W., Liu, Q., Tang, W., Cheung, K.C., Yang, K., Cheung, C.P., and Mo, C.C. (2021). Longitudinal dynamics of gut bacteriome, mycobiome and virome after fecal microbiota transplantation in graft-versus-host disease. Nature communications 12, 65.

Zolfo, M., Pinto, F., Asnicar, F., Manghi, P., Tett, A., Bushman, F.D., and Segata, N. (2019). Detecting contamination in viromes using ViromeQC. Nature biotechnology 37, 1408-1412.
