# Supplementary material for: IPEV: identification of prokaryotic and eukaryotic virus-derived sequences in virome using deep learning
Source: Gigascience. 2024 Apr 22;13:giae018. doi: 10.1093/gigascience/giae018 (PMC11034026; doi:10.1093/gigascience/giae018)
Supplement: giae018_GIGA-D-23-00357_Revision_1 [file giae018_giga-d-23-00357_revision_1.pdf]

# GigaScience

# IPEV: identification of prokaryotic and eukaryotic virus-derived sequences in virome using deep learning

--Manuscript Draft--

| | |
|---|---|
| **Manuscript Number:** | GIGA-D-23-00357R1 |
| **Full Title:** | IPEV: identification of prokaryotic and eukaryotic virus-derived sequences in virome using deep learning |
| **Article Type:** | Technical Note |
| **Funding Information:** | National Key Research and Development Program of China (2021YFC2300300) — Dr Huaiqiu Zhu<br>National Natural Science Foundation of China (32070667) — Dr Huaiqiu Zhu<br>National Natural Science Foundation of China (31671366) — Dr Huaiqiu Zhu<br>National Natural Science Foundation of China (32300078) — Dr Xiaoqing Jiang |
| **Abstract:** | Background: The virome obtained through virus-like particle enrichment contains a mixture of prokaryotic and eukaryotic virus-derived fragments. Accurate identification and classification of these elements are crucial to understanding their roles and functions in microbial communities. However, the rapid mutation rates of viral genomes pose challenges in developing high-performance tools for classification, potentially limiting downstream analyses.<br>Findings: We present IPEV, a novel method to distinguish prokaryotic and eukaryotic viruses in viromes, with a 2D convolutional neural network combining trinucleotide pair relative distance and frequency. Cross-validation assessments of IPEV demonstrate its state-of-the-art precision, significantly improving the F1-score by approximately 22% on an independent test set compared to existing methods when query viruses share less than 30% sequence similarity with known viruses. Furthermore, IPEV outperforms other methods in accuracy on marine and gut virome samples based on annotations by sequence alignments. IPEV reduces runtime by at most 1,225 times compared to existing methods under the same computing configuration. We also utilized IPEV to analyze longitudinal samples and found that the gut virome exhibits a higher degree of temporal stability than previously observed in persistent personal viromes, providing novel insights into the resilience of the gut virome in individuals.<br>Conclusions: IPEV is a high-performance, user-friendly tool that assists biologists in identifying and classifying prokaryotic and eukaryotic viruses within viromes. The tool is available at https://github.com/basehc/IPEV. |
| **Corresponding Author:** | Huaiqiu Zhu<br>Peking University<br>Beijing, CHINA |
| **Corresponding Author Secondary Information:** | |
| **Corresponding Author's Institution:** | Peking University |
| **Corresponding Author's Secondary Institution:** | |
| **First Author:** | Hengchuang Yin |
| **First Author Secondary Information:** | |
| **Order of Authors:** | Hengchuang Yin<br>Shufang Wu<br>Jie Tan |

Powered by Editorial Manager® and ProduXion Manager® from Aries Systems Corporation

| | |
|---|---|
| | Qian Guo |
| | Mo Li |
| | Jinyuan Guo |
| | Yaqi Wang |
| | Xiaoqing Jiang |
| | Huaiqiu Zhu |
| **Order of Authors Secondary Information:** | |
| **Response to Reviewers:** | Dear Editor,<br><br>Thank you very much for your previous Email on March 9, 2024, regarding our manuscript, “IPEV: identification of prokaryotic and eukaryotic virus-derived sequences in virome using deep learning” (GIGA-D-23-00357). We are very pleased to learn that our manuscript is acceptable for publication in GigaScience, subject to the completion of revisions suggested by the reviewer. Herein, we would like to express our gratitude to you, and the expert member of the Editorial Board, and Reviewer 1, for your thorough review. We are also pleased to note that our responses have addressed all of their concerns, that the “expert and the previous referee agree that the revisions are satisfactory”. Additionally, we are grateful for their careful reading of the previous version of our manuscript, and the further comments raised by the reviewer helped us make further improvements.<br><br>Following your suggestion, we have registered our new software application in the bio.tools database to receive a BioTools ID (biotools:IPEV), and we have included this ID in the latest version of the manuscript following the RRID.<br><br>In this revision, we made several minor changes (reported below) exactly followed Reviewer 1’s and your points. In addition, we have carefully reviewed the journal’s style and ensured that the revised manuscript conforms to it. At this stage, we have removed the tracking of changes or highlighting in red that was made for the purpose of peer review in the revised manuscript. We then report our revisions and point-by-point responses to Reviewer 1’s comments (in italic text) one by one as follows:<br><br>Reviewer 1 :<br>Comment 1: The authors include some text in the Discussion section (paragraph from line 423 to line 436, and paragraph from line 437 to 448) that, in my opinion, would fit better in the Results section. I suggest the authors include these in the Results section, and then in the Discussion comment how those results compare to other methods and what are their implications.<br>We are deeply grateful for Reviewer 1's suggestions. Following the suggestion of Review 1, we have relocated the below sentences into Subsection “Performance on viral genome fragments using cross-validation” as follows:<br>“In addition, we plotted the training loss, validation loss, training accuracy, and validation accuracy curves with respect to the number of epochs using 5-fold cross-validation. We observed that the model converged at 30 epochs, with training and validation losses and accuracies remaining consistent and overlapping. This indicates that the model achieved high performance while avoiding overfitting or underfitting the data, as shown in Figures S8 and S9”. (Please refer to Line 232-236, Page 8 in the revised manuscript.)<br>“We also test IPEV’s effectiveness in differentiating viral from non-viral genome fragments in datasets where viruses and non-viruses are present in a 50:50 ratio. As shown in Figure S12, across Groups A to D, IPEV achieved Sn scores of 0.73, 0.833, 0.905, and 0.931, respectively (details on sample construction and methods can be found in Supplementary Materials and Methods).” (Please refer to Line 236-240, Page 8 in the revised manuscript.)<br><br>Comment 2: I would suggest modifying the sentence in line 42 like this: “Nonetheless, it is essential to note that enriched sample approaches carry the risk of losing valuable host or environmental information [8], potentially leading to inaccurate virus host identification and constraining subsequent analyses.”<br>We are very thankful for Reviewer 1’s comment. We have revised the sentence |

| | |
|---|---|
| | "Nonetheless, it is essential to note that enriched sample approaches carry the risk of losing valuable host or environmental information [8] and can cause inaccurate virus host identification, thereby constraining subsequent analyses." as "Nonetheless, it is essential to note that enriched sample approaches carry the risk of losing valuable host or environmental information [8], potentially leading to inaccurate virus host identification and constraining subsequent analyses." (Please refer to Line 42-44, Page 2 in the revised manuscript.)<br><br>Comment 3: In the sentence starting in line 392, instead of "During" use "For".<br>We are very thankful for Reviewer 1's comment. We have replaced "During" with "For". Therefore, we have revised "During artificial contigs with less than 30% sequence homology against the training set" as "For artificial contigs with less than 30% sequence homology against the training set." (Please refer to Line 192, Page 14 in the revised manuscript.)<br><br>In hoping that the above revision has clarified all the points by Reviewer 1 and given a point-by-point response to all the concerns, we hereby resubmit our manuscript to the journal. We thank you for your kind consideration. Please do not hesitate to let us know if additional edits are needed. We will be no more than willing to meet your requirements.<br><br>Sincerely yours,<br>Huaiqiu Zhu, Ph. D., Professor<br>Peking University |
| **Additional Information:** | |
| **Question** | **Response** |
| Are you submitting this manuscript to a special series or article collection? | No |
| **Experimental design and statistics**<br><br>Full details of the experimental design and statistical methods used should be given in the Methods section, as detailed in our Minimum Standards Reporting Checklist. Information essential to interpreting the data presented should be made available in the figure legends.<br><br>Have you included all the information requested in your manuscript? | Yes |
| **Resources**<br><br>A description of all resources used, including antibodies, cell lines, animals and software tools, with enough information to allow them to be uniquely identified, should be included in the Methods section. Authors are strongly encouraged to cite Research Resource Identifiers (RRIDs) for antibodies, model organisms and tools, where possible. | Yes |

| | |
|---|---|
| Have you included the information requested as detailed in our Minimum Standards Reporting Checklist? | |
| **Availability of data and materials**<br><br>All datasets and code on which the conclusions of the paper rely must be either included in your submission or deposited in publicly available repositories (where available and ethically appropriate), referencing such data using a unique identifier in the references and in the “Availability of Data and Materials” section of your manuscript.<br><br>Have you have met the above requirement as detailed in our Minimum Standards Reporting Checklist? | Yes |

Manuscript Click here to access/download;Manuscript;IPEV_R2_revised.docx

# IPEV: identification of prokaryotic and eukaryotic virus-derived sequences in virome using deep learning

Hengchuang Yin[1], Shufang Wu[1], Jie Tan[1], Qian Guo[1], Mo Li[1,2], Jinyuan Guo[1,3], Yaqi Wang[1], Xiaoqing Jiang[1,4] and Huaiqiu Zhu[1,2,3]*

[1] *Department of Biomedical Engineering, College of Future Technology, and Center for Quantitative Biology, Peking University, Beijing 100871, Beijing, China;*

[2] *School of Life Sciences, Peking University, Beijing 100871, Beijing, China;*

[3] *Department of Biomedical Engineering, Georgia Institute of Technology and Emory University, GA 30332, Atlanta, USA*

[4] *Beijing Institute of Genomics, Chinese Academy of Sciences, and China National Center for Bioinformation, Beijing 100101, China*

* To whom correspondence should be addressed. Tel: 8610-6276 7261; Email: hqzhu@pku.edu.cn

## Abstract

**Background:** The virome obtained through virus-like particle enrichment contains a mixture of prokaryotic and eukaryotic virus-derived fragments. Accurate identification and classification of these elements are crucial to understanding their roles and functions in microbial communities. However, the rapid mutation rates of viral genomes pose challenges in developing high-performance tools for classification, potentially limiting downstream analyses.

**Findings:** We present IPEV, a novel method to distinguish prokaryotic and eukaryotic viruses in viromes, with a 2D convolutional neural network combining trinucleotide pair relative distance and frequency. Cross-validation assessments of IPEV demonstrate its state-of-the-art precision, significantly improving the F1-score by approximately 22% on an independent test set compared to existing methods when query viruses share less than 30% sequence similarity with known viruses. Furthermore, IPEV outperforms other methods in accuracy on marine and gut virome samples based on annotations by sequence alignments. IPEV reduces runtime by at most 1,225 times compared to existing methods under the same computing configuration. We also utilized IPEV to analyze longitudinal samples and found that the gut virome exhibits a higher degree of temporal stability than previously observed in persistent personal viromes, providing novel insights into the resilience of the gut virome in

individuals. **Conclusions:** IPEV is a high-performance, user-friendly tool that assists biologists in identifying and classifying prokaryotic and eukaryotic viruses within viromes. The tool is available at https://github.com/basehc/IPEV.

## INTRODUCTION

Viruses and virus-like particles (VLPs) are abundant and diverse biological entities on Earth. It is estimated that there are approximately $10^{31}$ viral particles [1], showcasing their pervasive nature. Notably, even in human feces, there can be as many as $10^9$ VLPs per gram, further emphasizing their prevalence and ubiquity [2, 3]. The advent of next-generation sequencing (NGS) technology has revolutionized virome studies, enabling the discovery of novel viruses and significantly advancing our understanding of their potential influence on both environmental and human body microbiomes [4-7]. Nonetheless, it is essential to note that enriched sample approaches carry the risk of losing valuable host or environmental information [8], potentially leading to inaccurate virus host identification and constraining subsequent analyses. A noteworthy example is our previous study, in which we found a strong correlation between alterations in the proportion of temperate phages within the gut virome and the occurrence of ulcerative colitis in patients [9]. Similarly, the significance of the eukaryotic virome should not be overlooked when examining the virome, as it is believed to play a vital role in both host health and disease [10-14]. Recent studies have shed light on intricate trans-kingdom interactions involving eukaryotic viruses, bacteria, and the host within the intestinal ecosystem [15-18]. Obviously, these analyses first require the precise differentiation of eukaryotic viruses from prokaryotic viral sequences, and it's beneficial for gaining a comprehensive understanding of the viral landscape [9, 15].

However, the precise identification of prokaryotic and eukaryotic viruses poses a significant challenge owing to the highly diverse and fast-mutating genetic elements within the virome. Furthermore, the limitations of assembly tools arise from factors such as mutations, recombination events, and often low or uneven sequencing coverage across the viral genome [19]. The abundance of short and frequently inadequate reads further complicates the task of virus identification. Moreover, the absence of a well-conserved genetic marker such as the bacterial 16S rRNA gene presents a barrier in constructing a phylogenetic tree to effectively differentiate between eukaryotic and prokaryotic viruses [20]. Virus identification typically involves aligning sequences against known viruses in genomic repositories, such as the National Center for Biotechnology Information (NCBI) Taxonomy Databases

[21] and European Nucleotide Archive (ENA) [22]. Despite the rapid increase in viral sequences, the number of viral reference sequences in public reference databases is still limited, constraining the effectiveness of sequence-based alignment methods. For instance, it has been estimated that there are millions of viral species, but the International Committee on Taxonomy of Viruses (ICTV) has recognized only 11,273 species to date [23].

Currently, some computational tools for identifying viruses have been introduced in metagenomes, such as HoPhage [24], iPHoP [25, 26], WIsH [27], CHERRY [28], PHP [29], and VHM-Net [30], designed to assign the host for a given phage contig using sequence similarity search or ab initio identification. Some tools, like DeePhage [9], PHACTS [31], and PhagePred [32], have been developed to answer questions about the lifestyles of phages. Other tools, like PPR-Meta [33], DeepVirFinder [34], VIBRANT [35], vConTACT2 [36], VirSorter [37], and the most recent version, VirSorter2 [38], can be used to identify viruses from metagenomics data. While analyzing and classifying phages in virome data is a current focus, it is important to note that eukaryotic viruses also play a critical role in influencing host immunity and disease phenotypes by infecting host cells and interacting with the bacterial microbiome through trans-kingdom interactions. However, existing methods for analyzing virome data are limited in distinguishing eukaryotic viruses from viromes, assigning hosts, and classifying them accurately [39]. Some methods, such as the Host Taxon Predictor (HTP) [40], can be used to bridge the gap, differentiating between phages and eukaryotic viruses based on sequence information and nucleic acid type (such as DNA or RNA). Given that HTP's performance is highly dependent on the nucleic acid type used, and that some experimental protocols for virome research may result in mixed datasets containing both DNA and RNA sequences [41], it might be challenging accurately determine the origin of these sequences. This uncertainty could, in our observations, influence the performance of HTP in classifying viruses.

In this paper, we present the IPEV (Identify Prokaryotic and Eukaryotic Virus-derived sequences), a high-performance, user-friendly tool for differentiating prokaryotic and eukaryotic viruses from virome sequence fragments. To achieve high performance on short viral sequences, we developed a 2D convolutional neural network (CNN) based on a sequence pattern matrix using the Sequence Graph Transform (SGT) model. Cross-validation tests demonstrate that IPEV significantly outperforms related methods in terms of F1-score metrics by at most 21.1% while requiring only 1/50th the time of HTP in the same computing environment. We also designed various homology layouts for independent sets

based on known sequence data to assess IPEV's generalization capabilities. IPEV outperforms HTP (KNN) by approximately 22% in terms of the F1-score on an independent set with highly imbalanced labels when the sequence identity between the training and independent test sets is less than 30%. IPEV's evaluations of marine virome samples are better than HTP, with much more accurate results. We applied IPEV to analyse the longitudinal gut virome data from ten healthy individuals over 12 months and achieved the best performance in at least 90% of the samples compared to other methods. Our analysis revealed that the gut virome exhibits temporal stability beyond that observed in persistent personal viromes, thus enhancing our understanding of gut virome stability in individuals.

## MATERIALS AND METHODS

### Dataset construction

Without accurately host-annotated virome datasets that could serve as benchmarks, we generated simulated datasets based on well-annotated complete virus genomes. Firstly, we downloaded the taxonomy ID list of viruses and corresponding host lineages from Virus-Host DB [42] and genome sequences from the NCBI database [43] on Oct. 31, 2021. As a result, we established our first dataset, referred to as Dataset-1, which contains 11,022 eukaryotic virus genomes and 5,051 prokaryotic virus genomes (of which 113 are attributed to archaeal viruses). To enhance the model's generalizability, we incorporated additional data from the Reference Viral Database (RVDB) [44] and 25,644 eukaryotic sequences along with 5,598 prokaryotic sequences from IMG/VR v4 [45], collectively termed Dataset-2. Details regarding the data inclusion criteria are outlined in the Supplementary Materials and Methods. Dataset-1 is sourced from reference sequences and manually curated with credible host annotations, while Dataset-2 is not. Therefore, based on genomes, we used all viruses in Dataset-2 and 10,000 eukaryotic and 4,000 prokaryotic viruses in Dataset-1 randomly divided for 5-fold cross-validation, while the remaining subset served as an independent test set for assessing generalizability.

Considering the limitations of current mainstream sequencing technologies and the length constraints of assembled contigs, we simulated four contig length groups (A-D) using MetaSim (v0.9.1) [46] with "exact" pre-set and "Uniform" distribution types. The contig length groups were as follows: Group A (100-400 bp), Group B (400-800 bp), Group C (800-1,200 bp), and Group D (1,200-1,800 bp). The specific contig numbers can be found in Tables S1 and S2 in the Supplementary Materials. We evaluated IPEV's generalization ability using an independent test set of 1,022 eukaryotic and 1,051

prokaryotic virus sequences. Using MetaSim, we totally generated 20,000 contigs and ensured low similarity against the training set using BLASTn (v2.7.1), following the above length groups. We generated six low homology independent test sets (Dataset I1-I6) with varying query coverage and identity thresholds relative to the training set. The number of corresponding prokaryotic and eukaryotic virus contigs in each independent test set can be found in Table S5 and Table S6, respectively.

We evaluated the effect of sequencing errors on IPEV's performance, and we generated a total of 10,000 contigs (1,200 to 1,800 bp) with 5%, 10%, and 15% sequencing errors based on the independent test set using MetaSim (related details can be found in the Supplementary Materials and Methods). Furthermore, we assessed the capability of the IPEV tool by analyzing protein sequences with functional annotations. We constructed a dataset of 7,384 RBPs and corresponding negative samples, which were manually verified. Our selection criteria revolved around methods that are oriented towards function, GO annotation, or product description and that feature RBP-related keywords. These protein sequences originated from a wide range of prokaryotic viruses, spanning seven orders and 28 families, including *Tubulavirales* and *Timlovirales*. To evaluate our model's efficacy in predicting eukaryotic viruses, we also assembled a collection of seven experimentally confirmed capping enzymes [47].

We also use real virome to evaluate IPEV and related tools. We first downloaded a dataset comprising 243 marine virome samples from the ENA (accession number: PRJEB22493 [48, 49]). Besides, we analyzed longitudinal data from Shkoporov et al.'s [50] study to evaluate IPEV's accuracy and the stability of gut virome data. We retrieved the raw human gut virome dataset from the NCBI SRA (accession number: PRJNA545408). This dataset included 130 virome samples from ten healthy adults (subjects 916-925) collected over 12 months (T1-T12) through monthly synchronous samplings. We utilized the SPAdes (v3.13.0) [51] software to assemble short reads and conducted BLASTn searches against a bacterial database to eliminate bacterial contamination with an e-value of e-5, an identity of 50%, and query coverage of 90%. Our reference bacterial dataset comprised 20,003 complete prokaryotic genomes sourced from the NCBI RefSeq database, comprising 19,629 bacterial genomes and 374 archaeal genomes. Following Shkoporov's personal persist virome (PPV) definition, we used cd-hit-est (v.4.8.1) with parameters (c 0.8, aS 0.8, d 0, n 5) to cluster decontaminated contigs and defined clusters containing contigs from at least six months as PPV clusters. For subject 917, sampled for 11 months, we modified the PPV definition to include contigs appearing in at least five months. We aligned the assembled contigs with reference virus sequences using BLASTn to assign virus taxon

labels. The potential prokaryotic or eukaryotic viruses were inferred from viral contigs with an e-value less than the cutoff of 1e-4.

## Mathematical model of DNA sequences

In this study, we developed a sequence pattern matrix using the Sequence Graph Transform (SGT) model to extract meaningful information based on the relative positions of trinucleotides [52]. The pattern matrix is a numerical representation of the frequency and order of trinucleotide pairs. We generated a trinucleotide set by combining three nucleotides to represent a DNA sequence ($S$) and calculated the weights of trinucleotides u and v using the following formula:

$$\psi_{uv}(s) = \frac{\sum_{\forall(l,m)} e^{-k|m-l|}}{|\Lambda_{uv}(s)|}. \quad (1)$$

Herein, $e^{-\kappa|m-l|}$ represent the weight of a trinucleotide pair of $u$ and $v$ at the position of $m$ and $l$. The relative distances of a trinucleotide pair of $u$ and $v$ are measured by $|m-l|$. $\left|\Lambda_{uv}\right|$ is the size of the set $\Lambda_{uv}$. It represents the size of total $(u, v)$ pairs in the trinucleotide set of a DNA sequence. A schematic representation of the sequence pattern matrix can be found in Figure 1B. Finally, the DNA sequence is converted to a $64 \times 64$ matrix of relationship weights for the trinucleotide pair set.

## Structure of the deep learning neural network

We constructed a 2D convolutional neural network (CNN) to predict taxon information using sequence pattern matrices. The CNN has the below layers: two convolution layers (with a $7 \times 7$ kernel size and the "same" padding), two max pooling layers (with a $2 \times 2$ pooling window), two dropout layers, a flatten layer and two fully connected layers followed by a softmax activation function. Here, the Conv2D layer takes the sequence pattern matrix $\mathrm{I}$ of dimensions ($L \times L$) as the sequence pattern matrix and generates total $\mathrm{F}$ feature maps as output by corresponding $\mathrm{F}$ (kernels) of dimensions $k_1 \times k_2$ with the same padding. Those kernels were used to extract information on the viral sequence. Using ReLU (Rectified Linear Unit) as the activation function, the Conv2D layer outputs an $\mathrm{F} \times k_1 \times k_2$ matrix $Y^C$ and computes the $\mathrm{f}^{\mathrm{th}}$ feature map at the $(i^{\mathrm{th}}, j^{\mathrm{th}})$ location, and the value is given as:

$$(Y_{ij}^C)_f = \mathrm{ReLU}\left(\sum_{m=0}^{k_1-1}\sum_{n=0}^{k_2-1} K_f(m,n) I(i-m, j-n) + b_f^C\right), \quad (2)$$

$$\text{for } i, j = 0,1,2, \ldots, \mathrm{L}-1, \mathrm{f} = 0,1,2, \ldots, \mathrm{F}-1.$$

Where $K^f$ and $b_{\mathrm{f}}^{\mathrm{C}}$ are a $k_1 \times k_2$ weight matrix and a bias of the $\mathrm{f}^{\mathrm{th}}$ kernel. Mainly, the ReLU function mentioned above is defined as follows:

$$\mathrm{ReLU(x)} = \begin{cases} \mathrm{x} & \text{if } \mathrm{x} \geq 0 \\ 0 & \text{if } \mathrm{x} < 0 \end{cases}. \quad (3)$$

The next layer in the model is a Maxpooling layer, taking the maximum value over an input channel with a pooling size $\mathrm{S1} \times \mathrm{S1}$ and a stride size $S2 \times \mathrm{S2}$. The padding option is set to "same". The window is shifted along with each channel independently and can generate F new channels with the size of $\mathrm{L}' \times \mathrm{L}'(\mathrm{L}' = \lceil \mathrm{L}/S2 \rceil)$. The Maxpooling layer outputs an $\mathrm{L}' \times \mathrm{L}' \times \mathrm{F}$ feature matrix $Y^M$ and one of the pooling operations for a specific channel at the $(i^{\mathrm{th}}, j^{\mathrm{th}})$ location was calculated as:

$$Y_{(i,j),\mathrm{f}}^{\mathrm{M}} = \max( Y_{(i\times\mathrm{S2},j\times\mathrm{S2}),\mathrm{f}}^{\mathrm{C}}, Y_{(i\times\mathrm{S2+1},j\times\mathrm{S2+1}),\mathrm{f}}^{\mathrm{C}}, Y_{(i\times\mathrm{S2+2},j\times\mathrm{S2+2}),\mathrm{f}}^{\mathrm{C}}, \dots, Y_{(i\times\mathrm{S2+S1\text{-}1},j\times\mathrm{S2+S1\text{-}1)\text{-}1,f}}^{\mathrm{C}}), \quad (4)$$

$$\text{for } i,j = 0,1,2,\dots,\mathrm{L}' - 1,\ \mathrm{f} = 0,1,2,\dots,\mathrm{F} - 1.$$

The features, the neural network learns in the Maxpooling layer are transferred to the Dropout layer. The output $\tilde{y}^{\mathrm{Dp}}$ is formulated as:

$$\tilde{y}^{\mathrm{Dp}} = \tilde{k} * Y^{\mathrm{M}} \quad \text{Where } \tilde{k} \sim B(1, \mathrm{P}). \quad (5)$$

Here $*$ denotes an element-wise product. For any layer $Y$, The drop mask $\tilde{k}$ denotes an independent Bernoulli distribution with random variables, each having a probability $p$ of 1. It could effectively reduce overfitting. We employed Flatten layer to convert all the elements $\boldsymbol{Y}^{\mathrm{Dp}}$ in the tensor into a $\boldsymbol{Y}^{\mathrm{F}}$one-dimensional array one by one. The Dense1 layer uses the ReLU function to output R units. It has an R×F weight matrix $W^{\mathrm{D1}}$ and an R-dimensional bias vector $b^{\mathrm{D1}}$. Each output unit is given as follows:

$$y_{\mathrm{r}}^{D1} = \mathrm{ReLu}\left(\textstyle\sum_{\mathrm{f}=0}^{\mathrm{F}-1} W_{\mathrm{r,f}}^{D1}\, y_{\mathrm{f}}^{\mathrm{F}} + b_{\mathrm{r}}^{D1}\right), \text{for } \mathrm{r} = 0,1,2,\dots,\mathrm{R}-1. \quad (6)$$

The Dense1 layer can generate an R-dimensional vector $\boldsymbol{y}^{\mathrm{D1}}$ while a Conv1D layer extracts features into different feature maps, and we use a SoftMax function as an activation function. The final layer is the Dense2 layer, which outputs only a $1 \times 2$ dimension array to represent the likelihood of phages and eukaryotic viruses. The output score is calculated as follows:

$$\hat{y}_i^{D2} = \mathrm{softmax}(y_i) = \frac{e^{y_i}}{\sum_{j=1}^{n} e^{y_j}}. \quad (7)$$

Moreover, the loss function is defined below:

$$Loss = -\textstyle\sum_{i=1}^{C} y_i^{D2} \cdot \log \hat{y}_i^{D2}. \quad (8)$$

We employed the Adam optimizer (learning rate = 0.0005) and batch size 16 to train the neural network and update network weights (F = 128, S1 = S2 = 2, P = 0.32, and R = 64). The architecture of

the IPEV neural network is depicted in Figure 1C. When using IPEV, the final viral taxon scores are obtained by weighted averaging of the subsequence predictions. The detailed calculation methodology is outlined in the Supplementary Materials and Methods section.

## RESULTS

### Performance on viral genome fragments using cross-validation

To evaluate the performance of IPEV, we implemented a 5-fold cross-validation procedure on Groups A-D. The HTP tool, which we compared, comprises four distinct classifiers: KNN, SVC, LR, and QDA. The results showed that IPEV performed better than KNN, SVC, LR, and QDA by an average AUC (Area Under the Curve) value increase of 0.16, 0.18, 0.20, and 0.22, respectively, in Group D (1,200-1,800 bp). This is further depicted in Figure 3 and Table S4. Additionally, we observed that the performance of the model's predictions is directly proportional to the nucleotide sequence length. The AUC value of IPEV increased from 0.88 to 0.99, from Group A (100-400 bp) to Group D (1,200-1,800 bp). In contrast, the AUC value of HTP (KNN) increased from 0.66 to 0.83, as shown in Figure S1.

In binary classification, sensitivity (Sn) and specificity (Sp) evaluate the ability to predict positive and negative samples. As shown in Figure 2 and Table S3, In Group A (100–400 bp), the performance of HTP (KNN) in accurately identifying prokaryotic viruses (considered as positive samples) was found to be modest, achieving Sn of 52.6% and Sp of 71.6%. In contrast, our IPEV tool demonstrated rather better performance under the same conditions, with Sn of 78.4% and Sp of 80.9%. This suggests that IPEV outperforms HTP (KNN) in accurately classifying short viral fragments. This implies that for some species with low abundance or insufficient sequencing depth, it may not be possible to assemble longer phage sequences, and HTP may fail to identify them. IPEV, on the other hand, does not exhibit a specific preference. In Group D (1,200–1,800 bp), the performance of HTP (KNN) in accurately identifying eukaryotic viruses was found to be modest, with Sn of 80.8% and Sp of 70.1%. Conversely, under the same conditions, the IPEV tool exhibited superior performance, with Sn of 95.0% and Sp of 96.1%. In addition, we plotted the training loss, validation loss, training accuracy, and validation accuracy curves with respect to the number of epochs using 5-fold cross-validation. We observed that the model converged at 30 epochs, with training and validation losses and accuracies remaining consistent and overlapping. This indicates that the model achieved high performance while avoiding overfitting or underfitting the data, as shown in Figures S8 and S9. We also test IPEV's effectiveness

in differentiating viral from non-viral genome fragments in datasets where viruses and non-viruses are present in a 50:50 ratio. As shown in Figure S12, across Groups A to D, IPEV achieved Sn scores of 0.73, 0.833, 0.905, and 0.931, respectively (details on sample construction and methods can be found in Supplementary Materials and Methods).

**Performance on novel viruses with low homology to known databases.**

Tools designed for predicting viral taxa aim to accurately identify newly discovered viruses, especially those exhibiting low homology to existing viral databases [40, 53, 54]. However, evaluating the performance of such tools is challenging owing to the lack of accurate labels for new viral sequences. This study defines a novel virus as one with very low homology to known viruses. To evaluate IPEV's effectiveness, we constructed several independent test sets in which sequence identity increased compared to the training set of IPEV, these evaluations are designed to measure IPEV's performance on "unseen" viruses. These six low homology test sets (Dataset I1-I6) were generated with varying degrees of query coverage and identity relative to the training set. Related details are shown in the Supplementary Materials and Methods. For Dataset I1, the majority of high homology sequences were eliminated, applying a threshold of 30% identity and 30% coverage. This highly labeled and unbalanced Dataset I1 comprises Groups A, B, C, and D, containing 3,180, 3,152, 3,050, and 3,171 prokaryotic virus contigs, respectively, and 1,106, 1,117, 1,011, and 1,036 eukaryotic virus contigs, respectively.

Despite removing sequences with high homology to the training set, IPEV still outperformed HTP, as shown in Figure 4 and Table S7. Specifically, in Group A (100-400 bp) of Dataset I1, IPEV reported Sn of 76.94% and Sp of 81.19%. On the other hand, HTP (KNN) reported Sn of 55.52% and Sp of 78.55%. In Group D (1,200-1,800 bp), IPEV outperforms HTP(KNN) in Sn and Sp by 10% and 16%, respectively. These results underscore the superior performance of IPEV in distinguishing prokaryotic viruses from eukaryotic viruses. Meanwhile, we observed that as the sequence length increased from Group A (100-400 bp) to Group D (1,200-1,800 bp), the F1-score of IPEV improved from 66.61% to 89.44%. In contrast, the F1-score for HTP (KNN) increased from 51.12% to 67.81%. This performance is consistent with the results of a 5-fold cross-validation. As shown in Figure 4 and Figure 5, in terms of the AUC metric, IPEV outperformed HTP (KNN) by 0.13, 0.11, 0.11, and 0.09 in Groups A, B, C, and D, respectively. These results demonstrate the advantage of IPEV in handling short fragments and datasets with low homology.

Furthermore, to evaluate the performance of IPEV, we utilized Dataset I2-I6, each exhibiting different levels of homology to the training set. As the number of high homology sequences between the independent test set and the training set increases, we observe a gradual improvement in the performance of IPEV, as shown in Table S7-S12 and Figure S3-S7. The results indicate that the similarity between the test and training sets is crucial to determining the classification performance. Table S12 demonstrates that in Group D (1,200-1,800 bp) of Dataset I6 (Query parameter: coverage = 100% identity = 60%), the F1-score that can be achieved with IPEV is 96%. In contrast, the highest F1-score reported with HTP (KNN) is 77%.

On the other hand, it is also important to consider the impact of label imbalance on model performance. In Dataset I1-I6, which vary in their similarity to the training set of IPEV and in their respective length ranges (from Group A to Group D), IPEV's Sn and Sp did not show significant variation as the label imbalance increased, while HTP (KNN) exhibited obvious changes. This indicates that IPEV maintains robust predictive performance, even when tested on sets with low sequence homology to the training set, and does not exhibit a bias in binary classification.

**Performance on test sets with sequencing errors**

In this subsection, we evaluate the performance of IPEV and HTP on a dataset with varying levels of sequencing errors, including base insertion, deletion, and substitution. Errors in NGS are determined by the sequencing method and the experimental environment. Specifically, NGS has an error rate of 0.06% to 0.24% per base, while third-generation sequencing, such as PacBio, even exhibits a higher error rate of 5% to 15% per base [55, 56]. To evaluate the robustness of IPEV, we employed MetaSim to generate 2,000 short reads for both eukaryotic and prokaryotic viruses with different sequencing errors ranging from 1,200 to 1,800 bp. As shown in Table 1, the AUC values for both IPEV and HTP decrease as an increasing proportion of sequencing errors. When the error rate (base substitutions) reaches 15%, IPEV outperforms HTP (KNN) by approximately 16% in terms of the AUC metric. When the error rate (base insertions or deletions) reaches 15%, IPEV outperforms HTP (KNN) by approximately 17% in terms of the AUC metric. The observed phenomenon may be attributed to the sequence pattern matrix's capacity to tolerate errors. Notably, our results indicate that the performance of IPEV is minimally affected by the percentage of sequencing errors introduced. Specifically, when the substitution-induced error rate rises from 0% to 15%, the AUC value declines slightly from 0.99 to 0.91.

These findings underscore the robustness and reliability of IPEV, showing its ability to maintain its performance despite the presence of sequencing errors.

**Table 1.** Comparison of IPEV and HTP's AUC value on artificial datasets (1,200-1,800 bp) with varying error rates.

| Error Rate (%) | Base Substitutions | | Base Insertions or Deletions | |
|---|---|---|---|---|
| | IPEV | HTP | IPEV | HTP |
| 0 | 0.99 | 0.82 | 0.99 | 0.82 |
| 5 | 0.98 | 0.80 | 0.98 | 0.80 |
| 10 | 0.95 | 0.77 | 0.97 | 0.80 |
| 15 | 0.91 | 0.75 | 0.95 | 0.78 |

**Performance on functional protein sequences**

In addition to evaluating short sequence fragments, we comprehensively evaluated the capability of the IPEV tool by incorporating protein sequences with functional annotations. One key aspect of understanding virus classification is identifying critical markers that play a role in the classification process, even though deep learning is often considered a black box.

Considering the crucial role of receptor-binding proteins (RBPs) in the adsorption and host invasion of bacteriophages, we formulated a hypothesis suggesting their substantial contribution to the accuracy of phage prediction. To test this hypothesis, we carefully assembled a dataset of 7,384 RBPs, complemented with corresponding negative samples, verified manually. We aimed to evaluate the impact of RBPs on phage prediction accuracy. The IPEV prediction results showed that RBPs significantly contributed to phage prediction accuracy. The predicted likelihood score by IPEV had a mean of 0.90 and a median of 0.98 for the RBP set, while for the non-RBP set, the mean was 0.77 and the median was 0.83, as shown in Figure 6 (Wilcoxon rank-sum test, p-value $< 2.2e-16$). The findings from our study also indicate that our model possesses the capability to learn host-related information to a certain degree. This emphasizes the significance of integrating host-related data into phage prediction models. To assess our model's performance in predicting eukaryotic viruses, we conducted a focused analysis of seven experimentally confirmed capping enzymes. The results revealed that our model accurately predicted the likelihood of these protein sequences, achieving a score close to 1. This

result provides compelling evidence of the model's high accuracy and effectiveness in predicting eukaryotic viruses. For additional details regarding these results, please refer to Table S14.

## Performance on the marine virome

We collected data from 243 marine virome samples with assembled contigs and annotated them for sequence type using Blastn. We evaluated our tool, IPEV, along with other related tools. We reported the overall average and median AUC for the samples, and we noted that our tool, IPEV, outperforms HTP (KNN, and others) across all samples. As shown in Figure 7, in our comprehensive evaluation, IPEV demonstrated its advanced capabilities by consistently outperforming other tools with its higher average AUC values: it outperforms SVC by 0.18, exceeds QDA by 0.20, surpasses LR by 0.16, and betters KNN by 0.19. These results indicate that IPEV is a highly competitive tool.

## Applying IPEV to analyze the longitudinal gut virome in a cohort study

Within this subsection, we provide a comprehensive overview of our analysis encompassing two primary aspects. Firstly, we assess the accuracy of IPEV, along with its related tool, in analyzing the gut virome. Secondly, we explore the temporal stability of healthy gut virome utilizing longitudinal data spanning a one-year period, employing the IPEV tool.

In our study, we analyzed a dataset comprising 130 samples collected from 10 subjects, which were originally obtained in Shkoporov et al.'s [50] study. We processed and annotated the raw data following the methodology outlined in the Materials and Methods section. We utilized the IPEV tool and its associated software on each distinct sample to generate predictions and compute AUC scores, leveraging BLAST annotation in the process. Our analysis, illustrated in Figure 8A, demonstrated that IPEV exhibited higher accuracy than HTP in over 90% of the real virus samples. Furthermore, IPEV's mean AUC value of 0.64 was significantly superior to those of KNN (0.55), SVC (0.51), LR (0.54), and QDA (0.51), according to the Wilcoxon rank-sum test results using Benjamini-Hochberg adjustment (IPEV with KNN: $P_{adj} < 2.89e-19$; IPEV with SVC: $P_{adj} <1.02e-33$; IPEV with LR: $P_{adj} <6.97e-22$; IPEV with QDA: $P_{adj} < 1.52e-36$).

Additionally, the median AUC for IPEV was 0.63, significantly higher than that of 0.54, 0.52, 0.53, and 0.51 for KNN, SVC, LR, and QDA, respectively. The median difference between the AUC of IPEV and KNN, SVC, LR, and QDA for each sample is shown in Figure 8B, with median differences of 0.09, 0.12, 0.11, and 0.12, respectively. We observed that the performance of QDA is equivalent to random

guessing, with median 0.51 AUC scores. While the performance of IPEV on the simulated dataset was not as remarkable as that on the real virome data, this disparity can be attributed to a notable factor. The assembled sequences in our dataset of 130 samples predominantly comprised short sequences, with those below 500 bp representing a significant portion of 80% of the total. Coping with this challenge, our model persevered and successfully identified viruses within the real virome, demonstrating its effectiveness and robustness.

The human gut virome is characterized by its immense diversity and abundance of virus particles. The gut feces contain up to approximately $10^9$ VLPs per gram, yet only a fraction of the virus genomes, ranging from 14.2% to 56.6%, can be annotated [57]. Previous research [50] has identified a highly individualized and persistent fraction in the gut virome, the personal persistent virome (PPV). Additionally, this research has observed temporal stability in the virus components at the individual level. To further explore the virome, we employed IPEV to ab initio annotate contigs in the virome while excluding bacterial contamination. The results showed that the average coefficient of variation of the phageome was significantly lower (0.04 ± 0.01) compared to the PPV (0.58 ± 0.05), indicating a high degree of temporal stability of the phageome, as illustrated in Figure 8C and Table S15. Our findings align with the studies of Shkoporov et al. and offer further substantiation, this time at a higher taxonomic level, for the hypothesis that the “kill-the-winner” mechanism operates effectively in phage strains and substrains. This mechanism prevents the dominance of any single species, facilitating the coexistence of multiple species instead. Consequently, it enhances the diversity of the gut ecosystem and reinforces its overall resilience [58]. In contrast, the PPV is dominated by lytic life cycles and composed of virulent *CrAss-like* and *Microviridae phages* [50] that infect major representatives of the bacterial microbiota, resulting in higher variations than those seen in gut ecology.

We also observed limited transient disturbance in the phageome component in subjects 916 (T1 and T7) and 922 (T3, T5, and T8) with antibiotic usage. An intriguing observation emerged from our study: despite variations in individual characteristics such as gender, BMI, and lifestyle factors like smoking and alcohol consumption, the relative abundances of the phageome remained remarkably similar among individuals. In contrast, the relative abundances of the PPV displayed substantial individual variations. To delve deeper into these findings, we employed IPEV to assess the contribution of phage and eukaryotic viromes to the variability of PPV. Notably, our analysis revealed that phages constituted the primary component and exerted significant dominance over fluctuations in PPV as

shown in Figure 8C. This highlights the crucial role of phages in driving the variability of PPV. By elucidating these relationships, our results contribute to a better understanding of the overall stability and dynamics of the gut virome.

## DISCUSSION

This study introduces IPEV, a novel method that utilizes a sequence pattern matrix and a 2D convolutional neural network to distinguish prokaryotic and eukaryotic virus-derived sequence fragments. To the author's best knowledge, IPEV is the first de novo identification algorithm tool developed to address this type of problem for virome data.

IPEV offers several advantages over traditional genomics techniques, such as k-mer methods and one-hot encoding. By integrating the position and frequency information of 3-mers into a sequence pattern matrix, IPEV enhances the efficiency of the neural network model and preserves valuable information about the order and position of trinucleotides.

To ensure the generalization evaluation of the presented tool, we opted not to use the traditional approach of dividing the dataset into training and test sets based on the date the sequence was discovered [37, 59]. This method can include sequences with high homology to the training set in the test set, inflating the algorithm's accuracy and making it challenging to assess its performance accurately. Instead, we used a series of thresholds to gradually remove sequences from the test set that were homologous to the training set. For artificial contigs with less than 30% sequence homology against the training set, IPEV achieved an average AUC of 0.98, indicating that our model learned valuable information and did not rely solely on sequence similarity for prediction. Our results suggest that IPEV can generalize well and provide reliable predictions for prokaryotic and eukaryotic virus-derived sequence fragments.

Our objective was to analyze the neural network by examining its probability scores for phage prediction. The presence of the RBP is crucial for the specific interaction between the bacteriophage and its host. Therefore, we specifically focused on genes manual confirmation that are associated with RBPs, as well as an equal number of non-RBP genes. Upon comparing the score distribution of IPEV between the two datasets, we observed a notable difference. The former dataset exhibited a higher score distribution in IPEV. This discrepancy suggests that RBPs with host information could substantially influence phage prediction within the IPEV framework. As the neural network model learns

from well-annotated data, IPEV can also uncover valuable insights regarding the virus-host relationship. This contributes to our overall understanding of neural networks and their performance in relevant tasks.

We utilized the IPEV approach to conduct a comprehensive analysis of longitudinal data from the gut virome, which includes eukaryotic viruses primarily acquired from the environment and diet as well as prokaryotic viruses that target microorganisms [50]. Our investigation revealed that the phage community exhibits remarkable resilience to environmental disturbances, with the “kill the winner” theory having a minimal impact at a higher taxonomic level. This finding aligns with previous research and supports the hypothesis that the “kill the winner” theory fosters a more diverse species community, ultimately enhancing ecosystem resilience. Furthermore, we observed significant fluctuations in the abundance of PPV, primarily driven by phages that specifically target representative gut microbiota.

Although IPEV was initially designed for identifying short viral sequences, our study also encompassed an evaluation over extended lengths of 3,000 to 5,000 bp. And this length spectrum allowed us to include vConTACT v.2.0 and iPHoP in our assessment. We constructed three datasets with contig lengths of 3,000–5,000 bp (details on dataset construction and evaluation methods are provided in the Supplementary Materials and Methods). Our findings indicate that IPEV consistently outperformed the others, achieving an average F1-score of 0.99 as depicted in Figure S11. This score significantly exceeds the F1-scores of 0.02 for vConTACT v.2.0 ($t = -31.29$, $P_{adj} < 0.0001$, two-tailed independent t-tests using Benjamini-Hochberg adjustment) and 0.41 for iPHoP ($t = -395.63$, $P_{adj} < 0.0001$, two-tailed independent t-tests), respectively.

Moreover, to fulfill the demands of high-throughput data processing, we implemented a design for IPEV that enhances the efficiency of analyzing large datasets. IPEV optimizes processing time by returning all taxon prediction scores in a single iteration, requiring only four loads of the neural network weight. Unlike HTP, which expends processing time on file I/O operations, this approach allows IPEV to handle large datasets more effectively. This substantial reduction in computational overhead significantly improves the running speed of IPEV. As shown in Figure S2, IPEV operates 50 times faster than HTP, 30 times faster than vConTACT v.2.0, and 1,225 times faster than iPHoP, taking only 9.6 minutes to analyze 20,000 sequences of 1,200-1,800 bp when using the same computational resources (CPU: Intel® Xeon® 20 cores, GPU: NVIDIA Tesla V100).

An important consideration when using IPEV is the potential bacterial and fungal contamination in virome datasets [60, 61]. To address this, we designed a feature within IPEV that eliminates false-

positive non-viral components (bacteria and fungi). This feature is available as an optional switch. Our current tool has limitations as it does not encompass downstream virus classification or provide specific virus-host predictions for virome data. A more precise and systematic classification system is required to understand the influence viruses have on microbial communities and hosts. We eagerly anticipate the development of additional tools that will reduce the extent of virome dark matter and enhance our comprehension of the virome's intricacies.

**Availability of supporting source code and requirements**

Project name: IPEV

Project home page: https://github.com/basehc/IPEV.

Operating system: IPEV is platform-independent.

Programming language: Python

Other requirements: IPEV is built on Python 3.8.6 and Tensorflow 2.3.1.

License: The source code of IPEV is distributed as open source under the GNU GPL V3.

RRID:SCR_023702

BioTools ID: IPEV

**Availability of data and materials**

Our study contains only publicly available viral genome sequences, reference bacterial genome sequences, the IPEV program, and a detailed tutorial that can be publicly available at Zhu Lab home page (https://cqb.pku.edu.cn/zhulab/info/1006/1156.htm) or GitHub (https://github.com/basehc/IPEV) under GNU General Public License V3. Code, data and Docker image for transparent and reproducible results were documented on Zenodo (https://doi.org/10.5281/zenodo.10118192).

**Additional files**

Supplementary Materials for this manuscript include the following:

Table S1: Details the distribution of contigs across various length ranges within a 5-fold cross-validation dataset based on Virus-Host DB.

Table S2: Number of simulated contigs based on RVDB and IMG/VR v4 databases.

Table S3: The average performance of IPEV and HTP (KNN) with 5-fold cross-validation for various sequence lengths, expressed as a percentage.

Table S4: The average AUC value for four length groups on a 5-fold cross-validation.

Table S5: Enumerates the numbers of the prokaryotic virus contigs in Dataset I1-I6.

Table S6: Enumerates the numbers of the eukaryotic virus contigs in Dataset I1-I6.

Table S7-S12: Compare the performance of IPEV and HTP under various query parameters for coverage and identity in Dataset I1-I6.

Table S13: Performance of IPEV and HTP on the ducks' gut virome data a total of 682 eukaryotic contigs and 1453 prokaryotic virus contigs).

Table S14: Likelihood scores predicted by IPEV algorithm on capping enzymes. And Table S15: P-values show the phageome's average coefficient of variation was significantly lower compared to the PPV (Wilcoxon rank-sum test, adjusted using the Benjamini-Hochberg correction).

Figure S1: The ROC curves and AUC value of IPEV and HTP performances in each set of 5-fold cross-validation.

Figure S2: Performance of IPEV, HTP, vConTACT2, and iPHoP as the number of sequences increases under the same computing configuration (1,200-1,800 bp).

Figure S3-S7: Comparison of IPEV and HTP on the Dataset I2-I6.

Figure S8: Mean accuracy, mean loss, and mean of IPEV's 5-cross-validation on Group A.

Figure S9: The mean validation loss and accuracy of IPEV's 5-cross-validation on Group A.

Figure S10: A. Performance (accuracy, specificity, sensitivity, precision, and F1-score) of IPEV and HTP (KNN, SVC, LR, QDA) on the duck gut virome data. B. The ROC curve and AUC value of IPEV and HTP (KNN, SVC, LR, QDA) on the duck gut virome data.

Figure S11. Average performance of IPEV, HTP, iPHoP, and vConTACT v.2.0 across three independent test sets with sequence lengths of 3,000-5,000 bp and Figure S12. the confusion matrices show the false positive reduction capability of IPEV on datasets with a 1:1 ratio of viruses to non-viruses across Groups A to D.

**Author contributions statements**

H.Q.Z., H.C.Y. and S.F.W. conceived and designed the project; H.C.Y., J.T. and S.F.W. constructed the datasets; H.C.Y. and S.F.W. wrote and optimized the model of IPEV; H.C.Y. and S.F.W. performed the data analysis and design of the pipeline and prepared all the figures and tables; H.C.Y. and H.Q.Z. drafted the manuscript. H.Q.Z., H.C.Y., S.F.W., Q.G., M.L., J.Y.G., Y.Q. W., X.Q.J., and J.T. revised and edited the manuscript, and all authors proofread and improved the manuscript.

## Acknowledgments

This work was supported by the National Key Research and Development Program of China (2021YFC2300300) and the National Natural Science Foundation of China (32070667, 31671366, 32300078). Part of the analysis was performed on the High-Performance Computing Platform of Peking University. We thank Tianze Wang of Peking University for helpful discussions

## Conflict of interest

The authors declare that there is no conflict of interest.

## Tables and Figures Legends

**Figure 1.** Workflow for extracting the sequence pattern matrix and using a deep learning neural network structure to predict taxon. **A.** The virus genomes are initially divided into five subsets, and then each subset is simulated to represent four groups with different contig lengths. **B.** Overlapping trinucleotides are used to represent the virus contigs. For example, if the nucleotides of the viral fragment are “ATTCATAACTT”, the trinucleotide set would consist of “ATT, TTC, TCA, CAT, ATA, TAA, AAC, ACT, CTT”. The trinucleotide set is then converted to a 64x64 sequence pattern matrix using a sequence pattern function. **C.** The IPEV tool employs a 2D CNN model as the classifier. The CNN model accepts the sequence pattern matrix as input and outputs a 1x2 array representing the likelihood of prokaryotic and eukaryotic viruses.

**Figure 2.** Panels A, B, C, and D display the comparative performance of IPEV and HTP (KNN, SVC, LR, and QDA) with 5-fold cross-validation across Groups A, B, C, and D, respectively. * Sn = TP / (TP + FN), Sp = TN / (TN + FP), ACC = (TP + TN) / (TP + TN + FP + FN), Precision = TP / (TP + FP), F1-score = 2 × Precision × Recall / (Precision + Recall), where TP, TN, FP, and FN respectively represent true positive, true negative, false positive, and false negative. As the method with the best performance in HTP, KNN is selected for comparison. The mean and standard deviation of 5-fold cross-validation are computed to elaborate on performance evaluation. Due to a lack of reconstruction between the train and validation set, the performance of HTP (KNN) is overestimated. (In this paper, prokaryotic viruses are treated as positive samples.)

**Figure 3.** The Average performance of IPEV and HTP (KNN) with 5-fold cross-validation for various sequence lengths, expressed as a percentage.

**Figure 4.** Panels A, B, C, and D display the comparative performance of IPEV and HTP (KNN) across Groups A, B, C, and D, respectively, of Dataset I1. (parameter: query coverage = 30%, identity = 30%).

**Figure 5.** Performance comparison between IPEV and HTP (KNN) on Dataset I1. A. The ROC (Receiver operating characteristic) curve demonstrates the discrimination capability, particularly in class-balanced test sets, with higher

AUC values preferred. B. The Precision-Recall curves measure discrimination capability in class-imbalanced test sets, with AP representing the Average Precision.

**Table 1.** Comparison of IPEV and HTP's AUC value on artificial datasets (1,200-1,800 bp) with varying error rates.

**Figure 6.** Histogram illustrating the predicted likelihood scores generated by IPEV for receptor-binding proteins (RBPs) and non-RBPs.

**Figure 7. A.** Box plots representing AUC scores of the ROC curves for IPEV, KNN, SVC, LR, and QDA. **B.** Violin plots displaying the AUC scores differences of each tool relative to IPEV.

**Figure 8. A.** Box plots representing the AUC scores of ROC curves for IPEV, KNN, SVC, LR, and QDA. **B.** Violin plots displaying the AUC scores differences of each tool relative to IPEV. **C.** Relative abundances of phages, PPV, and PPV-associated phages in the longitudinal data of subjects 916-925 as determined by IPEV (Details of the annotations can be found in the Supplementary Materials and Methods).

## References


1. Mushegian AR. Are There $10^{31}$ Virus Particles on Earth, or More, or Fewer?, *J. Bacteriol.* 2020;202(9) :e00052–20..
2. Mya B, Forest R. Here a virus, there a virus, everywhere the same virus?, *Trends Microbiol.* 2005;13(6):278-84.
3. Carding SR, Davis N, Hoyles L. Review article: the human intestinal virome in health and disease, *Aliment. Pharmacol. Ther.* 2017;46(9):800-15.
4. Reyes A, Semenkovich NP, Whiteson K et al. Going viral: next-generation sequencing applied to phage populations in the human gut, *Nat. Rev. Microbiol.* 2012;10(9):607-17.
5. Santiago-Rodriguez TM, Hollister EB. Human Virome and Disease: High-Throughput Sequencing for Virus Discovery, Identification of Phage-Bacteria Dysbiosis and Development of Therapeutic Approaches with Emphasis on the Human Gut, *Viruses.* 2019;11(7):656.
6. Mandal RS, Saha S, Das S. Metagenomic surveys of gut microbiota, *Genomics Proteomics Bioinf.* 2015;13(3):148-58.
7. Lim ES, Zhou Y, Zhao G et al. Early life dynamics of the human gut virome and bacterial microbiome in infants, *Nat. Med.* 2015;21(10):1228-34.
8. Edwards RA, McNair K, Faust K et al. Computational approaches to predict bacteriophage–host relationships, *FEMS Microbiol. Rev.* 2016;40(2):258-72.

9. Wu S, Fang Z, Tan J et al. DeePhage: distinguishing virulent and temperate phage-derived sequences in metavirome data with a deep learning approach, *GigaScience.* 2021;10(9):giab056.

10. Hall AJ. Noroviruses: The Perfect Human Pathogens?, *The Journal of Infectious Diseases.* 2012;205(11):1622-24.

11. Anderson EJ, Weber SG. Rotavirus infection in adults, *Lancet Infect. Dis.* 2004;4(2):91-99.

12. Bosch A, Pintó RM, Guix S. Human Astroviruses, *Clin. Microbiol. Rev.* 2014;27(4):1048-74.

13. Feng Z, Hensley L, McKnight KL et al. A pathogenic picornavirus acquires an envelope by hijacking cellular membranes, *Nature.* 2013;496(7445):367-71.

14. Ghebremedhin B. Human adenovirus: Viral pathogen with increasing importance, *Eur J Microbiol Immunol.* 2014;4(1):26-33.

15. Pfeiffer JK, Virgin HW. Viral immunity. Transkingdom control of viral infection and immunity in the mammalian intestine, *Science.* 2016;351(6270):aad5872.

16. Conceição-Neto N, Deboutte W, Dierckx T et al. Low eukaryotic viral richness is associated with faecal microbiota transplantation success in patients with UC, *Gut.* 2018;67(8):1558-59.

17. Jones MK, Watanabe M, Zhu S et al. Enteric bacteria promote human and mouse norovirus infection of B cells, *Science.* 2014;346(6210):755-9.

18. Metzger RN, Krug AB, Eisenacher K. Enteric Virome Sensing-Its Role in Intestinal Homeostasis and Immunity, *Viruses.* 2018;10(4):146.

19. Rose R, Constantinides B, Tapinos A et al. Challenges in the analysis of viral metagenomes, *Virus Evol.* 2016;2(2):vew022.

20. Jenkins C, Ling CL, Ciesielczuk HL et al. Detection and identification of bacteria in clinical samples by 16S rRNA gene sequencing: comparison of two different approaches in clinical practice, *J. Med. Microbiol.* 2012;61(Pt 4):483-88.

21. Federhen S. The NCBI Taxonomy database, *Nucleic Acids Res.* 2011;40(D1):D136-D43.

22. Yuan D, Ahamed A, Burgin J et al. The European Nucleotide Archive in 2023, *Nucleic Acids Res.* 2023; gkad1067.

23. Krishnamurthy SR, Wang D. Origins and challenges of viral dark matter, *Virus Res.* 2017;239:136-42.

24. Tan J, Fang Z, Wu S et al. HoPhage: an ab initio tool for identifying hosts of phage fragments from metaviromes, *Bioinformatics.* 2021;38(2):543-45.

25. Roux S, Camargo AP, Coutinho FH et al. iPHoP: An integrated machine learning framework to maximize host prediction for metagenome-derived viruses of archaea and bacteria, *PLoS Biol.* 2023;21(4):e3002083.

26. Coutinho FH, Zaragoza-Solas A, Lopez-Perez M et al. RaFAH: Host prediction for viruses of Bacteria and Archaea based on protein content, *Patterns (N Y).* 2021;2(7):100274.

27. Galiez C, Siebert M, Enault F et al. WIsH: who is the host? Predicting prokaryotic hosts from metagenomic phage contigs, *Bioinformatics.* 2017;33(19):3113-14.

28. Shang J, Sun Y. CHERRY: a Computational metHod for accuratE pRediction of virus–pRokarYotic interactions using a graph encoder–decoder model, *Briefings Bioinf.* 2022; *23*(5), bbac182.

29. Lu C, Zhang Z, Cai Z et al. Prokaryotic virus host predictor: a Gaussian model for host prediction of prokaryotic viruses in metagenomics, *BMC Biol.* 2021;19(1):5.

30. Wang W, Ren J, Tang K et al. A network-based integrated framework for predicting virus–prokaryote interactions, *NAR: Genomics Bioinf.* 2020;2(2).p.lqaa044

31. McNair K, Bailey BA, Edwards RA. PHACTS, a computational approach to classifying the lifestyle of phages, *Bioinformatics.* 2012;28(5):614-8.

32. Song K. Classifying the Lifestyle of Metagenomically-Derived Phages Sequences Using Alignment-Free Methods, *Frontiers in Microbiology.* 2020;11:567769.

33. Fang Z, Tan J, Wu S et al. PPR-Meta: a tool for identifying phages and plasmids from metagenomic fragments using deep learning, *GigaScience.* 2019;8(6).p.giz066

34. Ren J, Song K, Deng C et al. Identifying viruses from metagenomic data using deep learning, *Quant Biol.* 2020;8(1):64-77.

35. Kieft K, Zhou Z, Anantharaman K. VIBRANT: automated recovery, annotation and curation of microbial viruses, and evaluation of viral community function from genomic sequences, *Microbiome.* 2020;8(1):90.1-23

36. Bin Jang H, Bolduc B, Zablocki O et al. Taxonomic assignment of uncultivated prokaryotic virus genomes is enabled by gene-sharing networks, *Nat Biotechnol.* 2019;37(6):632-39.

37. Roux S, Enault F, Hurwitz BL et al. VirSorter: mining viral signal from microbial genomic data, *PeerJ.* 2015;3:e985.

38. Guo J, Bolduc B, Zayed AA et al. VirSorter2: a multi-classifier, expert-guided approach to detect diverse DNA and RNA viruses, *Microbiome.* 2021;9(1):37.1-13

39. Wang D. 5 challenges in understanding the role of the virome in health and disease, *PLoS Pathog.* 2020;16(3):e1008318.

40. Galan W, Bak M, Jakubowska M. Host Taxon Predictor - A Tool for Predicting Taxon of the Host of a Newly Discovered Virus, *Sci. Rep.* 2019;9(1):3436.

41. Greninger AL. A decade of RNA virus metagenomics is (not) enough, *Virus Res.* 2018;244:218-29.

42. Mihara T, Nishimura Y, Shimizu Y et al. Linking Virus Genomes with Host Taxonomy, *Viruses.* 2016;8(3):66.

43. Schoch CL, Ciufo S, Domrachev M et al. NCBI Taxonomy: a comprehensive update on curation, resources and tools, *Database (Oxford).* 2020; baaa062.

44. Goodacre N, Aljanahi A, Nandakumar S et al. A Reference Viral Database (RVDB) To Enhance Bioinformatics Analysis of High-Throughput Sequencing for Novel Virus Detection, *mSphere.* 2018;3(2):10-1128

45. Camargo AP, Nayfach S, Chen IA et al. IMG/VR v4: an expanded database of uncultivated virus genomes within a framework of extensive functional, taxonomic, and ecological metadata, *Nucleic Acids Res.* 2023;51(D1):D733-D43.

46. Richter DC, Ott F, Auch AF et al. MetaSim—A Sequencing Simulator for Genomics and Metagenomics, *PloS One.* 2008;3(10):e3373.

47. Jais PH, Decroly E, Jacquet E et al. C3P3-G1: first generation of a eukaryotic artificial cytoplasmic expression system, *Nucleic Acids Res.* 2019;47(5):2681-98.

48. Rangel-Pineros G, Almeida A, Beracochea M et al. VIRify: an integrated detection, annotation and taxonomic classification pipeline using virus-specific protein profile hidden Markov models, *PLoS Comput. Biol.* 2023;19(8):e1011422.

49. Gregory AC, Zayed AA, Conceição-Neto N et al. Marine DNA viral macro-and microdiversity from pole to pole, *Cell.* 2019;177(5):1109-23. e14.

50. Shkoporov AN, Clooney AG, Sutton TDS et al. The Human Gut Virome Is Highly Diverse, Stable, and Individual Specific, *Cell Host  Microbe.* 2019;26(4):527-41.e5.

51. Bankevich A, Nurk S, Antipov D et al. SPAdes: a new genome assembly algorithm and its applications to single-cell sequencing, *J. Comput. Biol.* 2012;19(5):455-77.
52. Ranjan C, Ebrahimi S, Paynabar KJapa. Sequence graph transform (SGT): A feature extraction function for sequence data mining, *Data Mining and Knowledge Discovery* 2022;36(2):668-708.
53. Mock F, Viehweger A, Barth E et al. VIDHOP, viral host prediction with deep learning, *Bioinformatics.* 2020;37(3):318-25.
54. Bahir I, Fromer M, Prat Y et al. Viral adaptation to host: a proteome‐based analysis of codon usage and amino acid preferences, *Mol. Syst. Biol.* 2009;5(1):311.
55. Dohm JC, Peters P, Stralis-Pavese N et al. Benchmarking of long-read correction methods, *NAR: Genomics Bioinf.* 2020;2(2):lqaa037.
56. Pourmohammadi R, Abouei J, Anpalagan A. Error analysis of the PacBio sequencing CCS reads, *The International Journal of Biostatistics.* 2023.
57. Roux S, Hallam SJ, Woyke T et al. Viral dark matter and virus-host interactions resolved from publicly available microbial genomes, *elife.* 2015;4:e08490.
58. Garmaeva S, Gulyaeva A, Sinha T et al. Stability of the human gut virome and effect of gluten-free diet, *Cell Rep.* 2021;35(7):109132.
59. Ren J, Ahlgren NA, Lu YY et al. VirFinder: a novel k-mer based tool for identifying viral sequences from assembled metagenomic data, *Microbiome.* 2017;5(1):69.
60. Zhang F, Zuo T, Yeoh YK et al. Longitudinal dynamics of gut bacteriome, mycobiome and virome after fecal microbiota transplantation in graft-versus-host disease, *Nat. Commun.* 2021;12(1):65.
61. Zolfo M, Pinto F, Asnicar F et al. Detecting contamination in viromes using ViromeQC, *Nat. Biotechnol.* 2019;37(12):1408-12.

 Click here to access/download;Figure;Figure_1.jpg

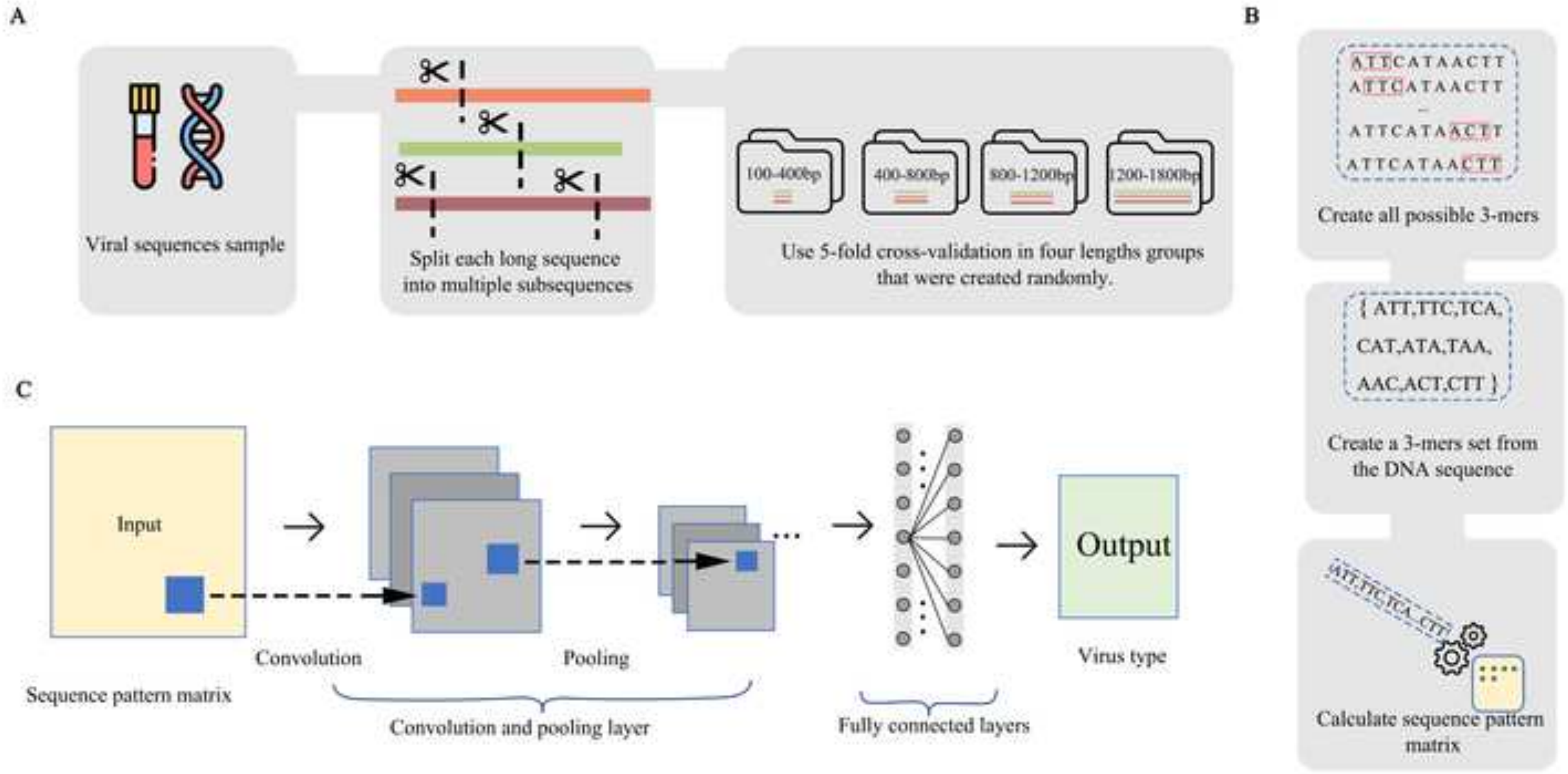

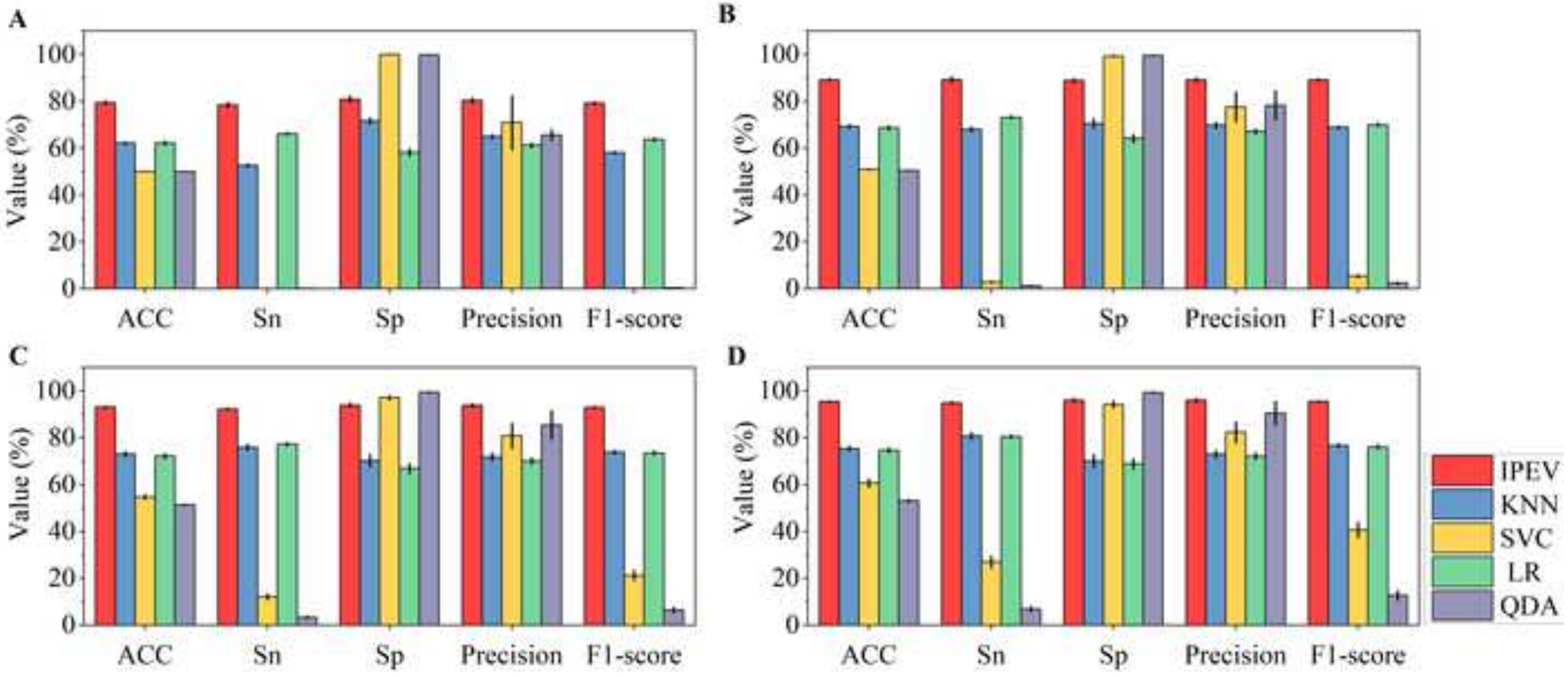
A
B
C
D
Value (%)
0
20
40
60
80
100
ACC
Sn
Sp
Precision
F1-score
IPEV
KNN
SVC
LR
QDA

Figure_3

Click here to access/download;Figure;Figure_3.jpg

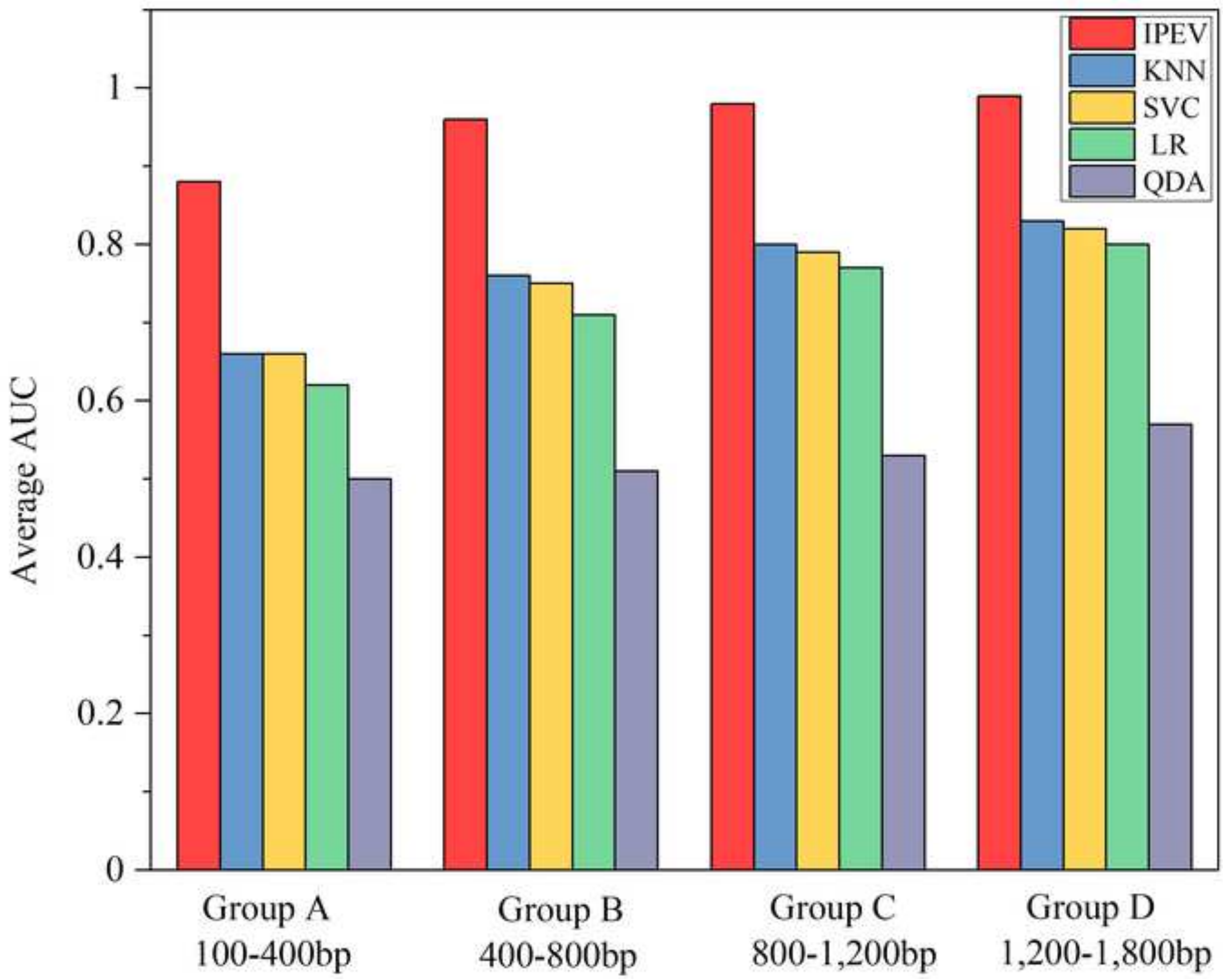

Figure_4_revised

Click here to access/download;Figure;Figure_4.jpg

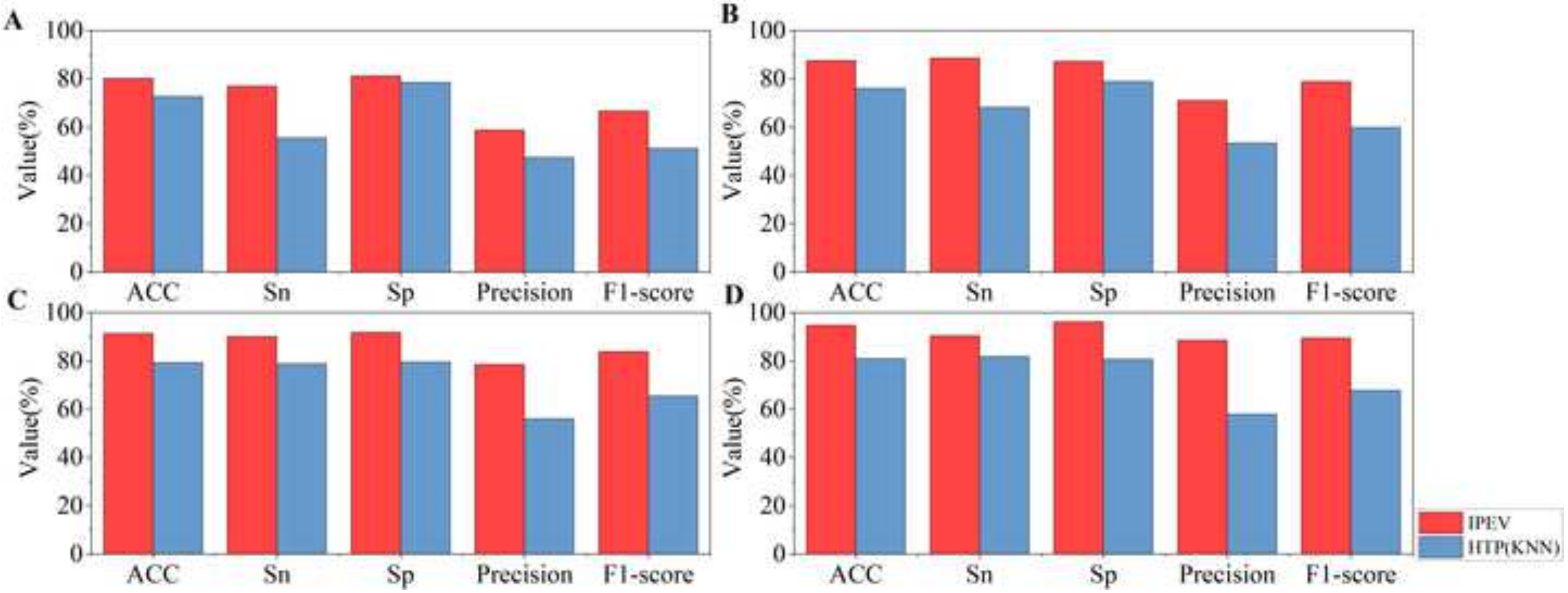

Figure_5 Click here to access/download;Figure;Figure_5.jpg

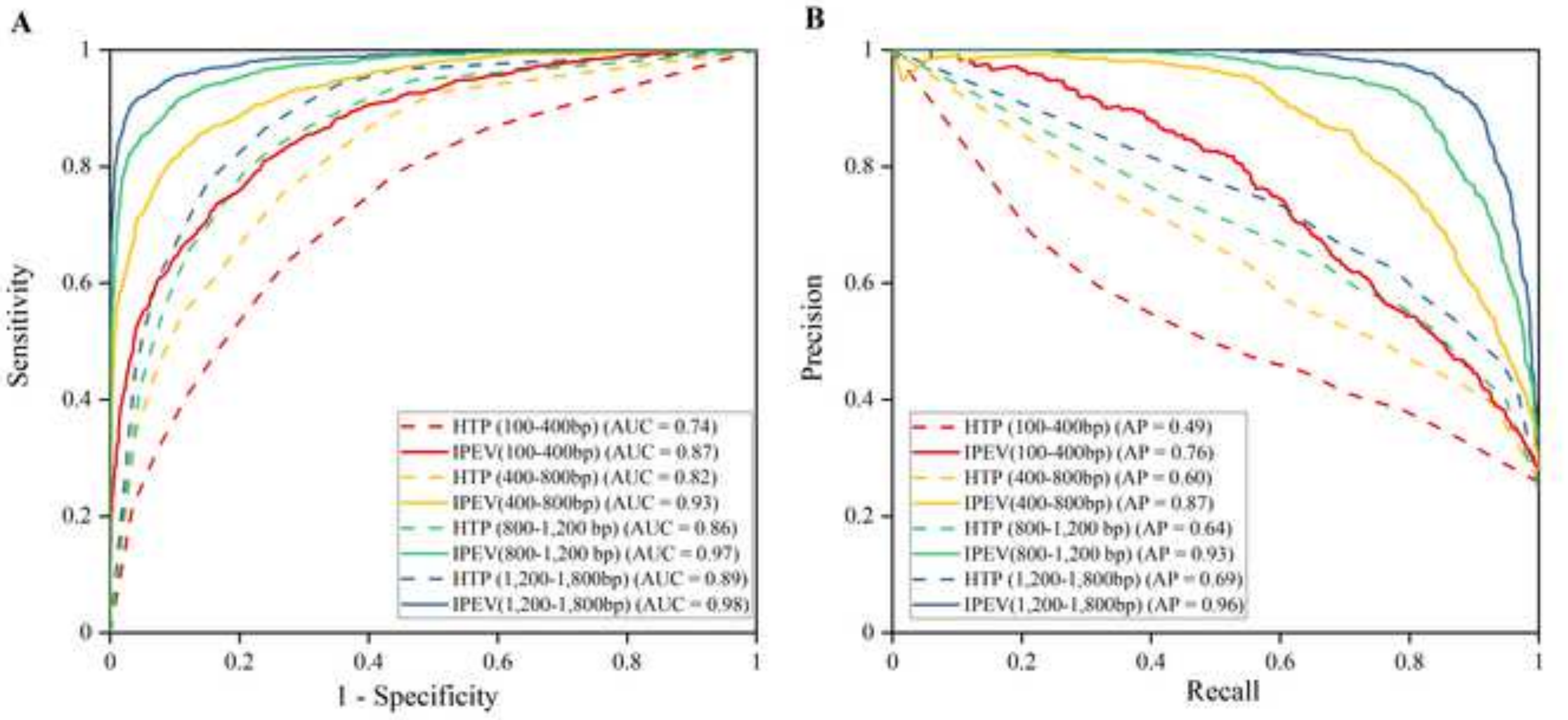

Figure_6

Click here to access/download;Figure;Figure_6.jpg

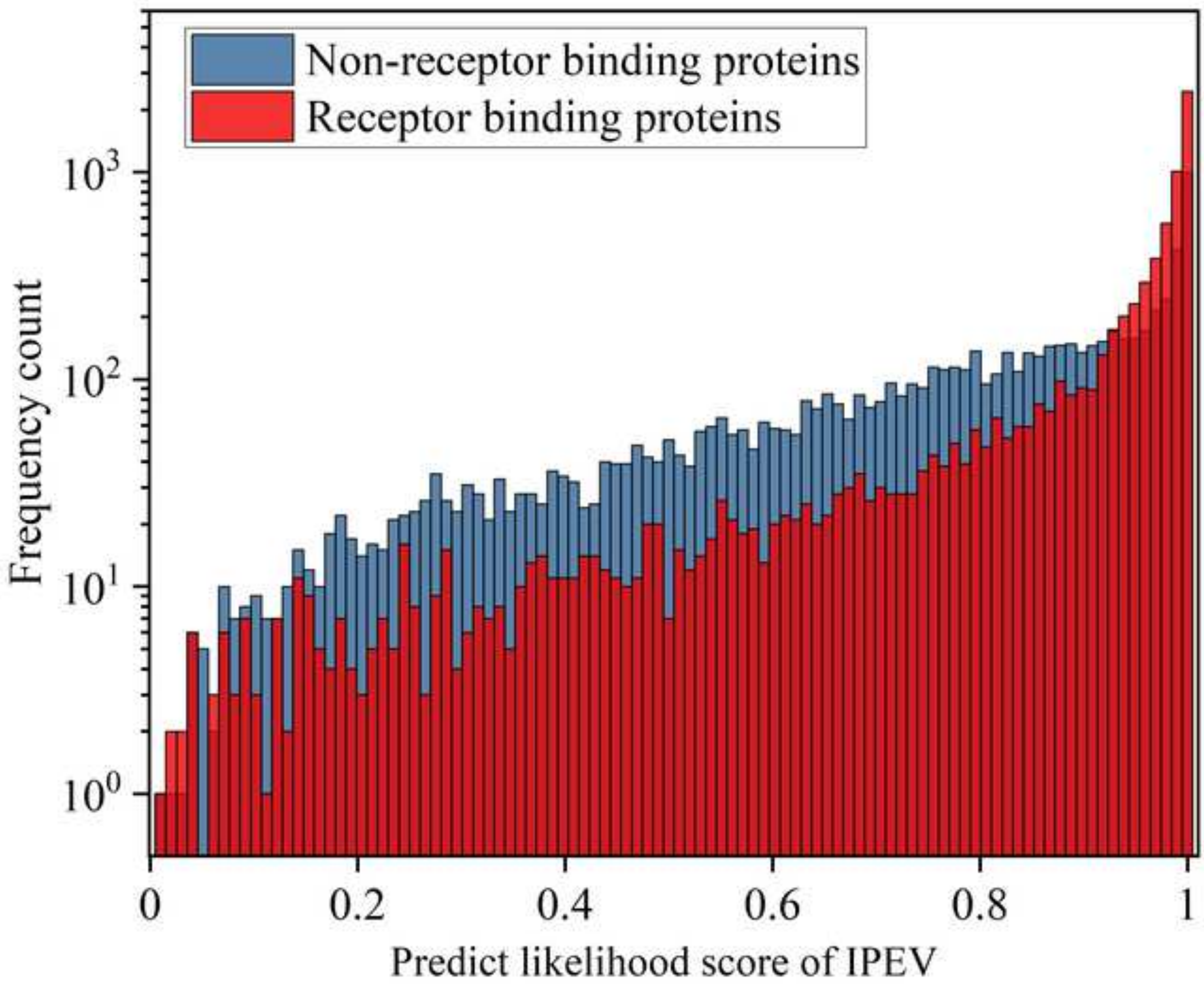

 Click here to access/download;Figure;Figure_7.jpg

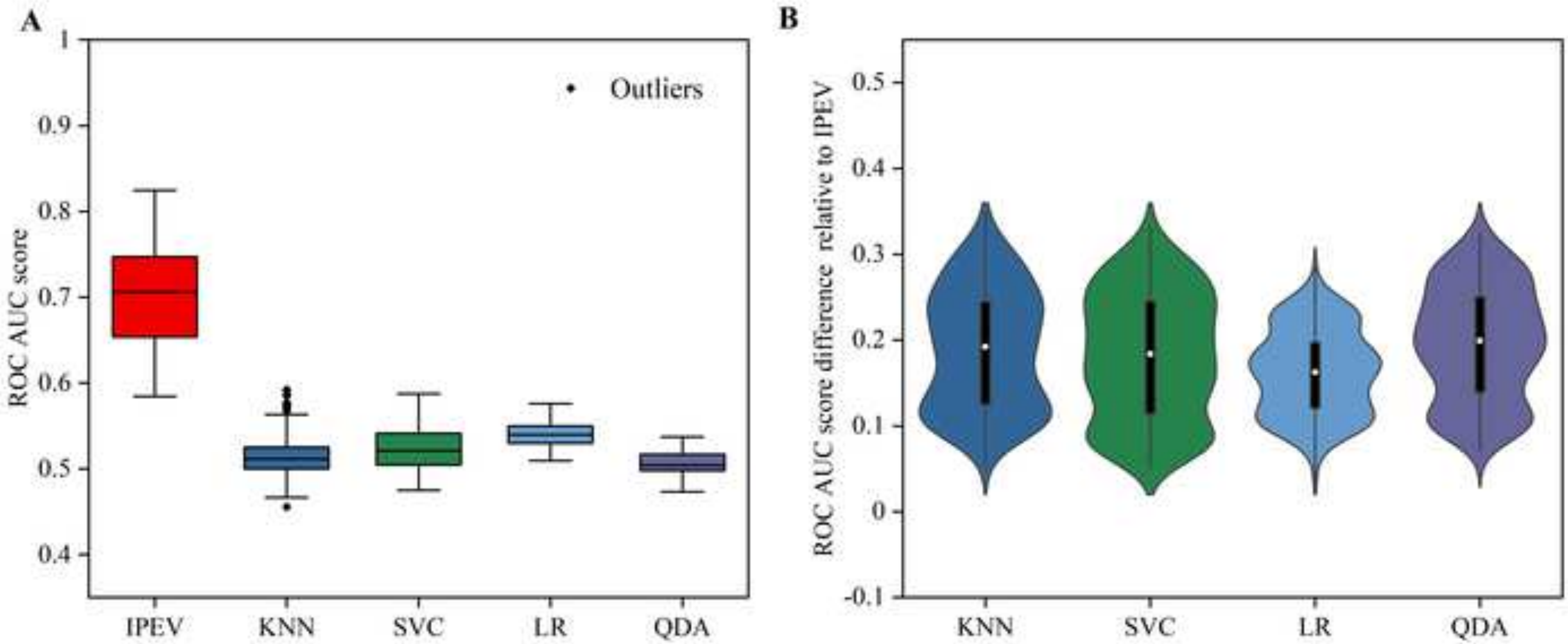

Click here to access/download;Figure;Figure_8.jpg

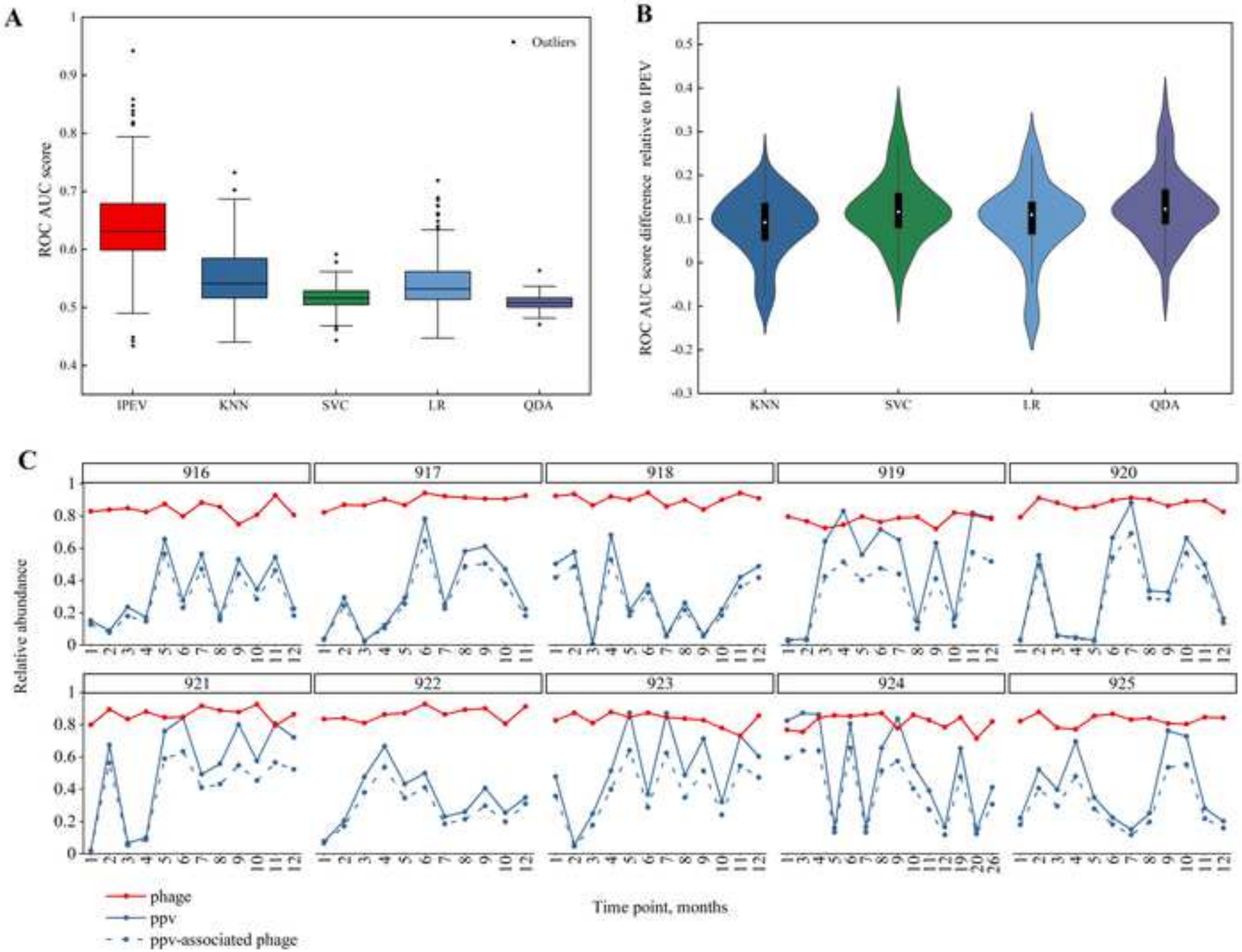

Click here to access/download
**Supplementary Material**
IPEV_R2_Supplementary_Materials.docx

Personal Cover
Click here to access/download;Personal Cover;Cover Letter_revised.docx

Dear Editor,

Thank you very much for your previous Email on March 9, 2024, regarding our manuscript, "IPEV: identification of prokaryotic and eukaryotic virus-derived sequences in virome using deep learning" (GIGA-D-23-00357). We are very pleased to learn that our manuscript is acceptable for publication in GigaScience, subject to the completion of revisions suggested by the reviewer. Herein, we would like to express our gratitude to you, and the expert member of the Editorial Board, and Reviewer 1, for your thorough review. We are also pleased to note that our responses have addressed all of their concerns, that the "expert and the previous referee agree that the revisions are satisfactory". Additionally, we are grateful for their careful reading of the previous version of our manuscript, and the further comments raised by the reviewer helped us make further improvements.

Following your suggestion, we have registered our new software application in the bio.tools database to receive a BioTools ID (biotools:IPEV), and we have included this ID in the latest version of the manuscript following the RRID.

In this revision, we made several minor changes (reported below) exactly followed Reviewer 1's and your points. In addition, we have carefully reviewed the journal's style and ensured that the revised manuscript conforms to it. At this stage, we have removed the tracking of changes or highlighting in red that was made for the purpose of peer review in the revised manuscript. We then report our revisions and point-by-point responses to Reviewer 1's comments (in *italic text*) one by one as follows:

***Reviewer 1：***

***Comment 1:*** *The authors include some text in the Discussion section (paragraph from line 423 to line 436, and paragraph from line 437 to 448) that, in my opinion, would fit better in the Results section. I suggest the authors include these in the Results section, and then in the Discussion comment how those results compare to other methods and what are their implications.*

We are deeply grateful for Reviewer 1's suggestions. Following the suggestion of Review 1, we have relocated the below sentences into Subsection "Performance on viral genome fragments using cross-validation" as follows:

"In addition, we plotted the training loss, validation loss, training accuracy, and validation accuracy

curves with respect to the number of epochs using 5-fold cross-validation. We observed that the model converged at 30 epochs, with training and validation losses and accuracies remaining consistent and overlapping. This indicates that the model achieved high performance while avoiding overfitting or underfitting the data, as shown in Figures S8 and S9". (Please refer to Line 232-236, Page 8 in the revised manuscript.)

"We also test IPEV's effectiveness in differentiating viral from non-viral genome fragments in datasets where viruses and non-viruses are present in a 50:50 ratio. As shown in Figure S12, across Groups A to D, IPEV achieved Sn scores of 0.73, 0.833, 0.905, and 0.931, respectively (details on sample construction and methods can be found in Supplementary Materials and Methods)." (Please refer to Line 236-240, Page 8 in the revised manuscript.)

***Comment 2:*** *I would suggest modifying the sentence in line 42 like this: "Nonetheless, it is essential to note that enriched sample approaches carry the risk of losing valuable host or environmental information [8], potentially leading to inaccurate virus host identification and constraining subsequent analyses."*

We are very thankful for Reviewer 1's comment. We have revised the sentence "Nonetheless, it is essential to note that enriched sample approaches carry the risk of losing valuable host or environmental information [8] and can cause inaccurate virus host identification, thereby constraining subsequent analyses." as "Nonetheless, it is essential to note that enriched sample approaches carry the risk of losing valuable host or environmental information [8], potentially leading to inaccurate virus host identification and constraining subsequent analyses." (Please refer to Line 42-44, Page 2 in the revised manuscript.)

***Comment 3:*** *In the sentence starting in line 392, instead of "During" use "For".*

We are very thankful for Reviewer 1's comment. We have replaced "During" with "For". Therefore, we have revised "During artificial contigs with less than 30% sequence homology against the training set" as "For artificial contigs with less than 30% sequence homology against the training set." (Please refer to Line 192, Page 14 in the revised manuscript.)

In hoping that the above revision has clarified all the points by Reviewer 1 and given a point-

by-point response to all the concerns, we hereby resubmit our manuscript to the journal. We thank you for your kind consideration. Please do not hesitate to let us know if additional edits are needed. We will be no more than willing to meet your requirements.

Sincerely yours,

Huaiqiu Zhu, Ph. D., Professor

Peking University
