# Supplementary material for: IPEV: identification of prokaryotic and eukaryotic virus-derived sequences in virome using deep learning
Source: Gigascience. 2024 Apr 22;13:giae018. doi: 10.1093/gigascience/giae018 (PMC11034026; doi:10.1093/gigascience/giae018)
Supplement: giae018_Supplemental_File [file giae018_supplemental_file.docx]

**Supplementary Materials for**

IPEV: identification of the prokaryotic and eukaryotic virus-derived sequences in virome using deep learning

Hengchuang Yin^1^, Shufang Wu^1^, Jie Tan^1^, Qian Guo^1^, Mo Li^1,2^, Jinyuan Guo^1,3^, Yaqi Wang^1^, Xiaoqing Jiang^1,4^ and Huaiqiu Zhu^1,2,3^*

*^1^ Department of Biomedical Engineering,* *College of Future Technology, and Center for Quantitative Biology, Peking University, Beijing 100871, Beijing, China;*

*^2^ School of Life Sciences, Peking University, Beijing 100871, Beijing, China;*

*^3^ Department of Biomedical Engineering, Georgia Institute of Technology and Emory University, GA 30332, Atlanta, USA*

*^4^ Beijing Institute of Genomics, Chinese Academy of Sciences, and China National Center for Bioinformation, Beijing 100101, China*

* To whom correspondence should be addressed. Tel: 8610-6276 7261; Email: hqzhu@pku.edu.cn

**Supplementary Materials for this manuscript include the following:**

Table S1: Details the distribution of contigs across various length ranges within a 5-fold cross-validation dataset based on Virus-Host DB.

Table S2: Number of simulated contigs based on RVDB and IMG/VR v4 databases.

Table S3: The average performance of IPEV and HTP (KNN) with 5-fold cross-validation for various sequence lengths, expressed as a percentage.

Table S4: Provides the average AUC (Area Under the Curve) value for four length groups subjected to 5-fold cross-validation.

Table S5: Enumerates the prokaryotic virus contigs in Dataset I1-I6.

Table S6: Enumerates the eukaryotic virus contigs in Dataset I1-I6.

Table S7-S12: Compare the performance of IPEV and HTP under various query parameters for coverage and identity in Dataset I1-I6.

Table S13: Performance of IPEV and HTP on the ducks’ gut virome data a total of 682 eukaryotic contigs and 1453 prokaryotic virus contigs)

Table S14: Likelihood scores predicted by IPEV algorithm on capping enzymes. And Table S15: P-values show the phageome's average coefficient of variation was significantly lower compared to the PPV (Wilcoxon rank-sum test, adjusted using the Benjamini-Hochberg correction).

Figure S1: The ROC curves and AUC value of IPEV and HTP performances in each set of 5-fold cross-validation.

Figure S2: Performance of IPEV, HTP, vConTACT2, and iPHoP as the number of sequences increases under the same computing configuration (1,200-1,800 bp).

Figure S3-S7: Comparison of IPEV and HTP on Dataset I2-I6.

Figure S8: Mean accuracy, mean loss, and mean of IPEV’s 5-cross-validation on Group A

Figure S9: The mean validation loss and accuracy of IPEV’s 5-cross-validation on Group A.

Figure S10: A. Performance (accuracy, specificity, sensitivity, precision, and F1-score) of IPEV and HTP (KNN, SVC, LR, QDA) on the duck gut virome data. B. The ROC curve and AUC value of IPEV and HTP (KNN, SVC, LR, QDA) on the duck gut virome data.

Figure S11. Average performance of IPEV, HTP, iPHoP, and vConTACT v.2.0 across three independent test sets with sequence lengths of 3,000-5,000 bp and Figure S12. the confusion matrices show the false positive reduction capability of IPEV on Groups A-D in datasets where viruses and non-viruses are present in a 50:50 ratio.

**Supplementary Materials and Methods**

**Independent test sets construction:**

In this study, we evaluated the sensitivity and generalization ability of IPEV to “novel viruses”, using a well-prepared independent test set, which comprised of 1,022 eukaryotic and 1,051 prokaryotic virus sequences. To generate simulated test data, we applied the same MetaSim parameters as previously described to produce 20,000 contigs, ensuring a size ratio of 1:1 between the eukaryotic virus dataset and the prokaryotic virus dataset, for Groups A to D. The independent test set was manually curated by selecting sequence fragments with low similarity against the training set using BLASTn (v2.7.1) search. We specifically ensured that the identity and coverage of sequences in the independent test sets were below a certain threshold z (z < query coverage: x, query i-dentity: y; where x, y = 30%, 60%, 100%) compared to the training set. We generated six low homologies independent test sets (Dataset I1-I6) with varying levels of query coverage and identity relative to the training set. These levels included no more than 30% query coverage and 30% identity, 30% query coverage and 60% identity, 60% query coverage and 30% identity, 60% query coverage and 60% identity, 100% query coverage and 30% identity, and 100% query coverage and 30% identity. Details of each Dataset I1-I6 are provided in Table S3 and Table S4.

**Construction of test set with sequencing errors:**

In this study, we constructed datasets with various sequencing errors, specifically targeting error rates of 5%, 10%, and 15%. These errors were categorized into two types: base substitutions, and base insertions or deletions. for the generation of sequences with a 5% rate of base substitutions, we configured the 'Error Rate at Read Start' and 'Error Rate at End of Read' settings in MetaSim to 0.05. Meanwhile, the 'Insertion Error Rate' and 'Deletion Error Rate' were set to 0, ensuring that only substitution errors were introduced at this stage. To simulate a higher error rate with 10% base insertions or deletions, we modified the settings such that the 'Insertion Error Rate' and 'Deletion Error Rate' were increased to 0.5. This adjustment was crucial for accurately representing datasets with a predominant presence of insertion or deletion errors, while keeping the other parameters consistent with the base substitution scenario.

**Construction of Dataset-2 from RVDB and IMG/VR:**

We accessed IMG/VR v4 on September 15, 2023, through the following link: https://genome.jgi.doe.gov/portal/pages/dynamicOrganismDownload.jsf?organism=IMG_VR. We proceeded to download the high-confidence nucleotide database (IMGVR_all_nucleotides-high_confidence.fna). The “high-confidence” designation refers to the estimated accuracy regarding the completeness and purity of single-scaffold viral genomes, as assessed by CheckV software—a tool utilized by the creators of IMG/VR v4 for qualification purposes. To cluster the sequences, we applied the cd-hit utility, setting the similarity threshold at 100%. The precise command executed was: cd-hit -i $INPUT_FILE -o $OUTPUT_FILE_100 -c 1.0 -n 10 -M 40000 -T 40*.* Following this, we conducted a filtering process on the IMGVR_UViG database, selectively retaining rows where the “Host prediction method” included references to “CRISPR” and “Isolate taxonomy”. Considering that our tool's scope does not encompass proviruses, we excluded them and selected only the high-quality sequences. From these procedures, we get 5,598 prokaryotic sequences.

Secondly, we incorporated data from the Reference Viral Database (RVDB). We accessed RVDB on September 15, 2023, via the website https://rvdb.dbi.udel.edu/ and procured the clustered version of the database v26.0 (released on April 10, 2023), named “C-RVDBv26.0.fasta”. We then clustered the sequences in C-RVDB using cd-hit with the command: cd-hit -i $INPUT_FILE -o $OUTPUT_FILE_100 -c 1.0 -n 10 -M 40000 -T 40. We excluded UNVERIFIED, partial sequences, and REFSEQ entries since we already have the latter in our collection. For each gene, we chose to retain only one strain variant, setting a threshold of 30 for each virus. Through these methods, we obtained 25,644 eukaryotic sequences.

**Construction of extended-length datasets:**

In addition to our viral genome fragment evaluations, we extended our assessment of IPEV’s performance to include viral genome fragments of longer lengths. Specifically, we focused on testing sets ranging from 3,000 to 5,000 base pairs (bp). To achieve a comprehensive evaluation, we manually simulated these sets based on independent test sets, each consisting of an equal number of eukaryotic and prokaryotic virus genomes, totaling 500 contigs from each category. This simulation was repeated three times. This approach was designed to ensure robustness in our results.

**Construction of viral and non-viral datasets**

We designed a comprehensive dataset, creating 4,000,000 sequences for Group A (100-400 bp), 3,600,000 for Group B (400-800 bp), 3,200,000 for Group C (800-1,200 bp), and 2,800,000 for Group D (1,200-1,800 bp), maintaining a 1:1 ratio of viral to non-viral contigs. For the construction of the non-viral component dataset, we performed searches on the NCBI Assembly database using the commands “(Fungi[orgn]) AND (representative genome[filter])” and “(Bacteria[orgn]) AND (representative genome[filter])” to download data sets. We downloaded 1,428 bacterial genome sequences, ensuring to exclude plasmids and include only those labeled as 'complete,' and 99 fungal sequences, excluding macrofungi. We simulated contigs to create negative sample groups: 2 million for Group A (100-400 bp), 1,800,000 for Group B (400-800 bp), 1,600,000 for Group C (800-1,200 bp), and 1,400,000 for Group D (1,200-1,800 bp). Based on the sequence pattern matrix and neural network framework outlined in our manuscript, we retrained, validated, and tested on the constructed Groups A to D using an 8:1:1 split for training, validation, and testing, respectively. Furthermore, we conducted experiments where the non-viral components constituted 50% of the test set. Viruses are treated as the positive sample.

**Deriving viral taxon scores from subsequence predictions**

To evaluate the performance of the neural network models, we carried out extensive training using 5-fold cross-validation within Groups A-D. When provided with a query virus fragment shorter than 1,800 bp, the model from the corresponding length group was used. If the fragment length exceeded 1,800 bp, it was first divided into 1,800 bp windows, and the model from Group D was applied. The remaining subsequences, ranging from 0 to 400 bp, 400 to 800 bp, and 800 to 1,200 bp, were assigned to models from Group A, Group B, and Group C respectively. The final prediction likelihood score was calculated as the weighted average of the scores obtained from each subsequence.

**Annotations and calculation of proportions for PPV and PPV-associated phage within the longitudinal data**

We conducted an analysis of longitudinal data from the study by Shkoporov et al. [1] using the IPEV tool to assess its accuracy and explore the stability of the gut virome. The raw data for a human gut virome dataset were retrieved from the NCBI Sequence Read Archive (SRA) under the accession number PRJNA545408. This dataset included 130 samples from ten healthy adults (subjects 916-925) collected over a 12-month period (T1-T12) through monthly synchronous samplings. The SPAdes software was utilized to assemble sequences, and blastn searches were conducted against a bacterial database to eliminate bacterial sequences with an e-value of e-5, an identity of 50%, and a coverage of 90%. Our bacterial dataset included 20,003 complete prokaryotic genomes sourced from the NCBI RefSeq database, composed of 19,629 bacterial genomes and 374 archaeal genomes.

For the data after removing bacterial sequences, we used IPEV to determine whether sequence fragments originated from eukaryotic viruses or prokaryotic viruses, using a threshold of 0.5. Simultaneously, we discarded all contig sequences less than 450 bp in length. We adhered to Shkoporov's definition of a person's persistent virome (PPV), which includes contigs conserved in ≥6 out of 12 time points. Decontaminated contigs were clustered using cd-hit-est (v.4.8.1) with c 0.8 aS 0.8 d 0 n 5. We defined clusters that contained contigs from at least 6 months as PPV clusters. Since subject 917 was sampled for 11 months, we adjusted the definition of PPV for subject 917 to include contigs that appeared in at least 5 months. We also calculated the proportion of phage annotations in PPV by IPEV, and these phages were defined as PPV-associated phages.

.

**Supplementary Tables**

**Table S1.** Distribution of contigs among different length ranges in a 5-fold cross-validation dataset based on Virus-Host DB

| Group | Total Contigs | Length Range (bp) |
| --- | --- | --- |
| Group A | 1,000,000 | 100-400 |
| Group B | 900,000 | 400-800 |
| Group C | 800,000 | 800-1,200 |
| Group D | 700,000 | 1,200-1,800 |

**Table S2.** Number of simulated contigs based on RVDB and IMG/VR v4 databases.

| Group | Number of prokaryotic virus contigs | Numbers of eukaryotic virus contigs |
| --- | --- | --- |
| Group A  (100-400 bp) | 500,000 | 500,000 |
| Group B  (400-800 bp) | 450,000 | 450,000 |
| Group C  (800-1,200 bp) | 400,000 | 400,000 |
| Group D  (1,200-1,800 bp) | 350,000 | 350,000 |

**Table S3.** The average performance of IPEV and HTP (KNN) with 5-fold cross-validation for various sequence lengths, expressed as a percentage.

| Group | Tool | ACC | Sn | Sp | Precision | F1-score |
| --- | --- | --- | --- | --- | --- | --- |
| Group A | IPEV | 79.4±0.7 | 78.4±0.1 | 80.9±0.1 | 80.3±0.1 | 79.2±0.7 |
|  | HTP | 62.1±0.5 | 52.6±0.5 | 71.6±1.1 | 65.0±0.8 | 58.1±0.5 |
| Group B | IPEV | 89.2±0.5 | 89.3±0.1 | 89.0±0.7 | 89.2±0.4 | 89.2±0.5 |
|  | HTP | 69.3±0.7 | 68.0±1.0 | 70.5±1.7 | 70.0±1.1 | 69.0±0.5 |
| Group C | IPEV | 93.1±0.4 | 92.2±048 | 94.0±0.1 | 93.8±0.1 | 93.0±0.4 |
|  | HTP | 73.1±1.0 | 76.0±1.1 | 70.2±2.3 | 72.0±1.4 | 73.8±0.7 |
| Group D | IPEV | 95.5±0.3 | 95.0±0.5 | 96.1±0.7 | 96.0±0.7 | 95.5±0.3 |
|  | HTP | 75.4±0.9 | 80.8±1.3 | 70.1±0.2 | 73.0±1.4 | 76.6±0.6 |

**Table S4.** The average AUC value of four length group on a 5-fold cross-validation

| Tool | Group A  (100-400 bp) | Group B  (400-800 bp) | Group C  (800-1,200 bp) | Group D  (1,200-1,800 bp) |
| --- | --- | --- | --- | --- |
| IPEV | 0.88 | 0.96 | 0.98 | 0.99 |
| KNN | 0.66 | 0.76 | 0.80 | 0.83 |
| LR | 0.66 | 0.75 | 0.79 | 0.82 |
| SVC | 0.62 | 0.71 | 0.77 | 0.80 |
| QDA | 0.50 | 0.51 | 0.53 | 0.57 |

**Table S5.** The number of the corresponding prokaryotic virus contigs on Dataset I1-I6.

| Dataset | Group A  (100-400 bp) | Group B  (400-800 bp) | Group C  (800-1,200 bp) | Group D  (1,200-1,800 bp) |
| --- | --- | --- | --- | --- |
| I1 | 3,180 | 3,152 | 3,050 | 3,171 |
| I2 | 3,178 | 3,149 | 3,050 | 3,170 |
| I3 | 4,244 | 4,124 | 4,164 | 4,499 |
| I4 | 4,242 | 4,125 | 4,170 | 4,499 |
| I5 | 8,101 | 8,216 | 8,411 | 8,432 |
| I6 | 8,104 | 8,216 | 8,409 | 8,427 |

**Table S6.** The number of the corresponding eukaryotic virus contigs on Dataset I1-I6.

| Dataset | Group A  (100-400 bp) | Group B  (400-800 bp) | Group C  (800-1,200 bp) | Group D  (1,200-1,800 bp) |
| --- | --- | --- | --- | --- |
| I1 | 1,106 | 1,117 | 1,011 | 1,036 |
| I2 | 1,106 | 1,117 | 1,011 | 1,036 |
| I3 | 1,739 | 1,705 | 1,785 | 1,938 |
| I4 | 1,739 | 1,703 | 1,784 | 1,935 |
| I5 | 5356 | 5,591 | 6,097 | 6,069 |
| I6 | 5,359 | 5,595 | 6,082 | 6,065 |

**Table S7.** Comparison of IPEV and HTP (KNN) on Dataset I1 (Query parameter: coverage = 30%, identity = 30%).

| Group | Tool | ACC | Sn | Sp | Precision | F1-score |
| --- | --- | --- | --- | --- | --- | --- |
| Group A | IPEV | 0.8010 | 0.7694 | 0.8119 | 0.5873 | 0.6661 |
|  | HTP | 0.7261 | 0.5552 | 0.7855 | 0.4738 | 0.5112 |
| Group B | IPEV | 0.8754 | 0.8854 | 0.8718 | 0.7100 | 0.7880 |
|  | HTP | 0.7606 | 0.6822 | 0.7884 | 0.5332 | 0.5986 |
| Group C | IPEV | 0.9138 | 0.9001 | 0.9184 | 0.7852 | 0.8387 |
|  | HTP | 0.7929 | 0.7873 | 0.7948 | 0.5598 | 0.6543 |
| Group D | IPEV | 0.9475 | 0.9035 | 0.9618 | 0.8855 | 0.8944 |
|  | HTP | 0.8089 | 0.8176 | 0.8061 | 0.5793 | 0.6781 |

**Table S8.** Comparison of IPEV and HTP on Dataset I2 (Query parameter: coverage=30% identity=60%)

| Group | Tool | ACC | Sp | Sn | Precision | F1-score |
| --- | --- | --- | --- | --- | --- | --- |
| Group A | IPEV | 0.8011 | 0.8118 | 0.7701 | 0.5873 | 0.6664 |
|  | HTP | 0.7264 | 0.7863 | 0.5538 | 0.4741 | 0.5109 |
| Group B | IPEV | 0.8755 | 0.872 | 0.8853 | 0.7103 | 0.7882 |
|  | HTP | 0.7604 | 0.7885 | 0.6810 | 0.5330 | 0.5980 |
| Group C | IPEV | 0.9133 | 0.918 | 0.8992 | 0.7845 | 0.8379 |
|  | HTP | 0.793 | 0.7948 | 0.7875 | 0.5601 | 0.6546 |
| Group D | IPEV | 0.9472 | 0.9618 | 0.9027 | 0.8856 | 0.8941 |
|  | HTP | 0.8092 | 0.8063 | 0.8179 | 0.5803 | 0.6789 |

**Table S9.** Comparison of IPEV and HTP on Dataset I3 (Query parameter: coverage=60% identity=30%)

| Group | Tool | ACC | Sp | Sn | Precision | F1-score |
| --- | --- | --- | --- | --- | --- | --- |
| Group A | IPEV | 0.8100 | 0.8202 | 0.7849 | 0.6414 | 0.7060 |
|  | HTP | 0.7107 | 0.7754 | 0.5526 | 0.5021 | 0.5261 |
| Group B | IPEV | 0.8837 | 0.8790 | 0.8950 | 0.7536 | 0.8182 |
|  | HTP | 0.7475 | 0.7723 | 0.6874 | 0.5552 | 0.6143 |
| Group C | IPEV | 0.9171 | 0.9207 | 0.9087 | 0.8309 | 0.8681 |
|  | HTP | 0.7753 | 0.7719 | 0.7832 | 0.5954 | 0.6765 |
| Group D | IPEV | 0.9529 | 0.9671 | 0.9200 | 0.9234 | 0.9217 |
|  | HTP | 0.7949 | 0.7873 | 0.8127 | 0.6220 | 0.7047 |

**Table S10.** Comparison of IPEV and HTP on Dataset I4 (Query parameter: coverage=60% identity=60%)

| Group | Tool | ACC | Sp | Sn | Precision | F1-score |
| --- | --- | --- | --- | --- | --- | --- |
| Group A | IPEV | 0.8097 | 0.8199 | 0.7849 | 0.6411 | 0.7058 |
|  | HTP | 0.7102 | 0.7749 | 0.5526 | 0.5016 | 0.5259 |
| Group B | IPEV | 0.8837 | 0.8790 | 0.8949 | 0.7533 | 0.8180 |
|  | HTP | 0.7476 | 0.7726 | 0.6870 | 0.5550 | 0.6140 |
| Group C | IPEV | 0.9172 | 0.9209 | 0.9086 | 0.8309 | 0.8680 |
|  | HTP | 0.7749 | 0.7715 | 0.7831 | 0.5945 | 0.6759 |
| Group D | IPEV | 0.9532 | 0.9671 | 0.9209 | 0.9233 | 0.9221 |
|  | HTP | 0.7948 | 0.7873 | 0.8124 | 0.6216 | 0.7043 |

**Table S11.** Comparison of IPEV and HTP on Dataset I5 (Query parameter: coverage=100% identity=30%)

| Group | Tool | ACC | Sp | Sn | Precision | F1-score |
| --- | --- | --- | --- | --- | --- | --- |
| Group A | IPEV | 0.8395 | 0.8383 | 0.8413 | 0.7748 | 0.8067 |
|  | HTP | 0.6879 | 0.7644 | 0.5723 | 0.6162 | 0.5934 |
| Group B | IPEV | 0.9103 | 0.8958 | 0.9315 | 0.8588 | 0.8937 |
|  | HTP | 0.7485 | 0.7712 | 0.7151 | 0.6802 | 0.6972 |
| Group C | IPEV | 0.9418 | 0.9407 | 0.9433 | 0.9202 | 0.9316 |
|  | HTP | 0.7716 | 0.7676 | 0.7773 | 0.7079 | 0.7410 |
| Group D | IPEV | 0.9650 | 0.9746 | 0.9516 | 0.9643 | 0.9579 |
|  | HTP | 0.8002 | 0.7923 | 0.8110 | 0.7376 | 0.7726 |

**Table S12.** Comparison of IPEV and HTP on Dataset I6 (Query parameter: coverage=100% identity=60%)

| Group | Tool | ACC | Sp | Sn | Precision | F1-score |
| --- | --- | --- | --- | --- | --- | --- |
| Group A | IPEV | 0.8395 | 0.8382 | 0.8414 | 0.7747 | 0.8067 |
|  | HTP | 0.6880 | 0.7643 | 0.5727 | 0.6164 | 0.5937 |
| Group B | IPEV | 0.9102 | 0.8959 | 0.9312 | 0.8590 | 0.8937 |
|  | HTP | 0.7484 | 0.7711 | 0.7151 | 0.6802 | 0.6972 |
| Group C | IPEV | 0.9420 | 0.9409 | 0.9434 | 0.9203 | 0.9317 |
|  | HTP | 0.7719 | 0.7676 | 0.7777 | 0.7077 | 0.7410 |
| Group D | IPEV | 0.9649 | 0.9746 | 0.9514 | 0.9642 | 0.9578 |
|  | HTP | 0.7999 | 0.7922 | 0.8106 | 0.7374 | 0.7722 |

**Table S13.** Performance of IPEV and HTP on the ducks’ gut virome data a total of 682 eukaryotic contigs and 1453 prokaryotic virus contigs)

| Tool | ACC | Sp | Sn | Precision | F1-score |
| --- | --- | --- | --- | --- | --- |
| IPEV | 0.7443 | 0.7777 | 0.6730 | 0.5870 | 0.6270 |
| KNN | 0.5822 | 0.5719 | 0.6041 | 0.3985 | 0.4802 |
| SVC | 0.3396 | 0.0365 | 0.9853 | 0.3243 | 0.4880 |
| LR | 0.5925 | 0.6710 | 0.4252 | 0.3776 | 0.0206 |
| QDA | 0.3218 | 0.0034 | 1.0000 | 0.3202 | 0.4851 |

We also evaluate IPEV using real virome data. We compared the performance of IPEV and HTP using the duck gut virome data. The dataset was obtained as raw reads by downloading Mohammed et al.’s sequencing data [2] from the intestinal tract of 23 ducks. We used sequence alignments as the ground truth and set the e-value threshold at 1e-4. After completing the assembly process and quality control using SPAdes, a total of 3,303 contigs were generated. After aligning against the NCBI viral database, 682 eukaryotic viruses and 1,453 phage contigs are utilized because reads are relatively short and have low sequencing depth and coverage. As depicted in Figure S10. IPEV outperformed KNN, SVC, LR, and QDA by 0.14, 0.13, 0.60, and 0.15, respectively. With an F1-score of 0.49 and a specificity of 0.04, SVC and QDA tend to classify eukaryotic viruses as phages, and LR performs poorly in metrics of F1-score. Figure 4B demonstrates that IPEV outperforms HTP (KNN) by ~0.21 on the AUC metric. HTP (QDA) yields the lowest AUC value with making arbitrary decisions. IPEV does not perform as well on real virome data as it does on simulated data, and it may be due to imbalanced sequence lengths and inaccurate labeling,

**Table S14.** Likelihood scores predicted by IPEV algorithm on capping enzymes

| Origin of enzyme, complete genome sequence, Genbank ID | | Likelihood Scores |
| --- | --- | --- |
| VV, [NC_006998.1](http://www.ncbi.nlm.nih.gov/nuccore/66275797) | 0.9740 | |
| Idem | 0.9828 | |
| Acanthamoeba Polyphaga Mimivirus, [NC_014649.1](http://www.ncbi.nlm.nih.gov/nuccore/NC_014649.1) | 0.9856 | |
| Bluetongue Virus, [NC_006024.2](http://www.ncbi.nlm.nih.gov/nuccore/NC_006024.2) | 0.9447 | |
| African swine fever virus, [NC_001659.2](http://www.ncbi.nlm.nih.gov/nuccore/NC_001659.2) | 0.9598 | |
| Bamboo Mosaic Virus, [L77962.1](http://www.ncbi.nlm.nih.gov/nuccore/L77962.1) | 0.9928 | |
| Kluyveromyces lactis plasmid pGKL2, [NC_010187.1](http://www.ncbi.nlm.nih.gov/nuccore/NC_010187.1) | 0.9986 | |

Table S14 highlights the efficacy of the IPEV algorithm when tested against a dataset of eukaryotic viral enzymes, originally identified and experimentally validated by Philippe et al [3]. The high likelihood scores predicted by IPEV underscore the robustness and reliability of our algorithm.

Table S15: P-values show the phageome's average coefficient of variation was significantly lower compared to the PPV (Wilcoxon rank-sum test, adjusted using the Benjamini-Hochberg correction).

| Subject | Unadjusted P-Value | Adjusted P-Value |
| --- | --- | --- |
| Subject 916 | 0.000674 | 0.002245 |
| Subject 917 | 0.004389 | 0.006270 |
| Subject 918 | 0.000674 | 0.002245 |
| Subject 919 | 0.043208 | 0.043208 |
| Subject 920 | 0.001522 | 0.003806 |
| Subject 921 | 0.002080 | 0.004161 |
| Subject 922 | 0.004389 | 0.006270 |
| Subject 923 | 0.010080 | 0.012600 |
| Subject 924 | 0.014882 | 0.016536 |
| Subject 925 | 0.000674 | 0.002245 |

**Supplementary Figures**


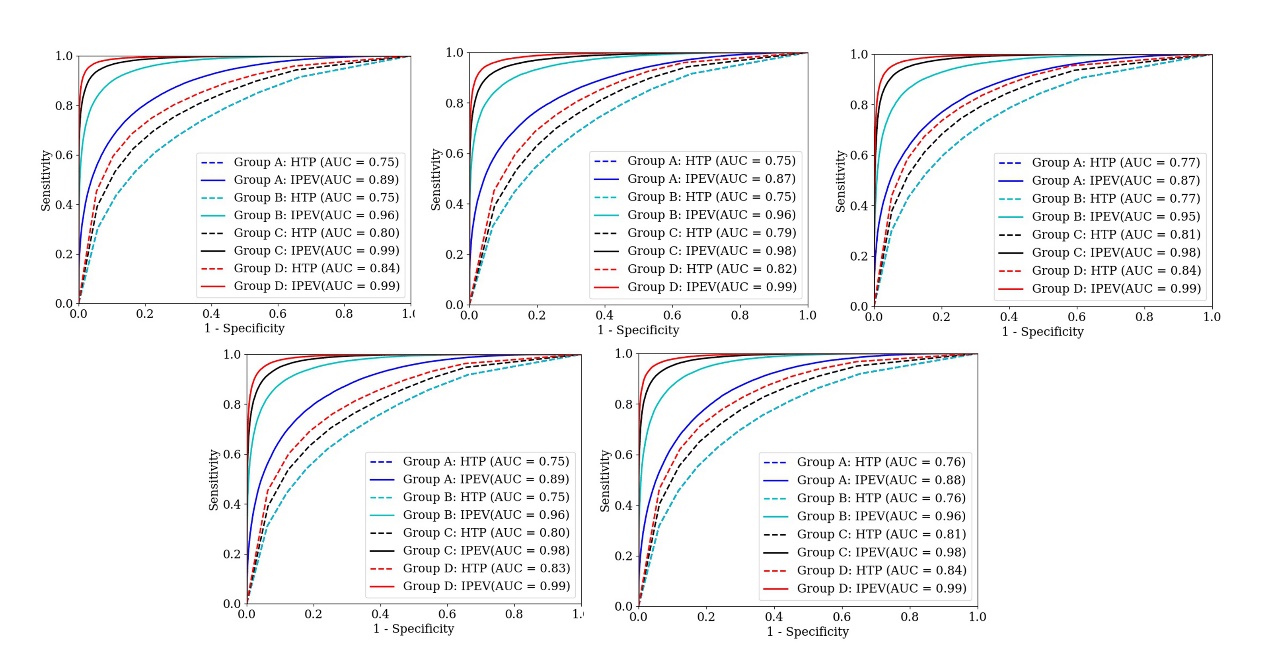


**Figure S1.** The ROC curves and AUC value of IPEV and HTP performances in each set of 5-fold cross-validation.


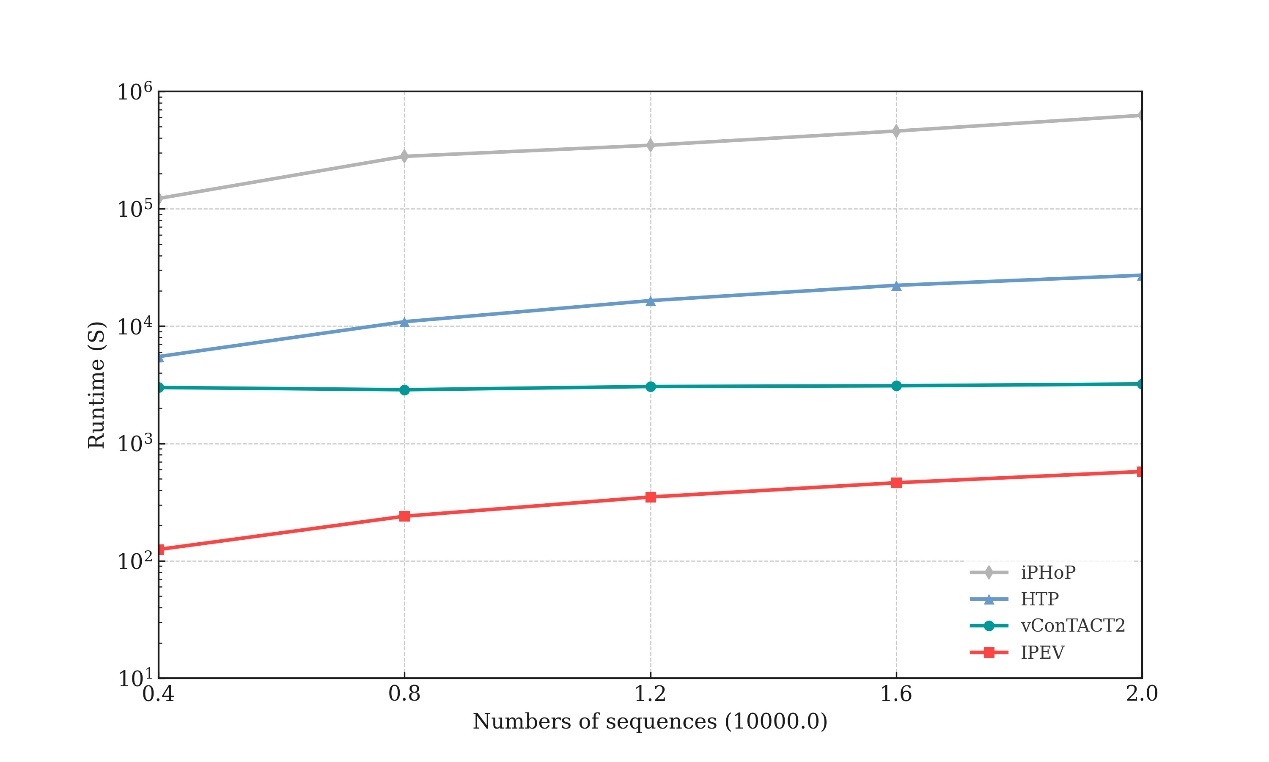

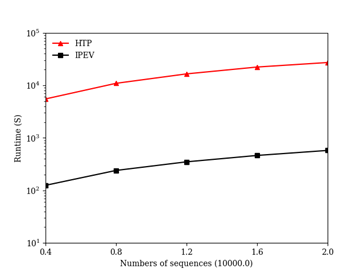


**Figure S2.** Performance of IPEV, HTP, vConTACT2 and iPHoP as the number of sequences increases under the same computing configuration (1,200-1,800 bp).

We utilized the MetaSim software to simulate sequences of varying lengths, ranging from 1,200 to 1,800 bp. These sequences were subsequently predicted using both the IPEV and related tools. We collected statistics on the running times of these tools under different quantities of sequences. As shown in Figure S2, IPEV operates 50 times faster than HTP, 30 times faster than vConTACT v.2.0, and 1,225 times faster than iPHoP, taking only 9.6 minutes to analyze 20,000 sequences of 1,200-1,800 bp when using the same computational resources (CPU: Intel(R) Xeon(R), 20 cores, GPU: NVIDIA Corporation GV100GL [Tesla V100 PCIe 32 GB]).

**
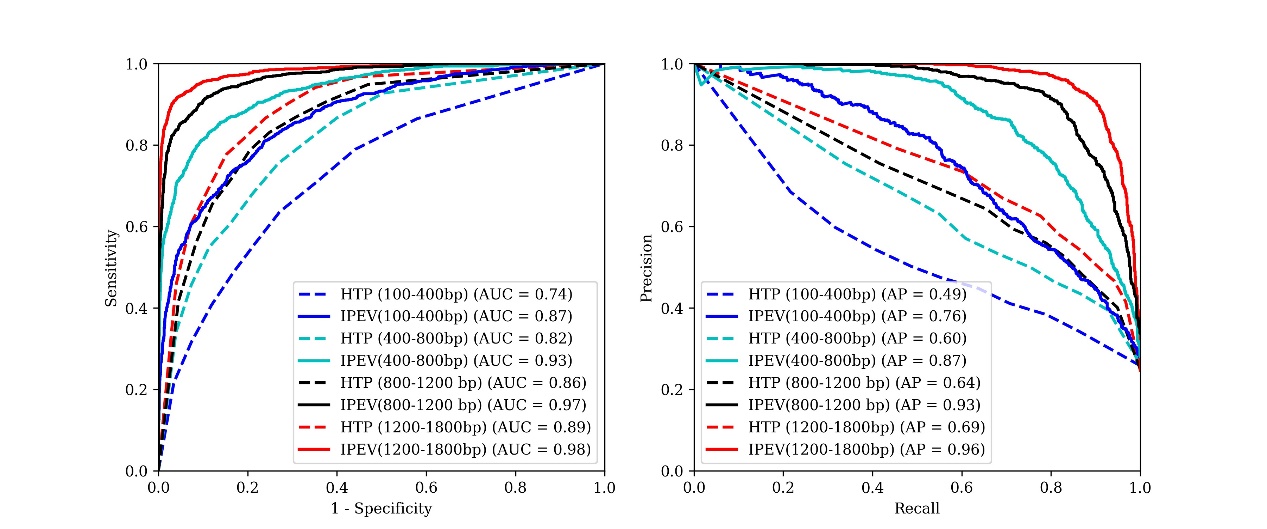
**

**Figure S3.** Comparison of IPEV and HTP on Dataset I2 (Query parameter: coverage=30%, identity=60%)

**
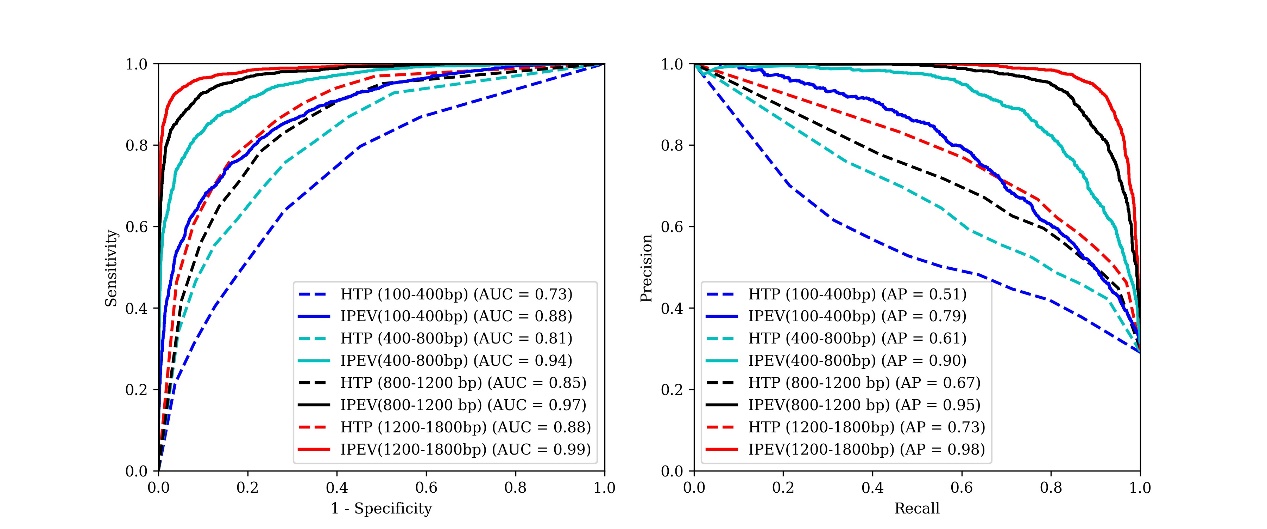
Figure S4.** Comparison of IPEV and HTP on Dataset I3 (Query parameter: coverage=60%, identity=30%)

**
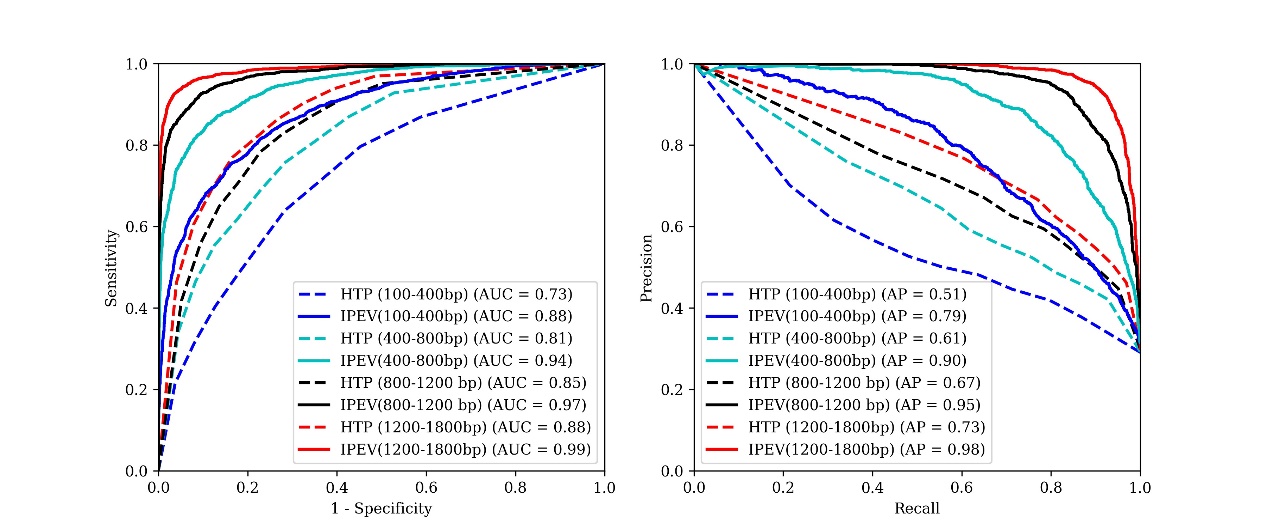
**

**Figure S5.** Comparison of IPEV and HTP on Dataset I4 (Query parameter: coverage=60%, identity=60%)


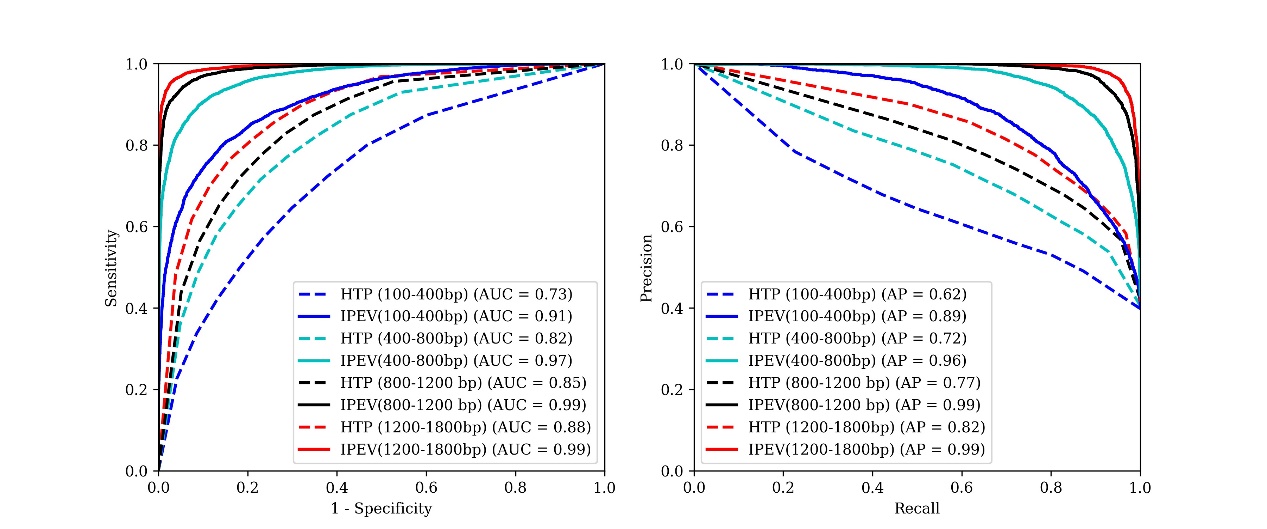
**Figure S6.** Comparison of IPEV and HTP on the Dataset I5 (Query parameter: coverage=100%, identity=30%)

**
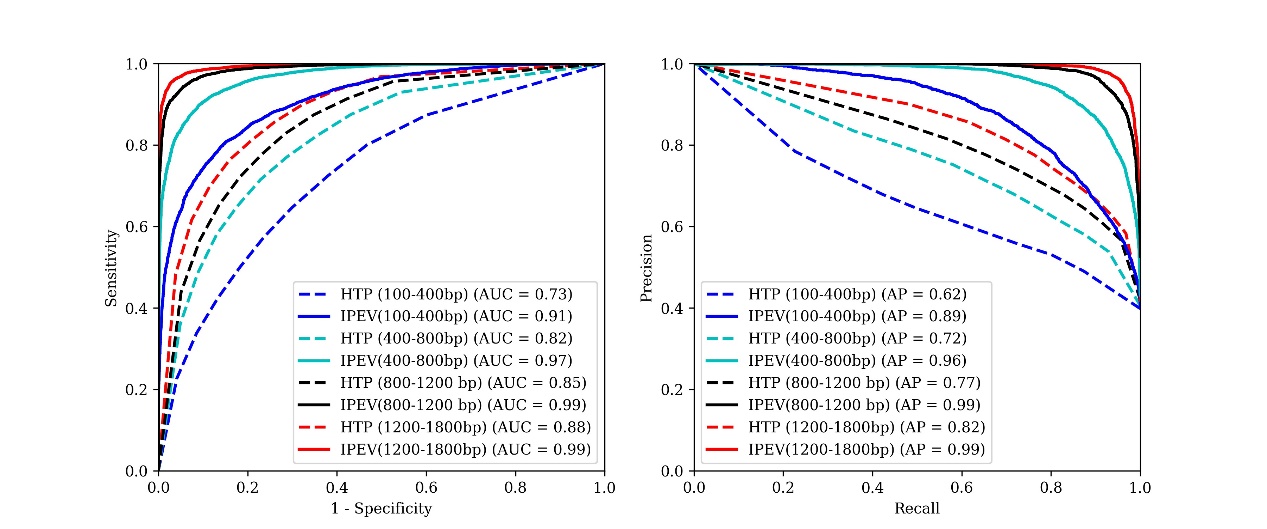
Figure S7.** Comparison of IPEV and HTP on Dataset I6 (Query parameter: coverage=100%, identity=60%)


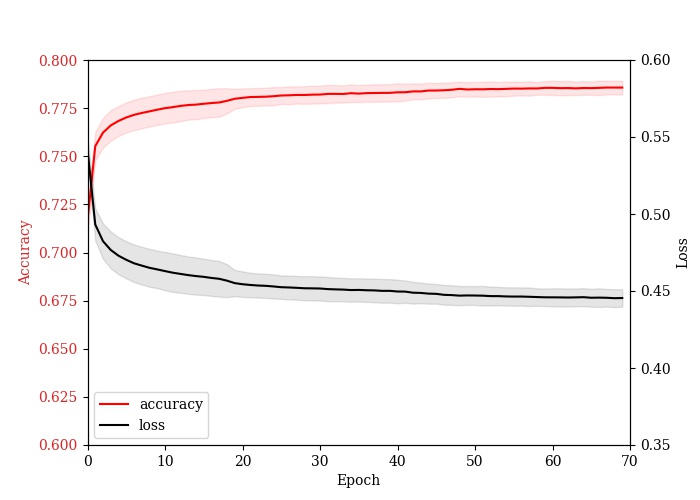


**Figure S8.** Mean accuracy, mean loss, and mean of IPEV’s 5-cross-validation on Group A


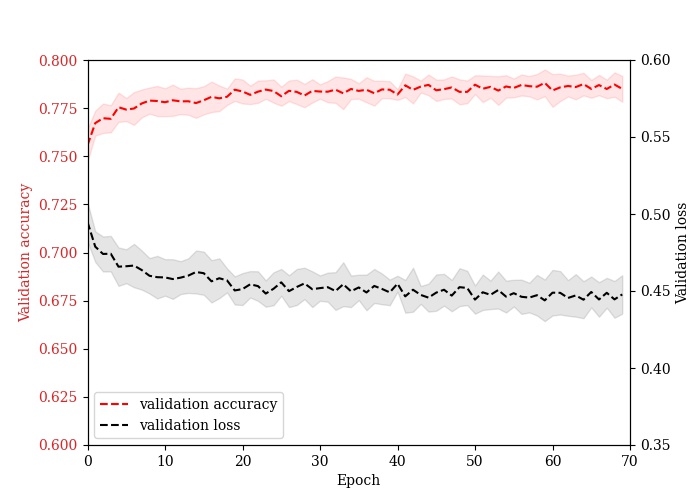


**Figure S9.** The mean validation loss and accuracy of IPEV’s 5-cross-validation on Group A.

**Figure S8.** and **Figure S9.** depicts the trajectory of accuracy and loss metrics throughout the training process of the IPEV neural network. Remarkably, around the 50th epoch, both the accuracy achieves a near peak and the loss hits a near nadir. And, during the entirety of the training process, the validation performance remains closely aligned with the training results.

**
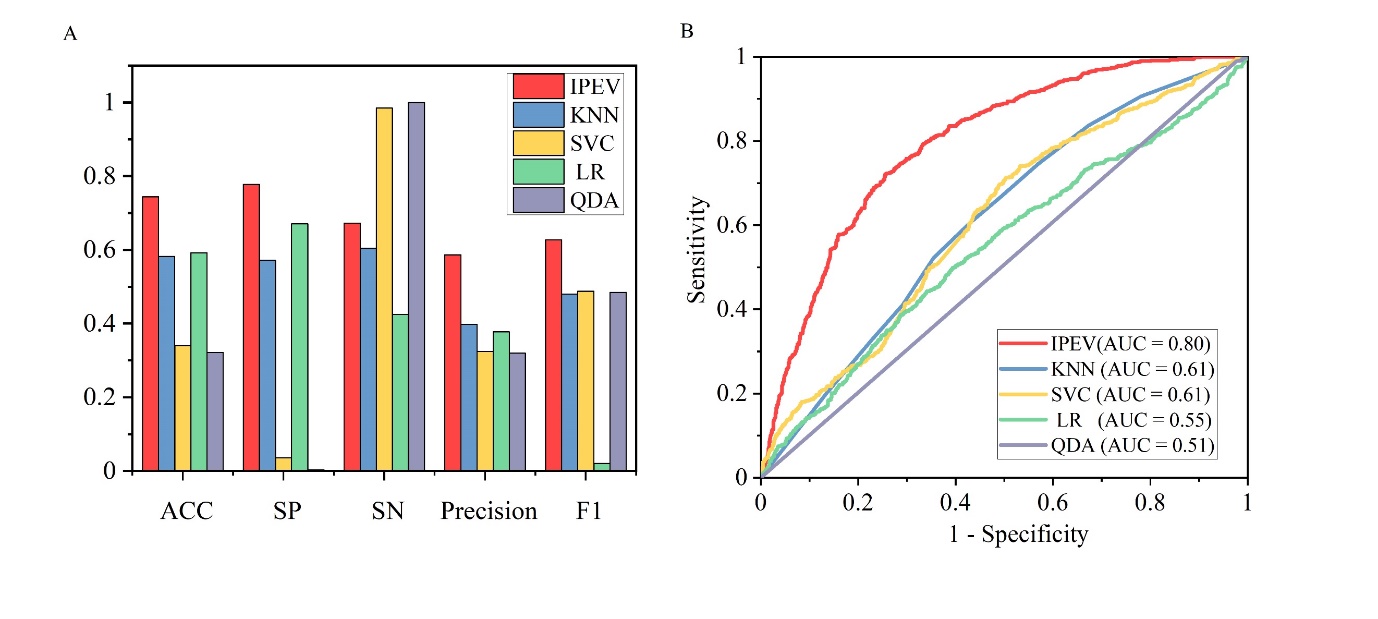
**

**Figure S10. A.** Performance (accuracy, specificity, sensitivity, precision, and F1-score) of IPEV and HTP (KNN, SVC, LR, QDA) on the duck gut virome data. **B.** The ROC curve and AUC value of IPEV and HTP (KNN, SVC, LR, QDA) on the duck gut virome data.


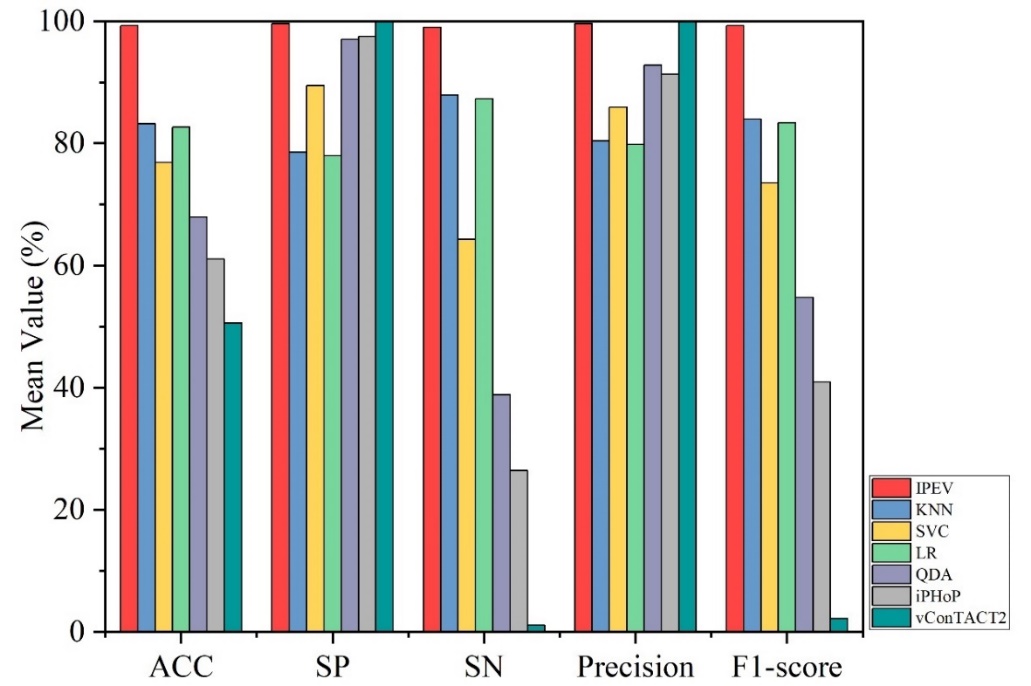


Figure S11. Average performance of IPEV, HTP, iPHoP, and vConTACT v.2.0 across three independent test sets with sequence lengths of 3,000-5,000 bp


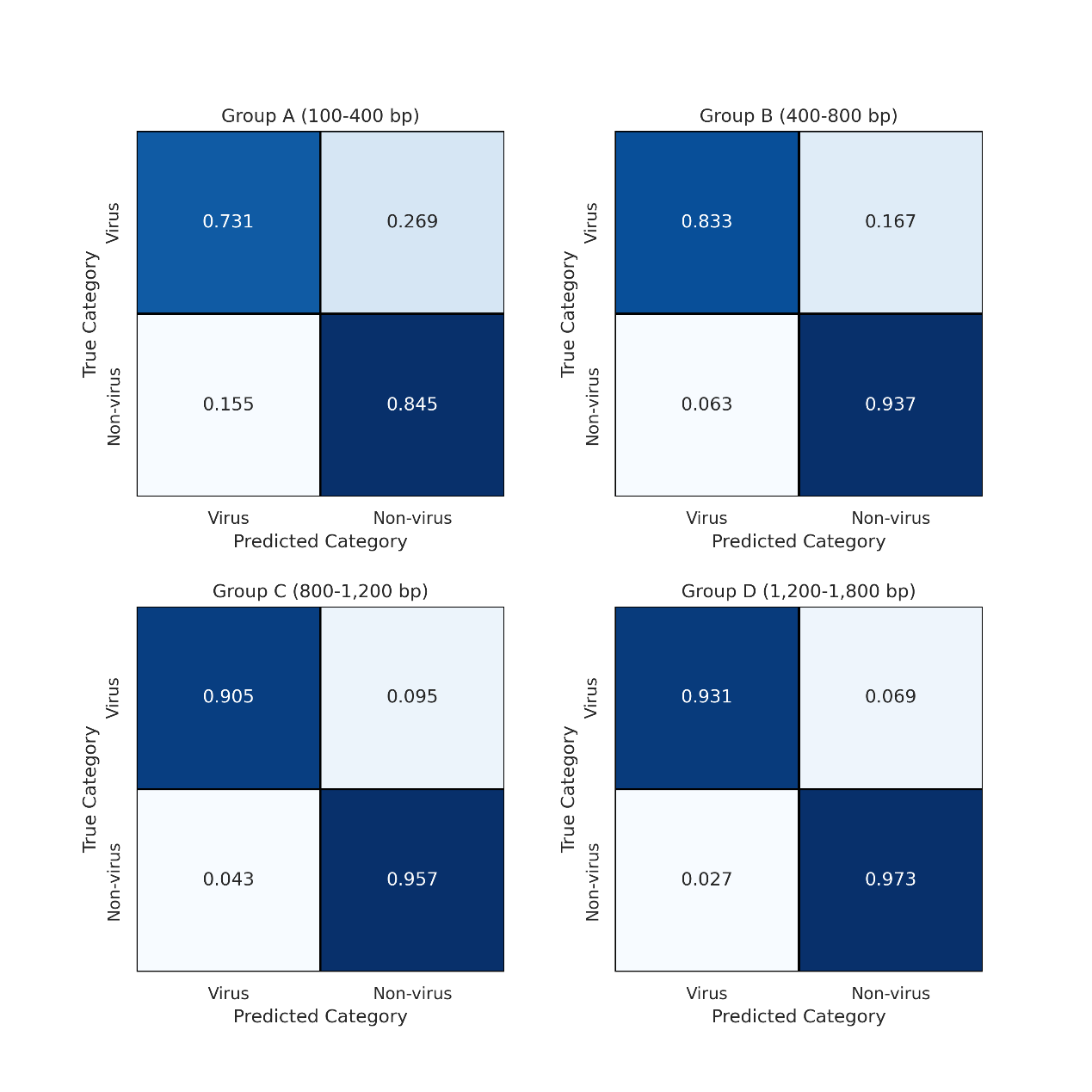


Figure S12. the confusion matrices show the false positive reduction capability of IPEV on Groups A-D in datasets where viruses and non-viruses are present in a 50:50 ratio.

**References**

1. Shkoporov AN, Clooney AG, Sutton TDS et al. The Human Gut Virome Is Highly Diverse, Stable, and Individual Specific, *Cell Host Microbe.* 2019;26(4):527-41.e5.

2. Fawaz M, Vijayakumar P, Mishra A et al. Duck gut viral metagenome analysis captures snapshot of viral diversity, *Gut Pathog.* 2016;8(30.

3. Jaïs PH, Decroly E, Jacquet E et al. C3P3-G1: first generation of a eukaryotic artificial cytoplasmic expression system, *Nucleic Acids Res.* 2019;47(5):2681-98.
