# Supplementary material for: IPEV: identification of prokaryotic and eukaryotic virus-derived sequences in virome using deep learning
Source: Gigascience. 2024 Apr 22;13:giae018. doi: 10.1093/gigascience/giae018 (PMC11034026; doi:10.1093/gigascience/giae018)
Supplement: giae018_Response_to_Reviewer_Comments_Original_Submission [file giae018_response_to_reviewer_comments_original_submission.pdf]

Author's Response To Reviewer Comments

Dear Editor,

Thank you very much for your previous Email on March 9, 2024, regarding our manuscript, “IPEV: identification of prokaryotic and eukaryotic virus-derived sequences in virome using deep learning” (GIGA-D-23-00357). We are very pleased to learn that our manuscript is acceptable for publication in GigaScience, subject to the completion of revisions suggested by the reviewer. Herein, we would like to express our gratitude to you, and the expert member of the Editorial Board, and Reviewer 1, for your thorough review. We are also pleased to note that our responses have addressed all of their concerns, that the “expert and the previous referee agree that the revisions are satisfactory”. Additionally, we are grateful for their careful reading of the previous version of our manuscript, and the further comments raised by the reviewer helped us make further improvements.

Following your suggestion, we have registered our new software application in the bio.tools database to receive a BioTools ID (biotools:IPEV), and we have included this ID in the latest version of the manuscript following the RRID.

In this revision, we made several minor changes (reported below) exactly followed Reviewer 1’s and your points. In addition, we have carefully reviewed the journal’s style and ensured that the revised manuscript conforms to it. At this stage, we have removed the tracking of changes or highlighting in red that was made for the purpose of peer review in the revised manuscript. We then report our revisions and point-by-point responses to Reviewer 1’s comments (in italic text) one by one as follows:

Reviewer 1：
Comment 1: The authors include some text in the Discussion section (paragraph from line 423 to line 436, and paragraph from line 437 to 448) that, in my opinion, would fit better in the Results section. I suggest the authors include these in the Results section, and then in the Discussion comment how those results compare to other methods and what are their implications.
We are deeply grateful for Reviewer 1's suggestions. Following the suggestion of Review 1, we have relocated the below sentences into Subsection “Performance on viral genome fragments using cross-validation” as follows:
“In addition, we plotted the training loss, validation loss, training accuracy, and validation accuracy curves with respect to the number of epochs using 5-fold cross-validation. We observed that the model converged at 30 epochs, with training and validation losses and accuracies remaining consistent and overlapping. This indicates that the model achieved high performance while avoiding overfitting or underfitting the data, as shown in Figures S8 and S9”. (Please refer to Line 232-236, Page 8 in the revised manuscript.)
“We also test IPEV’s effectiveness in differentiating viral from non-viral genome fragments in datasets where viruses and non-viruses are present in a 50:50 ratio. As shown in Figure S12, across Groups A to D, IPEV achieved Sn scores of 0.73, 0.833, 0.905, and 0.931, respectively (details on sample construction and methods can be found in Supplementary Materials and Methods).” (Please refer to Line 236-240, Page 8 in the revised manuscript.)

Comment 2: I would suggest modifying the sentence in line 42 like this: “Nonetheless, it is essential to note that enriched sample approaches carry the risk of losing valuable host or environmental information [8], potentially leading to inaccurate virus host identification and constraining subsequent analyses.”
We are very thankful for Reviewer 1’s comment. We have revised the sentence “Nonetheless, it is essential to note that enriched sample approaches carry the risk of losing valuable host or environmental information [8] and can cause inaccurate virus host identification, thereby constraining subsequent analyses.” as “Nonetheless, it is essential to note that enriched sample approaches carry the risk of losing valuable host or environmental information [8], potentially leading to inaccurate virus host identification and constraining subsequent analyses.” (Please refer to Line 42-44, Page 2 in the revised manuscript.)

Comment 3: In the sentence starting in line 392, instead of “During” use “For”.
We are very thankful for Reviewer 1’s comment. We have replaced “During” with “For”. Therefore, we have

revised “During artificial contigs with less than 30% sequence homology against the training set” as “For artificial contigs with less than 30% sequence homology against the training set.” (Please refer to Line 192, Page 14 in the revised manuscript.)

In hoping that the above revision has clarified all the points by Reviewer 1 and given a point-by-point response to all the concerns, we hereby resubmit our manuscript to the journal. We thank you for your kind consideration. Please do not hesitate to let us know if additional edits are needed. We will be no more than willing to meet your requirements.

Sincerely yours,
Huaiqiu Zhu, Ph. D., Professor
Peking University
