# Supplementary material for: IPEV: identification of prokaryotic and eukaryotic virus-derived sequences in virome using deep learning
Source: Gigascience. 2024 Apr 22;13:giae018. doi: 10.1093/gigascience/giae018 (PMC11034026; doi:10.1093/gigascience/giae018)
Supplement: giae018_Response_to_Reviewer_Comments_Revision_1 [file giae018_response_to_reviewer_comments_revision_1.pdf]

Author's Response To Reviewer Comments

Dear Editor,

Thank you very much for your previous Email on 21, Mar, 2024 regarding our manuscript, “IPEV: identification of prokaryotic and eukaryotic virus-derived sequences in virome using deep learning” (GIGA-D-23-00357R1). We have carefully addressed the editorial requests as outlined in your previous correspondence and have made the necessary corrections accordingly.

We revised the sentences in Section “Availability of data and materials” as:
“Our study contains only publicly available viral genome sequences and reference bacterial genome sequences [ENA study accession: PRJEB22493, NCBI BioProject accession: PRJNA545408]. The IPEV program and a detailed tutorial that can be publicly available at the Zhu Lab home page (https://cqb.pku.edu.cn/zhulab/info/1006/1156.htm) or GitHub (https://github.com/basehc/IPEV) under the GNU General Public License V3. An archival copy of the code is available via Software Heritage [62]. DOME-ML annotations are available via the DOME wizard [63]. Code and data for transparent and reproducible results were documented on Zenodo [64] and Docker image [65].”

“62. Yin H, Wu S, Tan J, Guo Q, Li M, Guo J, Wang Y, Jiang X, Zhu H . IPEV (Version 4). [Computer software]. Software Heritage, 2024, swh:1:snp:144cfb30531a50ea13d111b613eed96daf3497e0;origin=https://github.com/basehc/IPEV”
“63. DOME annotation: IPEV_data https://dome.ds-wizard.org/wizard/projects/5e3673f9-217c-474e-a5e7-91c6768638ad”
“64. Yin H, Wu S, Tan J, Guo Q, Li M, Guo J, Wang Y, Jiang X, Zhu H. Data from: Identification of prokaryotic and eukaryotic virus-derived sequences in virome using deep learning [Data set]. Zenodo. 2023 https://doi.org/10.5281/zenodo.10118192”
“65. Docker Image for IPEV. https://hub.docker.com/r/dryinhc/ipev_v1”

We also include ORCIDs for all authors on the title page in the revised manuscript.

We hope that these revisions meet your approval and look forward to the opportunity of our manuscript being published. Thank you for considering our work for GigaScience. Please do not hesitate to let us know if additional edits are needed. We will be no more than willing to meet your requirements.

Sincerely yours,
Huaiqiu Zhu, Ph. D., Professor
Peking University
