# Supplementary material for: IPEV: identification of prokaryotic and eukaryotic virus-derived sequences in virome using deep learning
Source: Gigascience. 2024 Apr 22;13:giae018. doi: 10.1093/gigascience/giae018 (PMC11034026; doi:10.1093/gigascience/giae018)
Supplement: giae018_Reviewer_1_Report_Original_Submission — Guillermo Andres Rangel-Pineros -- 1/22/2024 Reviewed [file giae018_reviewer_1_report_original_submission.pdf]

Reviewer Report

Title: IPEV: identification of prokaryotic and eukaryotic virus-derived sequences in virome using deep learning

Version: Original Submission **Date:** 1/22/2024

Reviewer name: Guillermo Andres Rangel-Pineros

Reviewer Comments to Author:

Yin et al described the development and testing of IPEV, a deep-learning-based model that detects and discriminates sequences derived from prokaryotic and eukaryotic viruses in virome datasets. The model was developed using a set of reference viral sequences with known host information. The sequences were represented as sequence pattern matrices that contained values derived from the frequency and order of trinucleotide pairs. These matrices were subsequently used to train a 2D convolutional neural network that generates a 2-value vector for each input sequence, indicating the probability that the sequence corresponds to a prokaryotic or eukaryotic virus. The model was trained and tested using 5-fold cross validation on the reference set, and the authors assessed the robustness of the method using input datasets covering a range of homology and mutation rate values. Finally, the authors applied their model to a gut virome dataset from Shkoporov et al 2019, and marine virome datasets from Gregory et al 2019. Indeed, IPEV represents a novel method that classifies viral sequences based on the type of host they target (prokaryotic or eukaryotic), and the results presented indicate that it efficiently covers a wide range of sequence lengths (from 100 bp). A model like IPEV provides a focus on eukaryotic viruses that is relatively shallow, in comparison with phages for which a wide range of prediction tools have been developed to date.In my opinion, the authors satisfactorily addressed the comments and suggestions made in the first round of review. I only have a few final suggestions to finalise the manuscript and have it ready for publication:1) The authors include some text in the Discussion section (paragraph from line 423 to line 436, and paragraph from line 437 to 448) that, in my opinion, would fit better in the Results section. I suggest the authors include these in the Results section, and then in the Discussion comment how those results compare to other methods and what are their implications.2) I would suggest modifying the sentence in line 42 like this: "Nonetheless, it is essential to note that enriched sample approaches carry the risk of losing valuable host or environmental information [8], potentially leading to inaccurate virus host identification and constraining subsequent analyses."3) In the sentence starting in line 392, instead of "During" use "For".

Level of Interest

Please indicate how interesting you found the manuscript: Choose an item.

Quality of Written English

Please indicate the quality of language in the manuscript: Choose an item.

Declaration of Competing Interests

Please complete a declaration of competing interests, considering the following questions:

- Have you in the past five years received reimbursements, fees, funding, or salary from an organisation that may in any way gain or lose financially from the publication of this manuscript, either now or in the future?
- Do you hold any stocks or shares in an organisation that may in any way gain or lose financially from the publication of this manuscript, either now or in the future?
- Do you hold or are you currently applying for any patents relating to the content of the manuscript?
- Have you received reimbursements, fees, funding, or salary from an organization that holds or has applied for patents relating to the content of the manuscript?
- Do you have any other financial competing interests?
- Do you have any non-financial competing interests in relation to this paper?

If you can answer no to all of the above, write 'I declare that I have no competing interests' below. If your reply is yes to any, please give details below.

I declare that I have no competing interests

I agree to the open peer review policy of the journal. I understand that my name will be included on my report to the authors and, if the manuscript is accepted for publication, my named report including any attachments I upload will be posted on the website along with the authors' responses. I agree for my report to be made available under an Open Access Creative Commons CC-BY license (http://creativecommons.org/licenses/by/4.0/). I understand that any comments which I do not wish to be included in my named report can be included as confidential comments to the editors, which will not be published.

Choose an item.

To further support our reviewers, we have joined with Publons, where you can gain additional credit to further highlight your hard work (see: https://publons.com/journal/530/gigascience). On publication of this paper, your review will be automatically added to Publons, you can then choose whether or not to claim your Publons credit. I understand this statement.

Yes Choose an item.
